# Measuring problem prescription opioid use among patients receiving long-term opioid analgesic treatment: development and evaluation of an algorithm for use in EHR and claims data

**DOI:** 10.1080/21556660.2020.1750419

**Published:** 2020-04-28

**Authors:** David S. Carrell, Ladia Albertson-Junkans, Arvind Ramaprasan, Grant Scull, Matt Mackwood, Eric Johnson, David J. Cronkite, Andrew Baer, Kris Hansen, Carla A. Green, Brian L. Hazlehurst, Shannon L. Janoff, Paul M. Coplan, Angela DeVeaugh-Geiss, Carlos G. Grijalva, Caihua Liang, Cheryl L. Enger, Jane Lange, Susan M. Shortreed, Michael Von Korff

**Affiliations:** a Kaiser Permanente Washington Health Research Institute, Seattle, WA, USA; b Kaiser Permanente Washington, Seattle, WA, USA; c Amazon, Seattle, WA, USA; d Kaiser Permanente Center for Health Research Northwest Region, Portland, OR, USA; e Purdue Pharma, L.P, Stamford, CT, USA; f Vanderbilt University, Nashville, TN, USA; g Optum, Inc., Epidemiology, Boston, MA, USA; h The Fred Hutchison Cancer Research Center, Seattle, WA, USA

**Keywords:** Algorithms, electronic health records, opioid-related disorders, population surveillance

## Abstract

**Objective:**

Opioid surveillance in response to the opioid epidemic will benefit from scalable, automated algorithms for identifying patients with clinically documented signs of problem prescription opioid use. Existing algorithms lack accuracy. We sought to develop a high-sensitivity, high-specificity classification algorithm based on widely available structured health data to identify patients receiving chronic extended-release/long-acting (ER/LA) therapy with evidence of problem use to support subsequent epidemiologic investigations.

**Methods:**

Outpatient medical records of a probability sample of 2,000 Kaiser Permanente Washington patients receiving ≥60 days’ supply of ER/LA opioids in a 90-day period from 1 January 2006 to 30 June 2015 were manually reviewed to determine the presence of clinically documented signs of problem use and used as a reference standard for algorithm development. Using 1,400 patients as training data, we constructed candidate predictors from demographic, enrollment, encounter, diagnosis, procedure, and medication data extracted from medical claims records or the equivalent from electronic health record (EHR) systems, and we used adaptive least absolute shrinkage and selection operator (LASSO) regression to develop a model. We evaluated this model in a comparable 600-patient validation set. We compared this model to ICD-9 diagnostic codes for opioid abuse, dependence, and poisoning. This study was registered with ClinicalTrials.gov as study NCT02667262 on 28 January 2016.

**Results:**

We operationalized 1,126 potential predictors characterizing patient demographics, procedures, diagnoses, timing, dose, and location of medication dispensing. The final model incorporating 53 predictors had a sensitivity of 0.582 at positive predictive value (PPV) of 0.572. ICD-9 codes for opioid abuse, dependence, and poisoning had a sensitivity of 0.390 at PPV of 0.599 in the same cohort.

**Conclusions:**

Scalable methods using widely available structured EHR/claims data to accurately identify problem opioid use among patients receiving long-term ER/LA therapy were unsuccessful. This approach may be useful for identifying patients needing clinical evaluation.

## Introduction

### Background

The federal government has declared the epidemic of opioid-related harms in the United States^1–4^ to be a public health emergency^5^, and a committee convened by the National Academy of Sciences Engineering and Medicine has concluded that a coordinated response will be needed to reverse the escalating prevalence of these harms^6^. Opioid surveillance, a key component in this response, is hampered by the absence of accurate, scalable surveillance methods for identifying patients with problem opioid use^7^^,^^8^. To date, most large-scale investigations of problem use have relied on International Classification of Diseases, Ninth Revision (ICD-9) diagnostic codes for opioid abuse (305.*), dependence or addiction (304.*) and/or poisoning (965.00, 965.02, 965.09, E850; Supplementary Appendix A)^9–15^ despite their poor sensitivity^16^^,^^17^. Recent research indicates some patients without formal diagnoses have clinical documentation of problem opioid use in encounter notes (e.g. discussion of opioid use disorder treatment options)^17^, suggesting that more sophisticated structured data algorithms might allow for more accurate identification of patients with problem opioid use.

This study is one of 11 post-marketing requirements (PMR) studies for extended-release, long-acting opioid analgesics (ER/LA).

### Objective

The objective of this study was to use a moderate amount of manually-curated gold standard data to develop a computable algorithm that accurately identified patients experiencing problem prescription opioid use, and to use this algorithm to generate gold standard data to support epidemiologic investigations among a collection of 11 PMR studies. In order to allow the resulting algorithm to be applied in very large healthcare data sets, inputs to the algorithm were restricted to structured health data such as diagnosis, procedure and medication codes that are widely available from medical claims records or their equivalent derived from electronic health records (EHRs). This study focuses on ER/LA recipients because it was conducted pursuant to a United States Food and Drug Administration (FDA) request to companies holding New Drug Applications for ER/LA opioids (as distinct from immediate-release opioids) to conduct post-marketing studies to assess the serious risks associated with long-term ER/LA use^18–20^. The study design was reviewed by a panel of experts at a two-day FDA public meeting in 2014^21^. The protocol (PMR 3033-7) is available at www.clinicatrials.gov^22^. Gold standard data generated using the algorithm developed in this study were to be combined with gold standard data on opioid-related overdoses developed in a companion study and used to investigate the incidence and epidemiology of problem opioid use and opioid-related overdose and death^23^ in a very large patient cohort combining data from Kaiser Permanente Northwest (KPNW), KPWA, Optum, and Tennessee Medicaid. As such, this study also contributes to an emerging literature on automated methods to determine patient phenotypes or case status in “big” healthcare data to support clinical, epidemiological and surveillance research without the need for expensive, sample-constraining manual chart review^24–26^.

Our operational definition of clinically-documented problem opioid use is described elsewhere^27^. Briefly, we define problem opioid use as a spectrum of behaviors and symptoms associated with the unhealthy use of prescription opioid medications. This definition includes, but does not require, clinically-documented evidence of the behavioral or physiological manifestations of substance use disorder as defined in the Diagnostic and Statistical Manual of Mental Disorders, version 5 (DSM-5). We prefer this more inclusive definition because (1) chart notes often lack details needed to support a rigorous clinical diagnosis of substance use disorder – even for patients with substance use disorders, and (2) the public health motivation for this research is not limited to clinically diagnosed opioid use disorder (OUD). By “clinically documented” we simply mean that the information is recorded in patient charts; this does not imply that a formal clinical diagnosis of substance use disorder has been made. We aimed to produce an algorithm with sensitivity ≥0.90 at a positive predictive value (PPV) ≥0.90. However, given the limitations of structured EHR/claims data we specified in advance minimally acceptable sensitivity of ≥0.75 at PPV ≥0.75. As a secondary objective, we compared our algorithm to a simple algorithm based on diagnosis codes commonly used in the scientific literature (Supplementary Appendix A)^9–15^.

## Methods

### Setting

The setting for this study was Kaiser Permanente Washington (KPWA, formerly Group Health Cooperative), where over 890,000 patients received outpatient care documented in an Epic EHR system^28^ during the study period, 1 January 2006 to 30 June 2015. Data used was limited to structured health data (including diagnosis, procedure and medication codes) widely available from medical claims records or its equivalent derived from EHRs (hereafter referred to as EHR/claims data). We deliberately focused on EHR/claims data so that the resulting algorithm could be applied in a wide variety of settings, including claims databases representing tens of millions of lives^29^. To the KPWA EHR data, we added claims data for outpatient, urgent, inpatient, and chemical dependence care received by KPWA patients outside KPWA. Medications for outside chemical dependence care were represented in the KPWA EHR. Encounter, diagnosis, procedure, and medication records were combined and transformed into the Sentinel Common Data Model (CDM, version 6)^30^^,^^31^, which is applicable to large sectors of the US population^32^. A research team at Kaiser Permanente Washington Health Research Institute had access to study patients’ complete outpatient (including primary and specialty care) EHR charts and manually reviewed this information to create reference standard data regarding the presence of documented signs of problem opioid use^27^.

### Study cohort and sample

Patients eligible for this study were ≥18 years of age by 1 January 2006 and had received ≥60 days’ supply of extended-release or long-acting (ER/LA) opioid analgesics (including transdermal or oral opioids and excluding buprenorphine) in any 90-day span during the study period (“long-term ER/LA”). We did not exclude patients exposed to ER/LA medications prior to the start of the study period (i.e. we studied a “prevalent user” cohort). We excluded patients receiving nursing home or hospice services during the study period. Study eligibility was independent of exposure to immediate-release (IR) opioids or the presence or absence of other conditions or diagnoses. Study patients were required to have ≥24 months of continuous enrollment, including ≥6 months prior to and ≥18 months following the first ER/LA dispensing in a patient’s earliest qualifying long-term ER/LA episode (the patient’s index date). We also required patients to have at least eight study quarters with EHR-documented encounters to assure opportunities for clinicians to observe and document patient issues.

Our stratified random sample of 2,000 patients was enriched with patients 18–35 years of age and patients with diagnoses during the study period of opioid dependence, abuse, and/or poisoning (Supplementary Appendix A), both of which are known correlates of problem opioid use^9^^,^^33–35^. We randomly assigned 70% (*n* = 1,400) to an algorithm training set and reserved 30% (*n* = 600) for a one-time evaluation of the final algorithm. Assuming a 20% prevalence of problem use and algorithm performance of 80% sensitivity and 80% specificity, the 95% confidence intervals for sensitivity and specificity in this validation set would be 71–89% and 76–84%, respectively.

### Reference standard

The creation of reference standard data by manual chart review is described elsewhere^27^. Briefly, experienced chart abstractors following a written protocol manually reviewed each patient’s entire outpatient chart to determine whether signs of problem opioid use were clinically documented, and if so the earliest date of documentation (“onset date”). Determinations regarding problem use were based on the totality of the evidence in the chart; determinations were negative if evidence was weak or ambiguous^27^. Inter-rater reliability among charts receiving a single review was high (Cohen’s kappa = 0.83).

### Algorithm development

Each patient’s EHR and claims data were the source data for algorithm development. A study team of clinicians, epidemiologists and medical records experts formed operational definitions of a large number of candidate predictor variables using training data informed by findings reported in the literature^8^^,^^36–40^, clinical experience, and qualitative insights gained from the manual review of 80 charts comparable to but not included in the study sample. Candidate predictors were typically binary (yes/no) measures reflecting patient demographics, diagnoses, encounters, and utilization data elements, individually or in combination.

To gauge potential “signal” in individual candidate predictors we calculated the following risk ratio (RR):

$$\mathrm{RR}=\frac{\text{Percentage ofproblem use POSITIVES with predictor set to TRUE}}{\text{Percentage of problem use NEGATIVES with predictor set to TRUE}}$$

We considered candidate predictors with larger values of RR and larger numbers of patients positive for the predictor (or, for interval level predictors, above a reasonable cut-point) to indicate greater discriminating signal. Using this information, we iteratively refined candidate predictors. We used a similar analytic approach to dichotomize some continuous candidate predictors. We included age-group interactions with candidate predictors when such interactions were scientifically compelling.

We used adaptive least absolute shrinkage and selection operator (LASSO) logistic regression^41^^,^^42^, as implemented in the “lqa” R package^43^ to identify a subset of candidate predictors for the final algorithm. We used adaptive LASSO because we wanted a parsimonious and transparent prediction model. Traditional LASSO is a regression analysis method that selects predictors by penalizing, or “shrinking toward zero,” coefficients of candidate predictors that do not substantially improve algorithm accuracy; adaptive LASSO extends traditional LASSO by favoring predictors with stronger initial associations with the outcome^44^. Implementing adaptive LASSO requires a gamma parameter, which is an exponent applied to the coefficient weights that determine how much the initial estimates of associations with the outcome influences the model fitting, and a lambda parameter, which influences how sparse the final model will be. We used the inverse of the absolute value of coefficients obtained from ridge regression to estimate lambda coefficient weights as is recommended when the ratio of predictors to sample size is large^43^.

To select the parameter values, we used eight-fold cross-validation on the training data, performing a grid search over values of both gamma and lambda. We avoided smaller folds because they may lack enough events to estimate a rich model. Our metric for evaluating model fit given lambda and gamma was the sum of squares in the left-out portion of the cross-validation sample: $\sum_{i}^{n}{\left(y_{i}-{\hat{y}}_{i}\right)}^{2},$ where ${\hat{y}}_{i}$ is the predicted value of the *i*^th^ data point in the left-out portion of the cross-validation sample using the prediction model estimated in the cross-validation sample. After selecting both lambda and gamma using cross validation, we estimated the predictive model on the entire training set using adaptive LASSO with these lambda and gamma values; this produced the model for the final classification algorithm, which predicted the logit of the probability of chart-documented problem use as a linear combination of the retained terms, plus selected interactions between these. The model-specified (“fitted”) probability was used as a risk score for each patient. Because both training and validation data oversampled higher-risk patients, we calculated weights based on the inverse of each patient’s probability of selection^45–47^ (i.e. design weights) to reweight the analytic datasets back to the pool of eligible patients to estimate prevalence.

### Observation period for algorithm implementation

Performance of claims-based algorithms may improve as the data collection period increases^12^, but the duration of continuous enrollment may vary considerably across the diverse healthcare settings where this algorithm was intended to be used^48^^,^^49^. We, therefore, used a 36-month observation period, including 12 months before and 24 months after a patient’s ER/LA index date, because >50% of study-eligible KPWA, KPNW, Optum/Humedica, and Tennessee Medicaid (settings where the algorithm was to be applied) had ≥36 months of continuous enrollment. This period allowed for adequate capture of patient information without bias toward patients with longer enrollment. Including 12 months pre-index allowed us to assess patients’ experience prior to long-term ER/LA use.

We operationalized reference standard outcomes to reflect the 36-month observation period. Patients with signs of problem use before or during the 36-month period were considered positive, and patients without evidence or whose onset occurred after the 36-month period were considered negative.

### Algorithm evaluation

During algorithm development and for final evaluation we used cut points on algorithm-calculated risk scores to classify patients as positive (values at or above the cut point) or negative (all other values) for problem use. We did this for selected cut-points chosen to optimize performance with (a) desirable sensitivity, (b) desirable specificity, (c) desirable PPV, or (d) balanced sensitivity and PPV. All cut points were selected based on training data. To evaluate the final algorithm, we used these cut points and reported algorithm performance in validation data by comparing. algorithm classifications to reference standard classifications.

Our algorithm evaluation metrics were:

- Sensitivity (recall or true positive rate):
  true positives/(true positives + false negatives),
- Specificity (true negative rate):
  true negatives/(false positives + true negatives),
- Positive predictive value (PPV or precision):
  true positives/(true positives + false positives), and
- Negative predictive value (NPV):
  true negatives/(true negatives + false negatives).

We characterize tradeoffs in algorithm sensitivity and specificity graphically using receiver operating characteristic (ROC) curves.

To compare the final algorithm’s performance to an approach commonly reported in the literature, we operationalized a simple ICD-9 code-based algorithm which classified a patient positive if they had an ICD-9 diagnosis code for prescription opioid dependence, abuse, or poisoning (Supplementary Appendix A) at any time during the observation period and negative otherwise.

This study was approved by the Human Subjects Review Board of Kaiser Permanente Washington.

## Results

The study sample and manual chart review results are described elsewhere^27^. Briefly, 3,728 patients met the study inclusion and exclusion criteria (Table 1). Median total days’ supply of ER/LA medications dispensed during each patient’s earliest qualifying continuous enrollment period was 1,208 days (interquartile range [IQR] 257–1,837 days; range 60–6,684 days). The median age was 52 years (IQR: 44–60, range: 20–96), 55% were women, and 79% were white (Table 1). The prevalence of reference-standard problem use at any time during the 9.5-year study period, weighted to account for sampling probabilities, was 29.3%, and 23.0% when limited to the 36-month observation period used for algorithm evaluation.

**Table 1.** Demographic characteristics of study-eligible Kaiser Permanente Washington patients (*n* = 3,728), patients sampled for inclusion in Study 3B (*n* = 2,000), and patients randomly assigned to the training (*n* = 1,400) and validation (*n* = 600) samples.

	Eligible for study		Full study sample		Training sample		Validation sample	
Demographic characteristic	*n*	%	*n*	%	*n*	%	*n*	%
Number of patients	3,728	100%	2,000	100%	1,400	100%	600	100%
Age at ER/LA index date								
Mean (SD)	55 (13.4)		52 (13.4)		52 (13.3)		52 (13.6)	
Min	20		20		20		20	
Median	52		52		52		51	
Max	96		96		96		94	
18–34 years	229	6.1	229	11.5	159	11.3	70	11.7
35–54 years	1,734	46.5	958	47.9	662	47.3	296	49.3
55–64 years	1008	27.0	484	24.2	346	24.7	138	23.0
65 + years	757	20.3	329	16.5	233	16.6	96	16.0
Gender								
Female	2,046	55	1,096	55	763	55	333	56
Male	1,682	45	904	45	637	45	267	45
Race								
White/Caucasian	2,978	79.8	1,586	79.3	1,107	79.1	479	79.8
Black/African American	143	3.8	73	3.7	54	3.9	19	3.1
Native American/Alaska Native	120	3.2	69	3.5	46	3.3	23	3.8
Asian	69	1.8	31	1.6	23	1.6	8	1.3
Hawaiian/Pacific Islander	20	0.5	11	0.6	8	0.6	3	0.5
Unknown/not specified	398	10.6	196	11.5	162	11.5	68	11.5

We operationalized 1,126 candidate predictor variables. Briefly, these included demographic measures; the Charlson Comorbidity Index; other medication; medications used to treat opioid use disorder; diagnoses of pain, mental health conditions, other substance use/disorders, and opioid overdose; emergency room utilization; physical therapy utilization; measures characterizing opioid prescription fill patterns and morphine-equivalent dose; and a variety of clinically-relevant interaction terms (summarized in Table 2; details in Supplementary Appendix C). Our candidate predictors did not include the administration of naloxone. This was because we found, in a companion study of opioid overdose, that naloxone is often not captured in structured EHR data and, in any case, is often administered presumptively by emergency care personnel before opioid involvement is assessed, thereby reducing the predictive power of naloxone administration^50^. A plurality of candidate predictors characterized opioid dispensing. For example, one such predictor indicated whether a patient received during any 3-month period ≥3 partially overlapping IR dispensing with ≤14 days’ supply on a Saturday, Sunday, or Monday. Information about encounters and non-opioid medications were also commonly represented in predictors. Some predictors were created by varying the values of key elements if doing so preserved face validity (e.g. morphine equivalent dose [MEQ] of ≥33% versus ≥50% versus ≥75% over consecutive calendar quarters).

**Table 2.** Categories of 1,126 candidate predictor variables operationalized from Sentinel demographics, encounters, diagnoses, procedures and medications EHR/claims data considered for inclusion in the classification algorithm to identify patients with chart-documented problem opioid use.

Category	Operationalization notes^a^
Diagnoses	
Pain Diagnoses	Back pain, other back or neck disorder, headache or migraine, neuropathic pain, fibromyalgia, arthritis
Change in pain location over time	Change during various time intervals (days, weeks, months)
Count of distinct pain locations	Lower back, other back or neck disorder, headache or migraine, neuropathic pain, fibromyalgia, arthritis
Mental health disorders	Depression, bipolar disorder, anxiety disorder, other mental health disorders, other mood disorder, schizophrenia/schizoaffective
Problem opioid use	Dependence, abuse, poisoning (excluding heroin), heroin
Non-opioid substance use disorder	Alcohol disorder, specified drug dependence, cannabis dependence, combination of drug dependence, nondependent drug abuse, tobacco use disorder
Sleep disorder	Insomnia, psychophysiological insomnia, inadequate sleep hygiene, insomnia due drug or substance, insomnia due to medical condition, physiologic (organic) insomnia, hypersomnia of central origin, central sleep apnea syndrome, isolated sleep symptoms, concurrent use of opioids and insomnia diagnosis
Psycho-social trauma	Post-Traumatic stress disorder (PTSD), domestic violence (E-codes, V-codes)
Hepatitis/cirrhosis	Ever/never; counts (overall, by month, by quarter); percent of quarters
Endocarditis	Ever/never; counts (overall, by month, by quarter); percent of quarters
Comorbidities	Charlson comorbidity index; point in time and change over time
Accidental injury or poisoning due to drugs (E-codes)	Opioids, non-narcotic analgesics, barbiturates and sedatives, psychoactive medications, other drugs
Adverse Effects from psychoactive drugs (E-codes)	Ever/never; counts (overall, by month, by quarter); percent of quarters
Medications	
Days’ supply	Total days’ supply overall, per month, per quarter; ER/LA and SA/IR combined and by type; percent change in days’ supply over time; ever/never and count of quarters with excess days’ supply
Medications used for the treatment of substance use disorder	Total days’ supply overall, per month, per quarter; ever/never use at various points in time and relative to index date
Opioid dispensings	Ever/never by month, by quarter; counts overall, by month, by quarter; in proximity with other medication dispensings (days, weeks, quarters); by day of the week
Psychoactive medications	Various versions, including antidepressant medications, antianxiety medications, muscle relaxers, homeopathic dispensings, benzodiazepine, barbiturate, hypnotics, anticonvulsants, add medication, lithium, stimulants
Concomitant use of opioids and other psychoactive medications	Ever/never; counts (overall, by month, by quarter); percent of quarters; number of different medications used concomitantly
Overlapping dispensings ("early fills")	Ever/never; counts (overall, by month, by quarter); percent of quarters; operationalized in a variety of ways including by NDC, by opioid type, by day of the week and other characteristics of dispensings
Morphine equivalence dosing (MEQ or MED)	Various versions, including average daily meq, meq per day of supply, changes in meq over time, high meq by dispensing and by time period (month, quarter), by opioid type (short acting versus long acting)
Medications used to treat opioid use disorder	Total days’ supply overall, per month, per quarter; ever/never use at various points in time and relative to onset date; frequency of dispensings
Concurrent use of opioids and pain diagnosis	Ever/never; counts (overall, by month, by quarter); percent of quarters
Encounters	
Emergency room (ER) encounters	Various versions, including opioids dispensed on the same date as emergency room encounters, day of week, ever/never and count of emergency room encounters during opioid use, emergency room encounters during concomitant use of opioids and other psychoactive medication(s)
Procedures	
Treatment of substance use disorder	Ever/never; counts (overall, by month, by quarter); percent of quarters
Urine drug screening	Ever/never; counts (overall, by month, by quarter); percent of quarters; number of urine drug screen in close proximity to other risk indicators such as overlapping dispensings and high MEQ
Surgery	Various version, based on type, opioid use prior to and after surgery, diagnoses in close proximity to surgery
Combinations and interactions	
Combinations of data from multiple sources	Various versions, including frequency of urine drug screening during periods of overlapping opioid dispensings, emergency room encounters during periods of overlapping opioid dispensings, emergency room encounters during periods of excess days’ supply of opioids, emergency room encounters during concomitant use of opioids and other psychoactive medications, emergency room encounters during periods of high morphine equivalence dose
Interactions	Over 100 interaction terms including interactions with patient age, patient gender, and interactions between selected diagnoses

^a^ Most potential predictors were derived in a variety of ways in both continuous and binary forms, including but not limited to: ever/never, frequency (overall, by month, by quarter), percent of time or visits, and/or in combination with other variables.

The final adaptive LASSO model incorporated 53 of the 1,126 candidate predictors. These 53 predictors (Supplementary Appendix B) included age, sex, diagnosis of opioid-dependence; diagnoses of comorbidities including mental health disorders, alcohol use disorder, non-opioid drug dependence, tobacco use disorder and anxiety disorder; various measures of opioid dispensings based on days’ supply and MEQ; dispensing of opioids concomitantly with other medications such as benzodiazepines; various measures of early refills; opioid dispensing in proximity to ER encounters; the history of receiving medications used to treat drug dependence; the coincidence of urine drug screening and dispensing of opioid medications; pain diagnoses; and interaction terms based on patient age.

**Figure 1.**
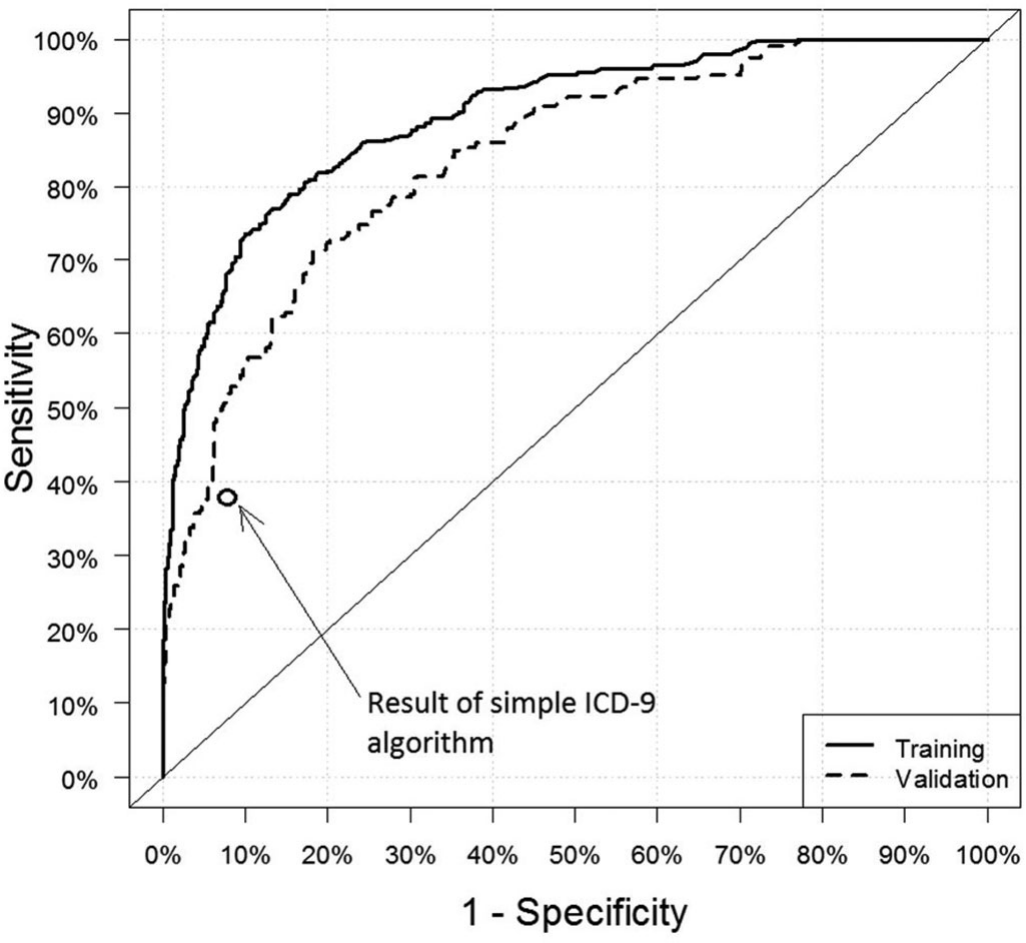
Receiver operating characteristic (ROC) curve for the problem opioid use classification algorithm in the training set (solid line), validation set (dashed lines), and sensitivity and specificity of the simple binary algorithm based on ICD-9 diagnosis codes for opioid abuse, dependence and poisoning (circle).

The performance of the final classification model is summarized in Table 3 and Figure 1. Performance in training data where algorithm sensitivity and PPV were balanced was 0.706 and 0.703, respectively, decreasing to 0.582 and 0.572, respectively, in validation data (Table 3, row 10), well below our a priori minimally acceptable level. A risk score cut point with high sensitivity (0.900 in training data and 0.850 in validation data; Table 3, row 1) yielded modest PPV (0.429 in training data and 0.412 in validation data). Conversely, a risk score cut point with high PPV (0.900 in training data and 0.774 in validation data; Table 3, row 7) yielded low sensitivity (0.356 in training data and 0.296 in validation data). The ROC curve (Figure 1) reveals consistent tradeoffs between sensitivity and specificity throughout the range of scores.

**Table 3.** Problem opioid use classification algorithm performance in the 1,400-patient training set and the 600-patient validation set, for selected values of the algorithm-generated risk score with desired performance characteristics (based on training data), as measured by sensitivity, specificity, positive predictive value (PPV) and negative predictive value (NPV).

Row	Desired performance characteristic (based on training data)		Risk score cut-point	Sensitivity^†^		Specificity^‡^		PPV^§^		NPV^¶^		Pred. prevalence^¥^	
				Train.	Valid.	Train.	Valid.	Train.	Valid.	Train.	Valid.	Train.	Valid.
1	Sensitivity	Excellent (0.90)	0.122	0.900	0.850	0.641	0.640	0.429	0.412	0.955	0.935	56%	56%
2		Good (0.80)	0.229	0.800	0.729	0.827	0.786	0.581	0.503	0.933	0.907	40%	42%
3		Acceptable (0.75)	0.278	0.752	0.629	0.879	0.841	0.651	0.541	0.922	0.884	35%	35%
4	Specificity	Excellent (0.90)	0.311	0.736	0.620	0.900	0.867	0.688	0.580	0.919	0.885	32%	33%
5		Good (0.80)	0.202	0.821	0.738	0.800	0.764	0.551	0.481	0.937	0.907	43%	44%
6		Acceptable (0.75)	0.169	0.861	0.776	0.751	0.727	0.509	0.457	0.948	0.916	47%	48%
7	PPV	Excellent (0.90)	0.705	0.356	0.296	0.988	0.974	0.900	0.774	0.837	0.823	14%	13%
8		Good (0.80)	0.478	0.545	0.486	0.959	0.934	0.800	0.685	0.876	0.859	22%	23%
9		Acceptable (0.75)	0.393	0.629	0.544	0.937	0.905	0.750	0.631	0.894	0.870	26%	28%
10	Sensitivity and PPV are balanced		0.330	0.706	0.582	0.911	0.871	0.703	0.572	0.912	0.875	30%	31%

† Sensitivity is the proportion of people correctly classified as having problem opioid use by the algorithm, defined as: Number of people identified with chart review to have problem opioid use and correctly classified by the algorithm to have problem opioid use/the number of people identified with chart review to have problem opioid use.

‡ Specificity is the proportion of people correctly classified as not having problem opioid use by the algorithm, defined as: Number of people identified with chart review to not have problem opioid use and correctly classified by the algorithm to not have problem opioid use/the number of people identified with chart review to not have problem opioid use.

§ Positive predictive value is the proportion of people the algorithm classifies as having problem opioid use who have problem opioid use identified by chart review, defined as: Number of people identified with chart review to have problem opioid use and classified by the algorithm to have problem opioid use/the number of people identified to have problem opioid use by the algorithm.

¶ Negative predictive value is the proportion of people the algorithm classifies as not having problem opioid use identified by chart review, defined as the number of people identified with chart review to not have problem opioid use and classified by the algorithm to not have problem opioid use/the number of people identified to have problem opioid use by the algorithm.

¥ This is the unadjusted predicted prevalence, defined as the percent of patients in the training sample predicted to be problem opioid use positive using the corresponding risk score cut point. The unadjusted prevalence of problem opioid use positive patients in the training sample was 36.5% (511/1,400).

The simple ICD-9 algorithm yielded a sensitivity of 0.399, PPV of 0.599, a specificity of 0.922 and a negative predictive value of 0.836 (Figure 1).

## Discussion

Our algorithm to detect clinician-documented signs of problem prescription opioid use based on a rich set of candidate predictors derived from medical claims data performed better than commonly used algorithms based on a simple set of ICD-9 diagnosis codes. However, performance in a cohort of long-term ER/LA opioid recipients was below our minimally acceptable level and not, therefore, suitable for gold standard case identification in epidemiologic investigations. If the balanced sensitivity/PPV version of the algorithm were used to classify patients it would overlook over 40% of actual cases, and 40% of patients classified as having problem use would be wrongly classified. Versions of the algorithm that preserved sensitivity would severely sacrifice PPV and vice-versa.

Despite its shortcomings for generating gold standard data, the modeling approach used here may be useful for developing clinical screening algorithms applicable to *all recipients of long-term opioid therapy* (not just ER/LA recipients) needed to identify patients at elevated risk of developing problem opioid use^51^. Such algorithms would use a patient’s EHR data *preceding an upcoming encounter* to calculate risk as of that encounter (rather than using data before and after ER/LA initiation, as in the present algorithm). To limit false-positive classifications, a problem opioid use risk score would be calibrated to emphasize specificity (rather than sensitivity), as is common in screening efforts to avoid high false-positive rates^52^^,^^53^.

We can speculate about possible reasons for the limited success of this algorithm. First, though it was not anticipated when this study was planned in 2014, focusing on a prevalent ER/LA user cohort, most of whom had substantial exposure to prescription opioids *prior to their study index dates*, may have severely complicated the algorithm development task. By not beginning observation at patients’ first exposure to long-term opioid therapy (including immediate-release formulations) the indicators of cause and effect related to problem use may have been confounded, increasing perplexity during algorithm training. It is possible, for example, that clinicians may have transitioned some patients to ER/LA therapy because of concerns about problematic use, a reasonable strategy given reports that ER/LA formulations carry reduced abuse/addiction potential^54^^,^^55^. Such channeling bias may also have inflated the observed prevalence of problem use.

Second, and also unanticipated when this study was planned, structured EHR/claims data alone may lack the nuance required to accurately identify signs of problem opioid use, a highly complex phenomenon^56^^,^^57^. To accurately identify this outcome algorithmically, it may be necessary to incorporate richer EHR data, including information from unstructured chart notes, thereby precluding the algorithm’s use in medical claims databases. Previous attempts to identify patients experiencing problem opioid use have yielded varying results^7^^,^^58^. Multiple screening tools have been developed^8^, but alternative approaches have sometimes given discordant results^59^. Distinguishing among subgroups of patients receiving long-term opioid therapy – based on age group, comorbidity profiles, or coterminous use of medications that amplify risks such as benzodiazepines – rather than attempting to use a single algorithm to identify all patients with problem use may improve algorithm performance. It is possible that more detailed diagnostic coding in the ICD-10 era (which began after our study period) may contain additional useful information.

Limitations of this study should be noted. First, we used professional chart abstractors rather than clinicians to create the reference standard, and some may consider clinician review to be superior. However, inter-rater agreement, the most objective indicator of high-quality abstraction, was very strong in this study and abstraction was guided by a detailed protocol^27^. Second, while adaptive LASSO is an appropriate method when candidate predictors exceed the number of outcome events, it is possible other modeling methods such as neural networks may have yielded somewhat better results. Third, this work was conducted in a single site; results elsewhere may vary. It is noteworthy that in a companion study of opioid overdose, the performance of an opioid overdose algorithm developed at Kaiser Permanente Northwest, which was very good, performed very similarly in Optum claims data, Medicaid data for the State of Tennessee, and Kaiser Permanente Washington^50^.

## Conclusions

Our attempt to develop a single automated algorithm for generating gold standard classifications regarding the presence or absence of problem opioid use in a prevalent user cohort of patients receiving long-term ER/LA therapy was unsuccessful. The approach reported here may have utility for developing screening tools to identify patients for whom further clinical evaluation is warranted. Future work should focus on incident long-term opioid recipients (without distinguishing ER/LA from IR) and target subgroups of patients whose clinical course may be more homogeneous and, therefore, more likely to be reflected in structured EHR/claims data.

## Supplementary Material

Supplemental Material

## Transparency

### Declaration of financial/other interests

DSC, LAJ, AR, GS, MM, EJ, DJC, KH, SMS, and MVK are employees of Kaiser Permanente Washington. CAG, BH, and SLJ are employees of Kaiser Permanente Northwest. CAG has since retired. PMC and ADG were employees of Purdue Pharma, LP at the time the work was conducted and are currently employees of Johnson & Johnson and Indivior, Inc. respectively. CL and CLE are employees of Optum, Inc. AB is an employee of Amazon, CGG is an employee of Vanderbilt University, and JL is an employee of The Fred Hutchinson Cancer Research Center. Prior to conducting the work described here DSC, AR, DSC, KH, and MVK worked on projects funded by grants to Kaiser Permanente Health Research Institute for research on opioid risks funded by Pfizer, Inc. Peer reviewers on this manuscript have no relevant financial or other relationships to disclose.
