# Supplementary material for: Measuring problem prescription opioid use among patients receiving long-term opioid analgesic treatment: development and evaluation of an algorithm for use in EHR and claims data
Source: J Drug Assess. 2020 Apr 28;9(1):97–105. doi: 10.1080/21556660.2020.1750419 (PMC7241518; doi:10.1080/21556660.2020.1750419)
Supplement: Supplemental Material [file IJDA_A_1750419_SM8913.docx]

# Appendices

# Measuring problem prescription opioid use among patients receiving long-term opioid analgesic treatment: Development and evaluation of an algorithm for use in EHR and claims data

The following appendices are to be available as online supplements.

**Appendix A Fourteen ICD-9 diagnosis codes used in previous studies to
identify patients with problem opioid use** Page 2

**Appendix B Fifty-three predictors selected by LASSO modeling for inclusion in the
final problem opioid use classification model (selected from the set of
1,126 candidate predictors described in Appendix C)** Page 3

**Appendix C The 1,126 candidate predictors operationalized and considered for
inclusion in the model to classify patients with respect to presence or
absence of problem opioid use** Page 11

# Appendix A. Fourteen ICD-9 diagnosis codes used in previous studies to identify patients with problem opioid use

Diagnosis codes used in previous studies to identify patients with problem opioid use from the International Classification of Diseases, Ninth Revision (ICD-9). Such codes have typically been used in algorithms that consider the appearance of one or more of these codes in a patient’s medical record (or medical claims data) within a defined period of time to be evidence of problem opioid use. The absence of such codes is typically considered evidence that problem opioid use was not present. We refer to such algorithms as “simple algorithms” because of the relatively limited nature of the information from a patient’s record they consider.

| **ICD-9 diagnosis codes typically used in simple algorithms to identify patients with problem opioid, including codes for opioid abuse, dependence and poisoning.** | |
| --- | --- |
| **ICD-9 code** | **ICD-9 description** |
| 304 | Opioid Dependence, Unspecified |
| 304.01 | Opioid Dependence, Continuous |
| 304.02 | Opioid Dependence, Episodic |
| 304.03 | Opioid type dependence, in remission |
| 304.7 | Opioid/Other Dependence, Unspecified |
| 304.71 | Opioid/Other Dependence, Continuous |
| 304.72 | Opioid/Other Dependence, Episodic |
| 305.5 | Opioid Abuse, Unspecified |
| 305.51 | Opioid Abuse, Continuous |
| 305.52 | Opioid Abuse, Episodic |
| 305.53 | Opioid abuse, in remission |
| 965 | Poisoning by opium (alkaloids), unspecified |
| 965.02 | Poisoning by methadone |
| 965.09 | Poisoning by other opiates/narcotics |

# Appendix B. Fifty-three predictors selected by LASSO modeling for inclusion in the final problem opioid use classification model (selected from the set of 1,126 candidate predictors described in Appendix C)

The adaptive LASSO regression model considered a set of 1,126 candidate predictors (described in Appendix C) and selected 53 predictors for inclusion in the final problem opioid use classification algorithm. These 53 predictors are listed in the following table.

| **Appendix B. The 53 predictors selected by LASSO modeling for inclusion in the final problem opioid use classification algorithm** | | | |
| --- | --- | --- | --- |
| **Row** | **Predictor variable name** | **Predictor variable description** | **Coefficient** |
| 0 | Intercept | Intercept term | -- |
| 1 | AGE_GE65 | Yes/no is age as of Index Date >=65 | -0.36810 |
| 2 | Alcohol_ICD9_30390_YN | Ever diagnosed w/ ICD9 303.90 [Alcoholism, unspecified] | 0.92253 |
| 3 | ANTIANXIETY_TOTAL_QUARTERS_GE2 | Yes/no >=2 quarters with >=1 anti-anxiety medication dispensing during the quarter | 0.20399 |
| 4 | AnxietyDx_Ever_YN | Ever diagnosed with Anxiety disorder dx; ICD9: "300.0X","300.2X","300.3X","300.5X","308.3X" | 0.12369 |
| 5 | COUNT_BENZO_WITH_OPIOIDS_GE4 | Yes/no >=4 benzodiazepine dispensings AND >=1 opioid dispensing during a quarter | 0.31740 |
| 6 | COUNT_RXSUP_ANY_LE14_GE21 | Yes/no >=21 opioid dispensings (ER/LA or SA/IR) with 1-14 days’ supply during the observation period | 0.09824 |
| 7 | COUNT_RXSUP_ANY_LE14_QTR_GE7 | Yes/no >=7 opioid dispensings (ER/LA or SA/IR) with 1-14 days’ supply during a quarter | 0.09179 |
| 8 | COUNT_RXSUP_ER_8LE14_QTR_GE1 | Ever/never >=1 ER/LA opioid dispensing with 8-14 days’ supply during a quarter | 0.13671 |
| 9 | COUNT_RXSUP_ER_LE14_GE6 | Yes/no >=6 ER/LA opioid dispensing with 1-14 days’ supply during the observation period | 0.05084 |
| 10 | COUNT_RXSUP_ER_LE14_QTR_GE1 | Ever/never >=1 ER/LA opioid dispensing with 1-14 days’ supply during a quarter | 0.08956 |
| 11 | COUNT_RXSUP_ER_LE14_QTR_GE2 | Yes/no >=2 ER/LA opioid dispensing with 1-14 days’ supply during a quarter | -0.04353 |
| 12 | COUNT_RXSUP_ER_LE28_GE30 | Yes/no >=30 ER/LA dispensings with 1-28 days’ supply during the observation period | 0.12776 |
| 13 | DRUG_DEP_NOS_EVER | Ever diagnosed with specific non-opioid substance abuse/dependence codes 1) Drug dependence NOS (ICD9: 304.[90/91/92]) or 2) Non-dependent drug abuse (ICD9:305.[90/91/92]) | 0.27163 |
| 14 | EARLY_FILL_QTR_COUNT_NDC_GE4 | Yes/no >=4 dispensings with the same NDC code overlap by >=1 day during a quarter | 0.14214 |
| 15 | EARLY_FILL_QTR_DAYS_GE2_GE2 | Yes/no >=2 dispensings with the same NDC code overlap by >=2 days during a quarter | 0.33116 |
| 16 | EARLY_FILL_QTR_DAYS_SUM_GE16 | Yes/no >=16 total days of supply overlap for a single NDC code during a quarter | 0.48086 |
| 17 | ER_GE55_AVG_DAILY_MEQ_AGELE45 | Yes/no average daily MEQ >=55 during a quarter with >=1 ER encounter, and age <=45 | 0.67097 |
| 18 | ER_RX_COUNT_ANY_IN_QTR_GE2 | Yes/no >=2 opioids (ER/LA or SA/IR) dispensed on the same date as an ER encounter during a quarter | 0.44452 |
| 19 | ER_RX_COUNT_SA_GE2 | Yes/no >=2 SA/IR opioids dispensed on the same date as an ER encounter anytime during the observation period | 0.06633 |
| 20 | Ever_Alcohol_3039X | Ever diagnosed w/ Alcoholism (ICD9 303.[90/91/92]) | 0.52143 |
| 21 | Ever_SubAbuse_DX_PX_RX | Ever diagnosed with and/or receive treatment and/or medication related to Substance Abuse Non-Opioid Substance abuse diagnosis ICD9 included: Alcoholism: 303.XX, V11.3,303.X Specified Drug Dependence: 304.1X,304.2X,304.4X,304.5X,304.6X Cannabis drug dependence: 304.3X,305.2X Combination of drug dependence: 304.X8,304.9X Non-dependent Drug abuse: 305.3, 305.4X, 305.6", 305.7X, 305.8X, 305.9X Tobacco use disorder: 305.1X Use the AA treatment codes:'H0001', 'H0002', 'H0003', 'H0005', 'H0006', 'H0007', 'H0008', 'H0009', 'H0010', 'H0011', 'H0012', 'H0013', 'H0014', 'H0015', 'H0016', 'H0020', 'H0021', 'H0022', 'H0026', 'H0027', 'H0028', 'H0033', 'H0047', 'H2035', 'H2036', 'S9475', 'T1007', 'T1008', 'T1009', 'T1010', 'T1011', 'T1012', 'T1013'. The list of AA treatment medications (SAS file) is provided in Data Request package 12. | 0.36921 |
| 22 | FILL_OVERLAP_GE2_UDS_GE2_IN_QTR | Yes/no >=2 overlapping dispensings (ER/LA or SA/IR) and >=2 Urine Drug Screens in the same quarter | 0.05105 |
| 23 | GENDER | Gender (numeric, 0=male, 1=female) | -0.17353 |
| 24 | HeadtoOther_1mon_YN | Ever change in location of pain(headache/migraine to any other pain type (other than headache) such as Low back pain, Other back/neck disorder, Neuropathic pain, Fibromyalgia, Arthritis ) location within 30 days Low Back Pain: "721.3X", "722.10", "722.32", "722.52", "722.93", "724.02", "724.2XX", "724.3X", "724.5X" , "724.6X" , "724.7X" , "738.5X" , "739.3X" , "739.4", "846.0X", "846.1" , "846.2X" , "846.X3" , "846.8X" , "846.9X" , "847.2X" Other back Pain: "720.XX" , "721.0X" , "721.1X", "721.2X" , "721.4X", "721.5X" , "721.6X" , "721.7X", "721.8X", "721.9X" , "722.X0", "722.11", "722.2X", "722.30", "722.31", "722.39", "722.4", "722.51", "722.6", "722.7" , "722.8X", "722.90", "722.91", "722.92", "723.XX" , "724.01", "724.09", "724.1", "724.4X", "724.8X" , "724.9X" , "729.2X" , "737.0X", "737.1X" , "737.2X", "737.3X" , "737.9X", "738.4X" , "739.1" , "739.2", "741.0", "741.9" , "742.5X", "847.0X" , "847.1X", "847.3X" , "847.4X" , "847.9X" Headache/ Migraine: "339", "784.0", "346", "307.81" Neuropathic Pain : "053.10", "053.12", "053.13", "053.19", "053.2" , "053.7" , "053.9" , "250.6", "300.89", "333.84", "336.9X" , "337.0X" , "337.9X" , "346.20", "350" , "351.XX" , "352.XX" , "353.xx" , "354.XX" , "355.XX" , "356.XX" , "357.XX" , "377.30", "385.89", "388.50", "388.71", "511.0X" , "607.90", "608.89", "608.90", "625.90", "788.00", "788.10", "897.2" , "897.4" , "897.6X" , "950.XX" , "951.XX" , "951.XX" , "953.XX" , "954.XX" , "955.XX" , "956.XX" ,"957.XX" , "997.6X" Fibromyalgia: "729.1" Arthritis: "710.XX", "711.9X", "714.XX", "715.XX", "716.1X", "716.2X", "716.5X", "716.6X", "719.XX", "725.XX" , "726.XX", "727.XX" , "728.XX", "729.XX" | 0.30804 |
| 25 | i_A2_EVER_ER_GE120_SA_GE80_IN_Q | Interaction term: 1. Age 46-64, 2. Ever/never >=120 days’ supply of ER/LA opioids AND >=80 days’ supply of SA/IR opioids during a quarter | 0.24079 |
| 26 | I_AGEGE46LE64_Ever_Alcohol_3039X | Interaction term: 1. Age 46-64, 2. Ever diagnosed with alcohol dx codes: ICD9: 303.90, 303.91, 303.92, 303.93, V11.3 | 0.16301 |
| 27 | I_AGEGE46LE64xDRUGDEPNOSEVER | Interaction term: 1. Age 46-64 2. Ever diagnosed with specific non-opioid substance abuse/dependence codes (Drug dependence NOS (ICD9: 304.[90/91/92]) or Non-dependent drug abuse (ICD9:305.[90/91/92])) | 0.47706 |
| 28 | I_AGELE45_NDistQtrSubAbuseDxGE6 | Interaction term: 1. Age <=45 2. Ever >=6 quarters w/ 1+ visits related to Non-Opioid Substance Abuse (alcoholism, Specified drugs, Combination drugs, Cannabis, Tobacco dependence) ICD9 codes: Non-Opioid Substance abuse diagnosis ICD9 included: Alcoholism: 303.XX, V11.3,303.X Specified Drug Dependence: 304.1X,304.2X,304.4X,304.5X,304.6X Cannabis drug dependence: 304.3X,305.2X Combination of drug dependence: 304.X8,304.9X Non-dependent Drug abuse: 305.3, 305.4X,305.6",305.7X,305.8X,305.9X Tobacco use disorder: 305.1X | 0.05932 |
| 29 | MAX_EARLY_FILL_NDC_COUNT | Maximum number of overlapping dispensings for a single NDC code 6 months before Index Date and 12 months after | 0.03661 |
| 30 | MAX_EARLY_FILL_QTR_DAYS_PCT | Maximum percentage of dispensings that are early for a single NDC code during a quarter | 0.00517 |
| 31 | MAX_PCNT_RXSUP_ER_LE14_QTR | Maximum percentage of ER/LA dispensings during a quarter that have 1-14 days’ supply | 0.00400 |
| 32 | MAX_RXSUP_QTR_LITHIUM | Maximum days’ supply of Lithium dispensed during a quarter | 0.00063 |
| 33 | MaxVisitsAnyQtr_ComboDrugDepDx | Maximum visits in any quarter related to Combination of drug dependence dx ICD9: 304.8x, 305.9x. Variable value was top coded at 2. | 0.12490 |
| 34 | MaxVisitsAnyQtr_NDADx | Maximum visits in any quarter related to Nondependent Drug Abuse dx. ICD9: 305.3*-305.9*. Variable value was top coded at 2. | 0.67332 |
| 35 | N_DistinctQtrAnxietyDxGE5 | Number of quarters w/ 1 or more visits related to Anxiety disorder dx. ICD9: "300.0x","300.2x","300.3x","300.5x","308.3x". | 0.42363 |
| 36 | OPAbuse_30550_YN | Ever diagnosed w/ ICD9 305.50 [Opioid Abuse, Unspecified] | 0.52254 |
| 37 | OPDepDx_Ever_YN | Ever diagnosed with w/ Opioid Dependence. ICD9:"304.00", "304.01", "304.02", "304.03", "304.70", "304.71", "304.72". | 0.11629 |
| 38 | OVERLAP_SUP_LE14_SA_WKND_GE3 | Yes/no >=3 SA/IR opioids with 1-14 days’ supply dispensed on Saturday, Sunday, or Monday that overlaps by >=1 day with an adjacent SA/IR opioid dispensing (of any days’ supply) during a quarter | 0.19166 |
| 39 | OVERLAP_SUP_LE7_ER_WKND_GE1 | Ever/never >=1 ER/LA opioid with 1-7 days’ supply dispensed on Saturday, Sunday, or Monday that overlaps by >=1 day with an adjacent ER/LA opioid dispensing (of any days supply) during a quarter | 0.10129 |
| 40 | OVERLAP_SUP2_LE28_QTR_GE5 | Yes/no >=5 opioid dispensings (ER/LA or SA/IR) with 8-28 days’ supply overlap by 1-27 days* with an adjacent dispensing (also with 8-28 days’ supply) during a quarter (*in other words, these dispensings overlap by at least 1 day, but are not complete overlaps (i.e., not duplicate fills by opioid type, dispensing date, and days’ supply)) | 0.28472 |
| 41 | Pct_QtrMHDxGE17 | Ever had >=17% quarters w/ 1+ visits related to Mental Health diagnosis Depression:"296.2x","296.3x","296.82", "300.4x", "311" Bipolar Disorder:"296.0x","296.1x","296.4x","296.5x", "296.6x", "296.7x", "296.80" ,"296.81","296.89" Anxiety disorder:"300.0x","300.2x","300.3x","300.5x","308.x3" Other Mental Health disorders: "300.1", "300.5", "300.6", "300.7", "300.8", "300.9","307.4" ,"309.81" Other Mood disorder:"296.9x","300.4x", "308", "309.xx" Schizophernia/Schizoaffective:"295.1x","295.2x","295.3x","295.4x","295.5x","295.6x","295.7x","295.8x","295.9x" Other Psychotic disorders: "297.xx", "298.xx" Hepatitis or Cirrhosis: "573.3x","571.xx" | 0.19724 |
| 42 | Pct_QtrOPDepDxGE18 | Ever had >= 18% of quarters w/ visits related to Opioid Dependence dx ICD9:"304.00", "304.01", "304.02", "304.03", "304.70", "304.71", "304.72" | 0.31126 |
| 43 | Pct_QtrPOUDx | Percentage of quarters w/ 1+ visits related to Problem Opioid Dx (ICD9:304.XX-305.XX, 965.0X). Variable value was top coded at 8%. | 3.83026 |
| 44 | Pct_QtrSubAbuseDx | Percentage of quarters w/ 1+ visits related to Non-Opioid Substance Abuse diagnosis (alcoholism, Specified drugs, Combination drugs, Cannabis, Tobacco dependence). Variable value was top coded at 25%. | 0.17652 |
| 45 | Pct_QtrSubAbuseDxGE25 | Ever had >=25% quarters w/ 1+ visits related to Non-Opioid Substance Abuse (alcoholism, Specified drugs, Combination drugs, Cannabis, Tobacco dependence) Non-Opioid Substance abuse diagnosis ICD9 included: Alcoholism: 303.XX, V11.3,303.X Specified Drug Dependence: 304.1X,304.2X,304.4X,304.5X,304.6X Cannabis drug dependence: 304.3X,305.2X Combination of drug dependence: 304.X8,304.9X Non-dependent Drug abuse: 305.3, 305.4X,305.6",305.7X,305.8X,305.9X Tobacco use disorder: 305.1X | 0.20163 |
| 46 | POST_INDEX_RX_TREAT_AA_EVER | Ever/never >=1 dispensing for medication used to treat opioid abuse/addiction during 24 months after index date | 0.31312 |
| 47 | POUDx_Ever_YN | Ever diagnosed with w/ Problem Opioid Dependence, Abuse, Poisoning, Heroin Dependence:"304.00", "304.01", "304.02", "304.03", "304.70", "304.71", "304.72" Abuse:"305.50","305.51","305.52","305.53" Poisoning (excluding heroin):"965.00","965.02","965.09" Heroin:"965.01" | 0.27975 |
| 48 | PX_80100 | Ever/never >=1 CPT code for Urine Drug Screen, qualitative multiple drug class (CPT code 80100) | 0.41900 |
| 49 | PX_80102 | Ever/never >=1 CPT code for Urine Drug Screen confirmation (CPT code 80102) | 0.10697 |
| 50 | UDS_EVER_TOTAL_QUARTERS | Total number of quarters with >=1 Urine Drug Screening (CPT codes: '80100','80101','80102','80154','80299','80300','83925','99408','99409') during the quarter | 0.01221 |
| 51 | UDS_EVER_TOTAL_QUARTERS_GE2 | Yes/no >=2 quarters with at least one Urine Drug Screening (CPT codes: '80100','80101','80102','80154','80299','80300','83925','99408','99409') during the quarter | 0.07485 |
| 52 | UDS_EVER_TOTAL_QUARTERS_LE2010 | Total number of quarters with at least one Urine Drug Screen (CPT codes: '80100','80101','80102','80154','80299','80300','83925','99408','99409') during non-routine screening (at Group Health, this is 2010 and earlier) | 0.10002 |
| 53 | UDS_TOTAL_COUNT_LE2010 | Total number of Urine Drug Screens (CPT codes: '80100','80101','80102','80154','80299','80300','83925','99408','99409') during non-routine screening (at Group Health, this is 2010 and earlier) | 0.04695 |

# Appendix C. The 1,126 candidate predictors operationalized and considered for inclusion in the model to classify patients with respect to presence or absence of problem opioid use

As described in the accompanying manuscript, we operationalized 1,126 candidate predictors of problem prescription opioid use based on EHR/claims data. a subset of 53 predictors from these 1,126 candidates were selected for inclusion in the final classification model using adaptive LASSO regression.

| **Appendix C. The 1,126 candidate predictor variables considered for inclusion in the classification algorithm for problem opioid use.** | | |
| --- | --- | --- |
| **Row** | **Potential predictor variable name** | **Potential predictor variable description** |
| 1 | AGE_LE45 | AGE_LE45 |
| 2 | AGE_GROUP | AGE_GROUP |
| 3 | AGE_GE46_LE64 | AGE_GE46_LE64 |
| 4 | AGE_GE65 | AGE_GE65 |
| 5 | N_DistinctQtrPOUDx | [N_DistinctQtrPOUDx]#Distinct quarters w/ Problem Opioid Dx (ICD9:304.*-305.*) |
| 6 | N_DistinctQtrOPDepDx | [N_DistinctQtrOPDepDx]#Distinct quarters w/ Opioid Dependence dx (ICD9:304.* only) |
| 7 | N_DistinctQtrOPAbuseDx | [N_DistinctQtrOPAbuseDx]#Distinct quarters w/ Opioid Abuse dx (ICD9:305.* only) |
| 8 | N_DistinctQtrSubAbuseDx | [N_DistinctQtrSubAbuseDx]#Distinct quarters w/ Non-Opioid Substance Abuse (alcoholism, Specified drugs, Combination drugs,Cannibis, Tobacco dependence) |
| 9 | N_DistinctQtrAlcoholDx | [N_DistinctQtrAlcoholDx]#Distinct quarters w/ Alcohol Abuse dx(ICD9: 303.0*, 303.9*, V11.3) |
| 10 | N_DistinctQtrSpecDrugDepDx | [N_DistinctQtrSpecDrugDepDx]#Distinct quarters w/ Specified Drug Dependence dx(ICD9: 304.*) |
| 11 | N_DistinctQtrCanDepDx | [N_DistinctQtrCanDepDx]#Distinct quarters w/ Cannabis Dependence dx(ICD9: 304.3*, 305.2*) |
| 12 | N_DistinctQtrComboDrugDepDx | [N_DistinctQtrComboDrugDepDx]#Distinct quarters w/ Combination of drug dependence dx(ICD9: 304.8*, 305.9*) |
| 13 | N_DistinctQtrNDADx | [N_DistinctQtrNDADx]#Distinct quarters w/ Nondependent Drug Abuse dx(ICD9: 305.3*-305.9*) |
| 14 | N_DistinctQtrTobbacoDx | [N_DistinctQtrTobbacoDx]#Distinct quarters w/ Tobacco disorder dx(ICD9: 305.1*) |
| 15 | N_DistinctQtrMHDx | 66666 |
| 16 | N_DistinctQtrDepressDx | [N_DistinctQtrDepressDx]#Distinct quarters w/ Depression dx |
| 17 | N_DistinctQtrBipolarDx | [N_DistinctQtrBipolarDx]#Distinct quarters w/ Bipolar dx |
| 18 | N_DistinctQtrAnxietyDx | [N_DistinctQtrAnxietyDx]#Distinct quarters w/ Anxietry disorder dx |
| 19 | N_DistinctQtrOtherMHDx | [N_DistinctQtrOtherMHDx]#Distinct quarters w/ Other Mental Health disorder dx |
| 20 | N_DistinctQtrOtherMoodDx | [N_DistinctQtrOtherMoodDx]#Distinct quarters w/ Other Mood disorder dx |
| 21 | N_DistinctQtrOtherPsyDx | [N_DistinctQtrOtherPsyDx]#Distinct quarters w/ Other Psychotic disorder dx |
| 22 | N_DistinctQtrHepaCirrDx | [N_DistinctQtrHepaCirrDx]#Distinct quarters w/ Hepatitis or Cirrhosis disorder dx |
| 23 | N_DistinctQtrPainDx | [N_DistinctQtrPainDx]#Distinct quarters w/ Pain dx |
| 24 | N_DistinctQtrLowBackPainDx | [N_DistinctQtrLowBackPainDx]#Distinct quarters w/ Low Back Pain dx |
| 25 | N_DistinctQtrOthBackPainDx | [N_DistinctQtrOthBackPainDx]#Distinct quarters w/ Other Back Pain dx |
| 26 | N_DistinctQtrHeadAcheDx | [N_DistinctQtrHeadacheDx]#Distinct quarters w/ Headache or Migrane dx |
| 27 | N_DistinctQtrFibroDx | [N_DistinctQtrFibroDx]#Distinct quarters w/ Fibromyalgia dx |
| 28 | N_DistinctQtrArthDx | [N_DistinctQtrArthDx]#Distinct quarters w/ Arthritis dx |
| 29 | Pct_QtrPOUDx | [Pct_QtrPOUDx]%Qtrs w/ Problem Opioid Dx (ICD9:304.*-305.*) |
| 30 | Pct_QtrOPDepDx | [Pct_QtrOPDepDx]%Qtrs w/ Opioid Dependence dx (ICD9:304.* only) |
| 31 | Pct_QtrOPAbuseDx | [Pct_QtrOPAbuseDx]%Qtrs w/ Opioid Abuse dx (ICD9:305.* only) |
| 32 | Pct_QtrSubAbuseDx | [Pct_QtrSubAbuseDx]%Qtrs w/ Non-Opioid Substance Abuse (alcoholism, Specified drugs, Combination drugs,Cannibis, Tobacco dependence) |
| 33 | Pct_QtrAlcoholDx | [Pct_QtrAlcoholDx]%Qtrs w/ Alcohol Abuse dx(ICD9: 303.0*, 303.9*, V11.3) |
| 34 | Pct_QtrSpecDrugDepDx | [Pct_QtrSpecDrugDepDx]%Qtrs w/ Specified Drug Dependence dx(ICD9: 304.*) |
| 35 | Pct_QtrComboDrugDepDx | [Pct_QtrComboDrugDepDx]%Qtrs w/ Combination of drug dependence dx(ICD9: 304.8*, 305.9*) |
| 36 | Pct_QtrNDADx | [Pct_QtrNDADx]%Qtrs w/ Nondependent Drug Abuse dx(ICD9: 305.3*-305.9*) |
| 37 | Pct_QtrTobbacoDx | [Pct_QtrTobbacoDx]%Qtrs w/ Tobacco disorder dx(ICD9: 305.1*) |
| 38 | Pct_QtrMHDx | [Pct_QtrMHDx]%Qtrs w/ Mental Health dx(ICD9: 305.1*) |
| 39 | Pct_QtrDepressDx | [Pct_QtrDepressDx]%Qtrs w/ Depression dx |
| 40 | Pct_QtrBipolarDx | [Pct_QtrBipolarDx]%Qtrs w/ Bipolar dx |
| 41 | Pct_QtrAnxietyDx | [Pct_QtrAnxietyDx]%Qtrs w/ Anxietry disorder dx |
| 42 | Pct_QtrOtherMHDx | [Pct_QtrOtherMHDx]%Qtrs w/ Other Mental Health disorder dx |
| 43 | Pct_QtrOtherMoodDx | [Pct_QtrOtherMoodDx]%Qtrs w/ Other Mood disorder dx |
| 44 | Pct_QtrHepaCirrDx | [Pct_QtrHepaCirrDx]%Qtrs w/ Hepatitis or Cirrhosis disorder dx |
| 45 | Pct_QtrPainDx | [Pct_QtrPainDx]%Qtrs w/ Pain dx |
| 46 | Pct_QtrLowBackPainDx | [Pct_QtrLowBackPainDx]%Qtrs w/ Low Back Pain dx |
| 47 | Pct_QtrOthBackPainDx | [Pct_QtrOthBackPainDx]%Qtrs w/ Other Back Pain dx |
| 48 | Pct_QtrHeadAcheDx | [Pct_QtrHeadacheDx]%Qtrs w/ Headache or Migrane dx |
| 49 | Pct_QtrNeuroPainDx | [Pct_QtrNeuroPainDx]%Qtrs w/ Neuropathic Pain dx |
| 50 | Pct_QtrFibroDx | [Pct_QtrFibroDx]%Qtrs w/ Fibromialgia dx |
| 51 | Pct_QtrArthDx | [Pct_QtrArthDx]%Qtrs w/ Arthritis dx |
| 52 | POUDx_Ever_YN | [POUDx_Ever_YN]Ever w/ Problem Opioid Dx (ICD9:304.*-305.*) |
| 53 | OPDepDx_Ever_YN | [OPDepDx_Ever_YN]Ever w/ Opioid Dependence dx (ICD9:304.* only) |
| 54 | OPAbuseDx_Ever_YN | [OPAbuseDx_Ever_YN]Ever w/ Opioid Abuse dx (ICD9:305.* only) |
| 55 | PoisonDx_Ever_YN | [PoisonDx_Ever_YN]Ever w/ Poisoning dx (ICD9:965.0* only) |
| 56 | SubAbuseDx_Ever_YN | [SubAbuseDx_Ever_YN]Ever w/ Non-Opioid Substance Abuse (alcoholism, Specified drugs, Combination drugs,Cannibis, Tobacco dependence) |
| 57 | AlcoholDx_Ever_YN | [AlcoholDx_Ever_YN]Ever w/ Alcohol Abuse dx(ICD9: 303.0*, 303.9*, V11.3) |
| 58 | SpecDrugDepDx_Ever_YN | [SpecDrugDepDx_Ever_YN]Ever w/ Specified Drug Dependence dx(ICD9: 304.*) |
| 59 | CanDepDx_Ever_YN | [CanDepDx_Ever_YN]Ever w/ Cannabis Dependence dx(ICD9: 304.3*, 305.2*) |
| 60 | ComboDrugDepDx_Ever_YN | [ComboDrugDepDx_Ever_YN]Ever w/ Combination of drug dependence dx(ICD9: 304.8*, 305.9*) |
| 61 | NDADx_Ever_YN | [NDADx_Ever_YN]Ever w/ Nondependent Drug Abuse dx(ICD9: 305.3*-305.9*) |
| 62 | TobbacoDx_Ever_YN | [TobbacoDx_Ever_YN]Ever w/ Tobacco disorder dx(ICD9: 305.1*) |
| 63 | MHDx_Ever_YN | [MHDx_Ever_YN]Ever w/ Mental Health dx(ICD9: 305.1*) |
| 64 | DepressDx_Ever_YN | [DepressDx_Ever_YN]Ever w/ Depression dx |
| 65 | BipolarDx_Ever_YN | [BipolarDx_Ever_YN]Ever w/ Bipolar dx |
| 66 | AnxietyDx_Ever_YN | [AnxietyDx_Ever_YN]Ever w/ Anxietry disorder dx |
| 67 | OtherMHDx_Ever_YN | [OtherMHDx_Ever_YN]Ever w/ Other Mental Health disorder dx |
| 68 | OtherMoodDx_Ever_YN | [OtherMoodDx_Ever_YN]Ever w/ Other Mood disorder dx |
| 69 | SchizoDx_Ever_YN | [SchizoDx_Ever_YN]Ever w/ Schizophernia/Schizoaffective disorder dx |
| 70 | OtherPsyDx_Ever_YN | [OtherPsyDx_Ever_YN]Ever w/ Other Psychotic disorder dx |
| 71 | HepaCirrDx_Ever_YN | [HepaCirrDx_Ever_YN]Ever w/ Hepatitis or Cirrhosis disorder dx |
| 72 | PainDx_Ever_YN | [PainDx_Ever_YN]Ever w/ Pain dx |
| 73 | LowBackPainDx_Ever_YN | [LowBackPainDx_Ever_YN]Ever w/ Low Back Pain dx |
| 74 | OthBackPainDx_Ever_YN | [OthBackPainDx_Ever_YN]Ever w/ Other Back Pain dx |
| 75 | HeadAcheDx_Ever_YN | [HeadacheDx_Ever_YN]Ever w/ Headache or Migrane dx |
| 76 | NeuroPainDx_Ever_YN | [NeuroPainDx_Ever_YN]Ever w/ Neuropathic Pain dx |
| 77 | ArthDx_Ever_YN | [ArthDx_Ever_YN]Ever w/ Arthritis dx |
| 78 | MaxVisitsAnyQtr_POUDx | [MaxVisitsAnyQtr_POUDx]Max #visits in any qtr w/ Problem Opioid Dx (ICD9:304.*-305.*) |
| 79 | MaxVisitsAnyQtr_OPDepDx | [MaxVisitsAnyQtr_OPDepDx]Max #visits in any qtr w/ Opioid Dependence dx (ICD9:304.* only) |
| 80 | MaxVisitsAnyQtr_OPAbuseDx | [MaxVisitsAnyQtr_OPAbuseDx]Max #visits in any qtr w/ Opioid Abuse dx (ICD9:305.* only) |
| 81 | MaxVisitsAnyQtr_SubAbuseDx | [MaxVisitsAnyQtr_SubAbuseDx]Max #visits in any qtr w/ Non-Opioid Substance Abuse (alcoholism, Specified drugs, Combination drugs,Cannibis, Tobacco dependence) |
| 82 | MaxVisitsAnyQtr_AlcoholDx | [MaxVisitsAnyQtr_AlcoholDx]Max #visits in any qtr w/ Alcohol Abuse dx(ICD9: 303.0*, 303.9*, V11.3) |
| 83 | MaxVisitsAnyQtr_SpecDrugDepDx | [MaxVisitsAnyQtr_SpecDrugDepDx]Max #visits in any qtr w/ Specified Drug Dependence dx(ICD9: 304.*) |
| 84 | MaxVisitsAnyQtr_CanDepDx | [MaxVisitsAnyQtr_CanDepDx]Max #visits in any qtr w/ Cannibis Dependence dx(ICD9: 304.3*, 305.2*) |
| 85 | MaxVisitsAnyQtr_ComboDrugDepDx | [MaxVisitsAnyQtr_ComboDrugDepDx]Max #visits in any qtr w/ Combination of drug dependence dx(ICD9: 304.8*, 305.9*) |
| 86 | MaxVisitsAnyQtr_NDADx | [MaxVisitsAnyQtr_NDADx]Max #visits in any qtr w/ Nondependent Drug Abuse dx(ICD9: 305.3*-305.9*) |
| 87 | MaxVisitsAnyQtr_TobbacoDx | [MaxVisitsAnyQtr_TobbacoDx]Max #visits in any qtr w/ Tobacco disorder dx(ICD9: 305.1*) |
| 88 | MaxVisitsAnyQtr_MHDx | [MaxVisitsAnyQtr_MHDx]Max #visits in any qtr w/ Mental Health dx(ICD9: 305.1*) |
| 89 | MaxVisitsAnyQtr_DepressDx | [MaxVisitsAnyQtr_DepressDx]Max #visits in any qtr w/ Depression dx |
| 90 | MaxVisitsAnyQtr_BipolarDx | [MaxVisitsAnyQtr_BipolarDx]Max #visits in any qtr w/ Bipolar dx |
| 91 | MaxVisitsAnyQtr_AnxietyDx | [MaxVisitsAnyQtr_AnxietyDx]Max #visits in any qtr w/ Anxietry disorder dx |
| 92 | MaxVisitsAnyQtr_OtherMHDx | [MaxVisitsAnyQtr_OtherMHDx]Max #visits in any qtr w/ Other Mental Health disorder dx |
| 93 | MaxVisitsAnyQtr_OtherMoodDx | [MaxVisitsAnyQtr_OtherMoodDx]Max #visits in any qtr w/ Other Mood disorder dx |
| 94 | MaxVisitsAnyQtr_SchizoDx | [MaxVisitsAnyQtr_SchizoDx]Max #visits in any qtr w/ Schizophernia/Schizoaffective disorder dx |
| 95 | MaxVisitsAnyQtr_OtherPsyDx | [MaxVisitsAnyQtr_OtherPsyDx]Max #visits in any qtr w/ Other Psychotic disorder dx |
| 96 | MaxVisitsAnyQtr_HepaCirrDx | [MaxVisitsAnyQtr_HepaCirrDx]Max #visits in any qtr w/ Hepatitis or Cirrhosis disorder dx |
| 97 | MaxVisitsAnyQtr_PainDx | [MaxVisitsAnyQtr_PainDx]Max #visits in any qtr w/ Pain dx |
| 98 | MaxVisitsAnyQtr_LowBackPainDx | [MaxVisitsAnyQtr_LowBackPainDx]Max #visits in any qtr w/ Low Back Pain dx |
| 99 | MaxVisitsAnyQtr_OthBackPainDx | [MaxVisitsAnyQtr_OthBackPainDx]Max #visits in any qtr w/ Other Back Pain dx |
| 100 | MaxVisitsAnyQtr_HeadAcheDx | [MaxVisitsAnyQtr_HeadacheDx]Max #visits in any qtr w/ Headache or Migrane dx |
| 101 | MaxVisitsAnyQtr_NeuroPainDx | [MaxVisitsAnyQtr_NeuroPainDx]Max #visits in any qtr w/ Neuropathic Pain dx |
| 102 | MaxVisitsAnyQtr_FibroDx | [MaxVisitsAnyQtr_FibroDx]Max #visits in any qtr w/ Fibromialgia dx |
| 103 | MaxVisitsAnyQtr_ArthDx | [MaxVisitsAnyQtr_ArthDx]Max #visits in any qtr w/ Arthritis dx |
| 104 | OPDep_30400_YN | [OPDep_30400_YN]Ever w/ ICD9 304.00 [Opioid Dependence, Unspecified] |
| 105 | OPDep_30401_YN | [OPDep_30401_YN]Ever w/ ICD9 304.01 [Opioid Dependence, Continuous] |
| 106 | OPDep_30402_YN | [OPDep_30402_YN]Ever w/ ICD9 304.02 [Opioid Dependence, Episodic] |
| 107 | OPDep_30403_YN | [OPDep_30403_YN]Ever w/ ICD9 304.03 [Opioid Dependence, in remission] |
| 108 | OPDep_30470_YN | [OPDep_30470_YN]Ever w/ ICD9 304.70 [Opioid/Other Dependence, Unspecified] |
| 109 | OPDep_30471_YN | [OPDep_30471_YN]Ever w/ ICD9 304.71 [Opioid/Other Dependence, Continuous] |
| 110 | OPAbuse_30550_YN | [OPAbuse_30550_YN]Ever w/ ICD9 305.50 [Opioid Abuse, Unspecified] |
| 111 | OPAbuse_30551_YN | [OPAbuse_30551_YN]Ever w/ ICD9 305.51 [Opioid Abuse, Continuous] |
| 112 | OPAbuse_30553_YN | [OPAbuse_30553_YN]Ever w/ ICD9 305.53 [Opioid Abuse, in remission] |
| 113 | Poisoning_96500_YN | [Poisoning_96500_YN]Ever w/ ICD9 965.00 [Poisoning by opium (alkaloids), unspecified] |
| 114 | Poisoning_96502_YN | [Poisoning_96502_YN]Ever w/ ICD9 965.02 [Poisoning by methadone] |
| 115 | Poisoning_96509_YN | [Poisoning_96509_YN]Ever w/ ICD9 965.09 [Poisoning by other opiates/narcotics] |
| 116 | Alcohol_ICD9_30300_YN | [Alcohol_ICD9_30300_YN]Ever w/ ICD9 303.00 [Alcoholism, unspecified] |
| 117 | Alcohol_ICD9_30301_YN | [Alcohol_ICD9_30301_YN]Ever w/ ICD9 303.01 [Alcoholism, continuous] |
| 118 | Alcohol_ICD9_30390_YN | [Alcohol_ICD9_30390_YN]Ever w/ ICD9 303.90 [Alcoholism, unspecified] |
| 119 | Alcohol_ICD9_30391_YN | [Alcohol_ICD9_30391_YN]Ever w/ ICD9 303.91 [Alcoholism, continuous] |
| 120 | Alcohol_ICD9_30393_YN | [Alcohol_ICD9_30393_YN]Ever w/ ICD9 303.93 [Alcoholism, in remission] |
| 121 | Alcohol_ICD9_V113_YN | [Alcohol_ICD9_V113_YN]Ever w/ ICD9 V11.3 [Personal History of Alcoholism] |
| 122 | SDD_ICD9_30410_YN | [SDD_ICD9_30410_YN]Ever w/ ICD9 304.10 [Specified drug dependence-Sedative, hypnotic or anxiolytic dependence, unspecified] |
| 123 | SDD_ICD9_30411_YN | [SDD_ICD9_30411_YN]Ever w/ ICD9 304.11 [Specified drug dependence-Sedative, hypnotic or anxiolytic dependence, continuous] |
| 124 | SDD_ICD9_30420_YN | [SDD_ICD9_30420_YN]Ever w/ ICD9 304.20 [Cocaine dependence, unspecified convert] |
| 125 | SDD_ICD9_30421_YN | [SDD_ICD9_30421_YN]Ever w/ ICD9 304.21 [Cocaine dependence, continuous convert] |
| 126 | SDD_ICD9_30423_YN | [SDD_ICD9_30423_YN]Ever w/ ICD9 304.23 [Cocaine dependence, in remission convert] |
| 127 | SDD_ICD9_30440_YN | [SDD_ICD9_30440_YN]Ever w/ ICD9 304.40 [Amphetamine and other psychostimulant dependence, unspecified] |
| 128 | SDD_ICD9_30442_YN | [SDD_ICD9_30442_YN]Ever w/ ICD9 304.42 [Amphetamine and other psychostimulant dependence, episodic] |
| 129 | SDD_ICD9_30443_YN | [SDD_ICD9_30443_YN]Ever w/ ICD9 304.43 [Amphetamine and other psychostimulant dependence, in remission] |
| 130 | SDD_ICD9_30450_YN | [SDD_ICD9_30450_YN]Ever w/ ICD9 304.50 [Hallucinogen dependence, unspecified] |
| 131 | SDD_ICD9_30451_YN | [SDD_ICD9_30451_YN]Ever w/ ICD9 304.51 [Hallucinogen dependence, continuous] |
| 132 | SDD_ICD9_30452_YN | [SDD_ICD9_30452_YN]Ever w/ ICD9 304.52 [Hallucinogen dependence, episodic] |
| 133 | SDD_ICD9_30453_YN | [SDD_ICD9_30453_YN]Ever w/ ICD9 304.53 [Hallucinogen dependence, in remission] |
| 134 | SDD_ICD9_30460_YN | [SDD_ICD9_30460_YN]Ever w/ ICD9 304.60 [Other specified drug dependence, unspecified] |
| 135 | SDD_ICD9_30461_YN | [SDD_ICD9_30461_YN]Ever w/ ICD9 304.61 [Other specified drug dependence, continuous] |
| 136 | Cannibis_ICD9_30430_YN | [Cannibis_ICD9_30430_YN]Ever w/ ICD9 304.30 [Cannabis dependence, unspecified] |
| 137 | Cannibis_ICD9_30431_YN | [Cannibis_ICD9_30431_YN]Ever w/ ICD9 304.31 [Cannabis dependence, continuous] |
| 138 | Cannibis_ICD9_30432_YN | [Cannibis_ICD9_30432_YN]Ever w/ ICD9 304.32 [Cannabis dependence, episodic] |
| 139 | Cannibis_ICD9_30433_YN | [Cannibis_ICD9_30433_YN]Ever w/ ICD9 304.33 [Cannabis dependence, in remission convert] |
| 140 | Cannibis_ICD9_30420_YN | [Cannibis_ICD9_30420_YN]Ever w/ ICD9 304.20 [Cannabis dependence, unspecified convert] |
| 141 | Cannibis_ICD9_30421_YN | [Cannibis_ICD9_30421_YN]Ever w/ ICD9 304.21 [Cannabis dependence, continuous convert] |
| 142 | ComboDrug_ICD9_30480_YN | [ComboDrug_ICD9_30480_YN]Ever w/ ICD9 304.80 [Combinations of drug dependence, unspecified] |
| 143 | ComboDrug_ICD9_30483_YN | [ComboDrug_ICD9_30483_YN]Ever w/ ICD9 304.83 [Combinations of drug dependence, in remission] |
| 144 | ComboDrug_ICD9_30490_YN | [ComboDrug_ICD9_30490_YN]Ever w/ ICD9 304.90 [Combinations of drug dependence, unspecified] |
| 145 | ComboDrug_ICD9_30491_YN | [ComboDrug_ICD9_30491_YN]Ever w/ ICD9 304.91 [Combinations of drug dependence, continuous] |
| 146 | ComboDrug_ICD9_30493_YN | [ComboDrug_ICD9_30493_YN]Ever w/ ICD9 304.93 [Combinations of drug dependence, in remission] |
| 147 | NondepDrugAbuse_ICD9_30540_YN | [NondepDrugAbuse_ICD9_30540_YN]Ever w/ ICD9 305.40 [Nondependent sedative, hypnotic or anxiolytic abuse, unspecified] |
| 148 | NondepDrugAbuse_ICD9_30560_YN | [NondepDrugAbuse_ICD9_30560_YN]Ever w/ ICD9 305.60 [Nondependent cocaine abuse, unspecified] |
| 149 | NondepDrugAbuse_ICD9_30573_YN | [NondepDrugAbuse_ICD9_30573_YN]Ever w/ ICD9 305.73 [Nondependent amphetamine or related acting sympathomimetic abuse, in remission] |
| 150 | NondepDrugAbuse_ICD9_30590_YN | [NondepDrugAbuse_ICD9_30590_YN]Ever w/ ICD9 305.90 [Nondependent other mixed or unspecified drug abuse, unspecified] |
| 151 | NondepDrugAbuse_ICD9_30593_YN | [NondepDrugAbuse_ICD9_30593_YN]Ever w/ ICD9 305.93 [Nondependent other mixed or unspecified drug abuse, in remission] |
| 152 | Tobacco_ICD9_30510_YN | [Tobacco_ICD9_30510_YN]Ever w/ ICD9 305.10 [Tobacco use disorder] |
| 153 | SED_HYP_ANX_EVER | [SED_HYP_ANX_EVER]Presence, ever, of dx codes for sedative, hypnotic or anxiolytic abuse dependence (304.[10/11/12]) or abuse (305.[40/41/43]) |
| 154 | COCAINE_DEP_AB_EVER | [COCAINE_DEP_AB_EVER]Presence, ever, of dx codes for Cocaine dependence (304.20) or abuse (305.[60/61]) |
| 155 | CANNIB_DEP_AB_EVER | [CANNIB_DEP_AB_EVER]Presence, ever, of dx codes for Cannabis dependence (304.[30/32/33] ) or abuse (305.[20/23]) |
| 156 | AMPHET_DEP_AB_EVER | [AMPHET_DEP_AB_EVER]Presence, ever, of dx codes for Amphetamine dependence (304.[40/41/43]) or abuse (305.70) |
| 157 | OPI_OTHER_DEP_EVER | [OPI_OTHER_DEP_EVER]Presence, ever, of dx codes for Opioid/other dependence (304.[70/71/72]) |
| 158 | COMBO_DRUG_DEP_EVER | [COMBO_DRUG_DEP_EVER]Presence, ever, of dx codes for Combination drug dependence (304.[80/81/82]) |
| 159 | DRUG_DEP_NOS_EVER | [DRUG_DEP_NOS_EVER]Presence, ever, of dx codes for Drug dependence NOS (304.[90/91/92]) or drug abuse NEC (305.[90/91/92]) |
| 160 | OPI_AB_EVER | [OPI_AB_EVER]Presence, ever, of dx codes for Opioid abuse (305.[50/51/52/53] |
| 161 | OPI_DEP_EVER | [OPI_DEP_EVER]Presence, ever, of dx codes for Opioid dependence, episodic (304.02), opioid dependence, in remission(304.03) |
| 162 | HYBRID_DRUG_DEP_AB_EVER | [HYBRID_DRUG_DEP_AB_EVER]Presence, ever, of multiple DX codes304.[10/11/12], 305.[40/41/43], 304.51, 304.20, 305.[60/61], 304.[40/41/43], 304.[70/71/72], 304.[80/81/82], 304.02, and 304.03 |
| 163 | PTSD_Ever_YN | [PTSD_Ever_YN] Ever w/ Post-traumatic stress disorder(PTSD)ICD9 dx 309.81(1=Yes, 0=No) |
| 164 | Bupe_Disp_3days_of_OpDepDx_YN | [Bupe_Disp_3days_of_OpDepDx_YN] Ever w/ Buprenorphine dispense within 3 days of Opioid Dependence DX visit (1=Yes, 0=No) |
| 165 | Bupe_Disp_7days_of_OpDepDx_YN | [Bupe_Disp_7days_of_OpDepDx_YN] Ever w/ Buprenorphine dispense within 7 days of Opioid Dependence DX visit (1=Yes, 0=No) |
| 166 | Bupe_Disp_10days_of_OpDepDx_YN | [Bupe_Disp_10days_of_OpDepDx_YN] Ever w/ Buprenorphine dispense within 10 days of Opioid Dependence DX visit (1=Yes, 0=No) |
| 167 | ChangePainLoc_1wk_YN | [ChangePainLoc_1wk_YN] Ever change in the location of pain within 7 days (1=Yes, 0=No) |
| 168 | LowbacktoOther_1wk_YN | [LowbacktoOther_1wk_YN] Ever change in the location of pain(low back to any other pain type) within 7 days (1=Yes, 0=No) |
| 169 | BackNecktoOther_1wk_YN | [BackNecktoOther_1wk_YN] Ever change in the location of pain(Back/neck to any other pain type) within 7 days (1=Yes, 0=No) |
| 170 | HeadtoOther_1wk_YN | [HeadtoOther_1wk_YN] Ever change in location of pain(headache/migraine to any other pain type) location within 7 days (1=Yes, 0=No) |
| 171 | NeurotoOther_1wk_YN | [NeurotoOther_1wk_YN] Ever change in location of pain(neuropathic pain to any other pain type) within 7 days (1=Yes, 0=No) |
| 172 | ArthtoOther_1wk_YN | [ArthtoOther_1wk_YN] Ever change in location of pain (arthritis to any other pain type) within 7 days (1=Yes, 0=No) |
| 173 | ChangePainLoc_1mon_YN | [ChangePainLoc_1mon_YN] Ever change in the location of pain within 30 days (1=Yes, 0=No) |
| 174 | LowbacktoOther_1mon_YN | [LowbacktoOther_1mon_YN] Ever change in the location of pain(low back to any other pain type) within 30 days (1=Yes, 0=No) |
| 175 | BackNecktoOther_1mon_YN | [BackNecktoOther_1mon_YN] Ever change in the location of pain(Back/neck to any other pain type) within 30 days (1=Yes, 0=No) |
| 176 | HeadtoOther_1mon_YN | [HeadtoOther_1mon_YN] Ever change in location of pain(headache/migraine to any other pain type) location within 30 days (1=Yes, 0=No) |
| 177 | NeurotoOther_1mon_YN | [NeurotoOther_1mon_YN] Ever change in location of pain(neuropathic pain to any other pain type) within 30 days (1=Yes, 0=No) |
| 178 | ArthtoOther_1mon_YN | [ArthtoOther_1mon_YN] Ever change in location of pain (arthritis to any other pain type) within 30 days (1=Yes, 0=No) |
| 179 | ChangePainLoc_1yr_YN | [ChangePainLoc_1yr_YN] Ever change in the location of pain within 365 days (1=Yes, 0=No) |
| 180 | LowbacktoOther_1yr_YN | [LowbacktoOther_1yr_YN] Ever change in the location of pain(low back to any other pain type) within 365 days (1=Yes, 0=No) |
| 181 | BackNecktoOther_1yr_YN | [BackNecktoOther_1yr_YN] Ever change in the location of pain(Back/neck to any other pain type) within 365 days (1=Yes, 0=No) |
| 182 | HeadtoOther_1yr_YN | [HeadtoOther_1yr_YN] Ever change in location of pain(headache/migraine to any other pain type) location within 365 days (1=Yes, 0=No) |
| 183 | NeurotoOther_1yr_YN | [NeurotoOther_1yr_YN] Ever change in location of pain(neuropathic pain to any other pain type) within 365 days (1=Yes, 0=No) |
| 184 | ArthtoOther_1yr_YN | [ArthtoOther_1yr_YN] Ever change in location of pain (arthritis to any other pain type) within 365 days (1=Yes, 0=No) |
| 185 | No_of_Distinct_Pain_Loc |  |
| 186 | Ever_NoofDistinctPainLoc_GE3 | [Ever_NoofDistinctPainLoc_GE3] Ever had >= 3 distinct pain location visits (1=Yes, 0=No) |
| 187 | Ever_NoofDistinctPainLoc_GE4 | [Ever_NoofDistinctPainLoc_GE4] Ever had >= 4 distinct pain location visits (1=Yes, 0=No) |
| 188 | Ever_NoofDistinctPainLoc_GE5 | [Ever_NoofDistinctPainLoc_GE5] Ever had >= 5 distinct pain location visits (1=Yes, 0=No) |
| 189 | Ever_NoofDistinctPainLoc_GE6 | [Ever_NoofDistinctPainLoc_GE6] Ever had >= 6 distinct pain location visits (1=Yes, 0=No) |
| 190 | Ever_1stChgPainLocwithin109days | [Ever_1stChgPainLocwithin109days] Ever had a new pain dx within 109 days (1=Yes, 0=No) |
| 191 | FLC_LT109Days_PLC_LT7Days_GE1 | [FLC_LT109Days_PLC_LT7Days_GE1] New pain dx within 109 days, GE 1 location change within 7 days (1=Yes, 0=No) |
| 192 | FLC_LT109Days_PLC_LT30Days_GE1 | [FLC_LT109Days_PLC_LT30Days_GE1] New pain dx within 109 days, GE 1 location change within 30 days (1=Yes, 0=No) |
| 193 | FLC_LT109Days_PLC_LT90Days_GE1 | [FLC_LT109Days_PLC_LT90Days_GE1] Yes/No New pain dx within 109 days, GE 1 location change within 90 days (1=Yes, 0=No) |
| 194 | FLC_LT109Days_PLC_LT180Days_GE1 | [FLC_LT109Days_PLC_LT180Days_GE1] New pain dx within 109 days, GE 1 location change within 180 days (1=Yes, 0=No) |
| 195 | DV_E_codes_YN | [DV_E_codes_YN]Domestic Violence Related E-Codes:E967.1,E967.3,E967.9,E960-E968,E904.0 & E968.4,E980-E989 |
| 196 | DV_V_codes_YN | [DV_V_codes_YN] Domestic Violence V-Codes (history codes):V15.41,V15.42,V15.49,V61.11,V61.12 |
| 197 | HYBRID_DV_EV_codes_YN | [HYBRID_DV_EV_codes_YN]Domestic Violence related E & V codes:E967.1,E967.3,E967.9,E960-E968,E904.0 & E968.4,E980-E989; V-Codes (history codes):V15.41,V15.42,V15.49,V61.11,V61.12 |
| 198 | Ever_Alcohol_3039X | [Ever_Alcohol_3039X] Ever with alcohol dx codes [303.90,303.91,303.92,303.93,V11.3] |
| 199 | Ever_Alcohol_3030X | [Ever_Alcohol_3030X] Ever with alcohol dx codes [303.00 303.01, 303.02, 303.03] |
| 200 | Charlson_Score_GE2 | [Charlson_Score_GE2]Yes/No Charlson score@ Index date >= 2 (1=Yes,0=No) |
| 201 | Charlson_Score_PostIndex_GE2 | [Charlson_Score_PostIndex_GE2]Yes/No Charlson score@ 1 year post index >= 2 (1=Yes,0=No) |
| 202 | Pct_increase_Charlson_GE10 | [Pct_increase_Charlson_GE10]Yes/No Percentage change in Charlson score post index >= 10 (1=Yes,0=No) |
| 203 | Ever_ConcurAlcTobOpioids_1days | [Ever_ConcurAlcTobOpioids_1days] Ever concurrent (within 1 days) use of Opioid (ERLA+SA) prescription with Tobacco and/or Alcohol use |
| 204 | Ever_ConcurAlcTobOpioids_3days | [Ever_ConcurAlcTobOpioids_3days] Ever concurrent (within 3 days) use of Opioids (ERLA+SA) prescription with Tobacco and/or Alcohol use |
| 205 | Ever_ConcurAlcTobOpioids_7days | [Ever_ConcurAlcTobOpioids_7days] Ever concurrent (within 7 days) use of Opioids (ERLA+SA) prescription with Tobacco and/or Alcohol use |
| 206 | Ever_ConcurAlcTobOpioids_30days | [Ever_ConcurAlcTobOpioids_30days] Ever concurrent (within 30 days) use of Opioids (ERLA+SA) prescription with Tobacco and/or Alcohol use |
| 207 | Ever_ConcurAlcTobErla_1days | [Ever_ConcurAlcTobErla_1days] Ever concurrent (within 1 days) use of ERLA prescription with Tobacco and/or Alcohol use |
| 208 | Ever_ConcurAlcTobErla_3days | [Ever_ConcurAlcTobErla_3days] Ever concurrent (within 3 days) use of ERLA prescription with Tobacco and/or Alcohol use |
| 209 | Ever_ConcurAlcTobErla_7days | [Ever_ConcurAlcTobErla_7days] Ever concurrent (within 7 days) use of ERLA prescription with Tobacco and/or Alcohol use |
| 210 | Ever_ConcurAlcTobErla_30days | [Ever_ConcurAlcTobErla_30days] Ever concurrent (within 30 days) use of ERLA prescription with Tobacco and/or Alcohol use |
| 211 | Ever_ConcurAlcTobSA_1days | [Ever_ConcurAlcTobSA_1days] Ever concurrent (within 1 days) use of SA prescription with Tobacco and/or Alcohol use |
| 212 | Ever_ConcurAlcTobSA_3days | [Ever_ConcurAlcTobSA_3days] Ever concurrent (within 3 days) use of SA prescription with Tobacco and/or Alcohol use |
| 213 | Ever_ConcurAlcTobSA_7days | [Ever_ConcurAlcTobSA_7days] Ever concurrent (within 7 days) use of SA prescription with Tobacco and/or Alcohol use |
| 214 | Ever_ConcurAlcTobSA_30days | [Ever_ConcurAlcTobSA_30days] Ever concurrent (within 30 days) use of SA prescription with Tobacco and/or Alcohol use |
| 215 | Fibromyalgia_Ever_YN | [Fibromyalgia_Ever_YN] Ever w/ Fibromyalgia ICD9 dx 729.1x (1=Yes, 0=No) |
| 216 | CC_Opioids_Fibro_within7days | [CC_Opioids_Fibro_within7days] ERLA+SA opioids within 7 days of Fibromyalgia dx (1=Yes, 0=No) |
| 217 | CC_ERLAOpioids_Fibro_within7days | [CC_ERLAOpioids_Fibro_within7days] ERLA opioids within 7 days of Fibromyalgia dx (1=Yes, 0=No) |
| 218 | CC_SAOpioids_Fibro_within7days | [CC_SAOpioids_Fibro_within7days] SA opioids within 7 days of Fibromyalgia dx (1=Yes, 0=No) |
| 219 | Opioids_BP_Dep_within7days | [Opioids_BP_Dep_within7days] ERLA+SA opioids within 7 days of BP and Depression dx (1=Yes, 0=No) |
| 220 | ERLA_Opioid_BP_Dep_within7days | [ERLA_Opioid_BP_Dep_within7days] ERLA opioids within 7 days of BP and Depression dx(1=Yes, 0=No) |
| 221 | SA_Opioid_BP_Dep_within7days | [SA_Opioid_BP_Dep_within7days] SA opioids within 7 days of BP and Depression dx (1=Yes, 0=No) |
| 222 | Ever_ERLASAForHeadMigraine_1days | [Ever_ERLASAForHeadMigraine_1days] Ever dispensed Opioid (ERLA+SA) for headache/migraine dx( within 1 day) |
| 223 | Ever_ERLAForHeadMigraine_1days | [Ever_ERLAForHeadMigrane_1days] Ever dispensed ERLA Opioids for headache/migraine dx( within 1 day) |
| 224 | Ever_SAForHeadMigraine_1days | [Ever_SAForHeadMigraine_1days] Ever dispensed SA Opioid for headache/migraine dx( within 1 day) |
| 225 | Ever_Insomnia_dx | [Ever_Insomnia_dx] Ever diagnosed with Acute insomnia[304.41],Psychophysiolical insomina[307.42], inadequate sleep hygiene[V69.4], Insomnia due to drug or substance [292.85], Insomnia due to unspecifi |
| 226 | Ever_AcuteInsomnia_dx | [Ever_AcuteInsomnia_dx] Ever diagnosed with Acute insomnia[304.41] |
| 227 | Ever_PsychoPhysioInsomnia_dx | [Ever_PsychoPhysioInsomnia_dx] Ever diagnosed with Psychophysiolical insomina[307.42] |
| 228 | Ever_InadeqSleepHygiene_dx | [Ever_InadeqSleepHygiene_dx] Ever diagnosed inadequate sleep hygiene[V69.4] |
| 229 | Ever_InsomniaDuetoDrugSubs_dx | [Ever_InsomniaDuetoDrugSubs_dx] Ever diagnosed with Insomnia due to drug or substance [292.85] |
| 230 | Ever_InsomniaDueToMedCond_dx | [Ever_InsomniaDueToMedCond_dx] Ever diagnosed with Insomnia due to medical condition[327.01] |
| 231 | Ever_InsomniaUnspecified_dx | [Ever_InsomniaUnspecified_dx] Ever diagnosed with Insomnia NOT due to substance or known physiologic condition, unspecified[780.52] |
| 232 | Ever_PhysiologicInsomnia_dx | [Ever_PhysiologicInsomnia_dx] Ever diagnosed with Physiologic(organic) insomnia, unspecified[327.00] |
| 233 | Ever_ObstructiveSleepApnea | [Ever_ObstructiveSleepApnea] Ever diagnosed with Obstructive Sleep Apnea [327.23] |
| 234 | Ever_CentralSleepApneaSyndrome | [Ever_CentralSleepApneaSyndrome] Ever diagnosed with Central Sleep Apnea Syndromes [327.21,768.04,327.22,327.27,327.29,770.81] |
| 235 | Ever_HypoventHypoxemicSynd | [Ever_HypoventHypoxemicSynd] Ever diagnosed with sleep-realted Hypoventilation and Hypoxemic Syndromes [327.24- 324.26] |
| 236 | Ever_HypersomniasCentralOrigin | [Ever_HypersomniasCentralOrigin] Ever diagnosed with Hypersomnias of Central Origin [347.01,347.00,347.10,780.54,327.13,327.11,327.12,307.44,292.85,327.15,327.10] |
| 237 | Ever_CircadianRythmSD | [Ever_CircadianRythmSD] Ever diagnosed with Circadian Rhythm Sleep Disorder [327.31,327.32,327.33,327.34,327.35,327.36,327.39,292.85] |
| 238 | Ever_Parasomnias | [Ever_Parasomnias] Ever diagnosed with parasomnias [327.41,307.46,327.42,327.43,307.47,300.15,788.36,327.49,368.16,227.40,292.85,327.44] |
| 239 | Ever_IsolatedSleepSymptoms | [Ever_IsolatedSleepSymptoms] Ever diagnosed with isolated sleep symptoms [307.49,786.09,307.47,781.01,327.8,307.48] |
| 240 | Ever_SleepDisorder_Dx | [ Ever_SleepDisorder_Dx] Ever diagnosed with Sleep Disorder [307.4X,327.0X-327.4X, 347.0X, 780.X, 780.X etc.] |
| 241 | Ever_AllOpioids_Sleep_Disorder | [Ever_AllOpioids_Sleep_Disorder] Ever dispensed Opioid (ERLA+SA) within 1 day for sleep disorder related visit |
| 242 | Ever_ERLAOpioids_Sleep_Disorder | [Ever_ERLAOpioids_Sleep_Disorder] Ever dispensed Opioid (ERLA only) within 1 day for sleep disorder related visit |
| 243 | Ever_SAOpioids_Sleep_Disorder | [Ever_SAOpioids_Sleep_Disorder] Ever dispensed Opioid (SA only) within 1 day for sleep disorder related visit |
| 244 | Ever_AntiDep_Treat_Pain_MH_QTR | [Ever_AntiDep_Treat_Pain_MH_QTR] Ever used antidepressants to treat both Depression and Pain in same QTR |
| 245 | Ever_AntiDep_Treat_Pain_MH_MTH | [Ever_AntiDep_Treat_Pain_MH_MTH] Ever used antidepressants to treat both Depression and Pain in same MONTH |
| 246 | Ever_AntiDep_Treat_Pain_MH_WK | [Ever_AntiDep_Treat_Pain_MH_WK] Ever used antidepressants to treat both Depression and Pain in same WEEK |
| 247 | Ever_Phy_Therapy | [Ever_Phy_Therapy] Ever ahd Physical Therapy related services |
| 248 | Ever_Opioids_PhyTherapy_1day | [Ever_Opioids_PhyTherapy_1day] Ever dispensed Opioid (ERLA+SA) while on Physical Therapy treatment(within 1 day) |
| 249 | Ever_ERLA_PhyTherapy_1day | [Ever_ERLA_PhyTherapy_1day] Ever dispensed Opioid (ERLA only) while on Physical Therapy treatment(within 1 day) |
| 250 | Ever_SA_PhyTherapy_1day | [Ever_SA_PhyTherapy_1day] Ever dispensed Opioid (SA only) while on Physical Therapy treatment(within 1 day) |
| 251 | Ever_SubAbuse_DX_PX_RX | [Ever_SubAbuse_DX_PX_RX] Ever w/ diagnosis, Treatment and/or medication related to Substance Abuse |
| 252 | Ever_SubAbuse_Tmt_and_Meds | [Ever_SubAbuse_Tmt_and_Meds] Ever received Substance Abuse Treatment and medication |
| 253 | DaysDiff_SubAb_Tmt_RX | [DaysDiff_SubAb_Tmt_RX] Received medication within days of Substance Abuse Treatment |
| 254 | SubAb_Tmt_RX_LE3_Days | [SubAb_Tmt_RX_LE3_Days] Received AA treatment meds within 3 days of attending Substance Abuse Treatment program |
| 255 | SubAb_Tmt_RX_LE6_Days | [SubAb_Tmt_RX_LE6_Days] Received AA treatment meds within 6 days of attending Substance Abuse Treatment program |
| 256 | N_DistinctQtrSchizoDx |  |
| 257 | N_DistinctQtrPOUDxGE2 | [N_DistinctQtrPOUDxGE2]Ever >=2 quarters w/ Problem Opioid Dx (ICD9:304.*-305.*) |
| 258 | N_DistinctQtrOPDepDxGE2 | [N_DistinctQtrOPDepDxGE2]Ever >=2 quarters w/ Opioid Dependence dx (ICD9:304.* only) |
| 259 | N_DistinctQtrOPAbuseDxGE2 | [N_DistinctQtrOPAbuseDxGE2]Ever >=2 quarters w/ Opioid Abuse dx (ICD9:305.* only) |
| 260 | N_DistinctQtrSubAbuseDxGE6 | [N_DistinctQtrSubAbuseDxGE6]Ever >=6 quarters w/ Non-Opioid Substance Abuse (alcoholism, Specified drugs, Combination drugs,Cannibis, Tobacco dependence) |
| 261 | N_DistinctQtrAlcoholDxGE4 | [N_DistinctQtrAlcoholDxGE4]Ever >=4 quarters w/ Alcohol Abuse dx(ICD9: 303.0*, 303.9*, V11.3) |
| 262 | N_DistinctQtrTobbacoDxGE6 | [N_DistinctQtrTobbacoDxGE6]Ever >=6 quarters w/ Tobacco disorder dx(ICD9: 305.1*) |
| 263 | N_DistinctQtrMHDxGE6 | [N_DistinctQtrMHDxGE6]Ever >=6 quarters w/ Mental Health dx(ICD9: 305.1*) |
| 264 | N_DistinctQtrDepressDxGE6 | [N_DistinctQtrDepressDxGE6]Ever >=6 quarters w/ Depression dx |
| 265 | N_DistinctQtrAnxietyDxGE5 | [N_DistinctQtrAnxietyDxGE5]Ever >=5 quarters w/ Anxiety disorder dx |
| 266 | N_DistinctQtrOtherMoodDxGE2 | [N_DistinctQtrOtherMoodDxGE2]Ever >=2 quarters w/ Other Mood disorder dx |
| 267 | N_DistinctQtrPainDxGE6 | [N_DistinctQtrPainDxGE6]Ever >=6 quarters w/ Pain dx |
| 268 | N_DistinctQtrLowBackPainDxGE6 | [N_DistinctQtrLowBackPainDxGE6]Ever >=6 quarters w/ Low Back Pain dx |
| 269 | N_DistinctQtrOthBackPainDxGE6 | [N_DistinctQtrOthBackPainDxGE6]Ever >=6 quarters w/ Other Back Pain dx |
| 270 | N_DistinctQtrHeadacheDxGE6 | [N_DistinctQtrHeadacheDxGE6]Ever >=6 quarters w/ Headache or Migraine dx |
| 271 | N_DistinctQtrFibroDxGE5 | [N_DistinctQtrFibroDxGE5]Ever >=5 quarters w/ Fibromyalgia dx |
| 272 | N_DistinctQtrArthDxGE6 | [N_DistinctQtrArthDxGE6]Ever >=6 quarters w/ Arthritis dx |
| 273 | MaxVisitsAnyQtr_AlcoholDxGE2 | [MaxVisitsAnyQtr_AlcoholDxGE2]Ever >=2 visits in any qtr w/ Alcohol Abuse dx(ICD9: 303.0*, 303.9*, V11.3) |
| 274 | MaxVisitsAnyQtr_SpecDrugDepDxGE2 | [MaxVisitsAnyQtr_SpecDrugDepDxGE2]Ever >=2 visits in any qtr w/ Specified Drug Dependence dx(ICD9: 304.*) |
| 275 | MaxVisitsAnyQtr_CanDepDxGE2 | [MaxVisitsAnyQtr_CanDepDxGE2]Ever >=2 visits in any qtr w/ Cannabis Dependence dx(ICD9: 304.3*, 305.2*) |
| 276 | MaxVisitsAnyQtr_NDADxGE2 | [MaxVisitsAnyQtr_NDADxGE2]Ever >=2 visits in any qtr w/ Nondependent Drug Abuse dx(ICD9: 305.3*-305.9*) |
| 277 | MaxVisitsAnyQtr_HepaCirrDxGE3 | [MaxVisitsAnyQtr_HepaCirrDxGE3]Ever >=3 visits in any qtr w/ Hepatitis or Cirrhosis disorder dx |
| 278 | MaxVisitsAnyQtr_BipolarDxGE3 | [MaxVisitsAnyQtr_BipolarDxGE3]Ever >=3 visits in any qtr w/ Bipolar dx |
| 279 | MaxVisitsAnyQtr_OtherMHDxGE3 | [MaxVisitsAnyQtr_OtherMHDxGE3]Ever >=3 visits in any qtr w/ Other Mental Health disorder dx |
| 280 | MaxVisitsAnyQtr_ComDrugDepDxGE2 | [MaxVisitsAnyQtr_ComboDrugDepDxGE2]Ever >=2 visits in any qtr w/ Combination of drug dependence dx(ICD9: 304.8*, 305.9*) |
| 281 | Pct_QtrPOUDxGE8 | [Pct_QtrPOUDxGE8]Ever >=8% Qtrs w/ Problem Opioid Dx (ICD9:304.*-305.*) |
| 282 | Pct_QtrOPDepDxGE18 | [Pct_QtrOPDepDxGE18]Ever >=18% Qtrs w/ Opioid Dependence dx (ICD9:304.* only) |
| 283 | Pct_QtrSubAbuseDxGE25 | [Pct_QtrSubAbuseDxGE25]Ever >=25% Qtrs w/ Non-Opioid Substance Abuse (alcoholism, Specified drugs, Combination drugs,Cannibis, Tobacco dependence) |
| 284 | Pct_QtrAlcoholDxGE18 | [Pct_QtrAlcoholDxGE18]Ever >=18% Qtrs w/ Alcohol Abuse dx(ICD9: 303.0*, 303.9*, V11.3) |
| 285 | Pct_QtrComboDrugDepDxGE2 | [Pct_QtrComboDrugDepDxGE2]Ever >=2% Qtrs w/ Combination of drug dependence dx(ICD9: 304.8*, 305.9*) |
| 286 | Pct_QtrNDADxGE4 | [Pct_QtrNDADxGE4]Ever >=4% Qtrs w/ Nondependent Drug Abuse dx(ICD9: 305.3*-305.9*) |
| 287 | Pct_QtrTobbacoDxGE10 | [Pct_QtrTobbacoDxGE10]Ever >=10% Qtrs w/ Tobacco disorder dx(ICD9: 305.1*) |
| 288 | Pct_QtrMHDxGE17 | [Pct_QtrMHDxGE17]Ever >=17% Qtrs w/ Mental Health dx(ICD9: 305.1*) |
| 289 | Pct_QtrDepressDxGE22 | [Pct_QtrDepressDxGE22]Ever >=22% Qtrs w/ Depression dx |
| 290 | Pct_QtrAnxietyDxGE10 | [Pct_QtrAnxietyDxGE10]Ever >=10% Qtrs w/ Anxiety disorder dx |
| 291 | Pct_QtrOtherMoodDxGE8 | [Pct_QtrOtherMoodDxGE8]Ever >=8% Qtrs w/ Other Mood disorder dx |
| 292 | Pct_QtrPainDxGE58 | [Pct_QtrPainDxGE58]Ever >=58% Qtrs w/ Pain dx |
| 293 | Pct_QtrLowBackPainDxGE38 | [Pct_QtrLowBackPainDxGE38]Ever >=38% Qtrs w/ Low Back Pain dx |
| 294 | Pct_QtrOthBackPainDxGE20 | [Pct_QtrOthBackPainDxGE20]Ever >=20% Qtrs w/ Other Back Pain dx |
| 295 | Pct_QtrHeadacheDxGE17 | [Pct_QtrHeadacheDxGE17]Ever >=17% Qtrs w/ Headache or Migraine dx |
| 296 | Pct_QtrFibroDxGE8 | [Pct_QtrFibroDxGE8]Ever >=8% Qtrs w/ Fibromialgia dx |
| 297 | Pct_QtrArthDxGE38 | [Pct_QtrArthDxGE38]Ever >=38% Qtrs w/ Arthritis dx |
| 298 | GENDER_MALE |  |
| 299 | GENDER_FEMALE |  |
| 300 | I_ICD9xSubAbTmtRXLE3Days | [I_ICD9xSubAbTmtRXLE3Days] Interaction: ICD9_YES x SubAb_Tmt_RX_LE3_Days |
| 301 | I_ICD9xSubAbTmtRXLE6Days | [I_ICD9xSubAbTmtRXLE6Days] Interaction: ICD9_YES x SubAb_Tmt_RX_LE6_Days |
| 302 | I_AGEGE46LE64xSubAbTmtRXLE3Days | [I_AGEGE46LE64xSubAbTmtRXLE3Days] Interaction: AGE_GE46_LE64 x SubAb_Tmt_RX_LE3_Days |
| 303 | I_AGEGE65xSubAbTmtRXLE3Days | [I_AGEGE65xSubAbTmtRXLE3Days] Interaction: AGE_GE65 x SubAb_Tmt_RX_LE3_Days |
| 304 | I_AGEGE46LE64xSubAbTmtRXLE6Days | [I_AGEGE46LE64xSubAbTmtRXLE6Days] Interaction: AGE_GE46_LE64 x SubAb_Tmt_RX_LE6_Days |
| 305 | I_AGEGE65xSubAbTmtRXLE6Days | [I_AGEGE65xSubAbTmtRXLE6Days] Interaction: AGE_GE65 x SubAb_Tmt_RX_LE6_Days |
| 306 | I_AGEGE46LE64_Ever_Alcohol_3039X | [I_AGEGE46LE64_Ever_Alcohol_3039X] Interaction: Interaction: AGE_GE46_LE64 x Ever_Alcohol_3039X |
| 307 | I_AGEGE46LE64_AlcoholDx_Ever_YN | [I_AGEGE46LE64_AlcoholDx_Ever_YN] Interaction: Interaction: AGE_GE46_LE64 x AlcoholDx_Ever_YN |
| 308 | I_AGELE45_NDistQtrSubAbuseDxGE6 | [I_AGELE45_NDistQtrSubAbuseDxGE6] Interaction: Interaction: AGE_LE45 x N_DistinctQtrSubAbuseDxGE6 |
| 309 | I_AGEGE46LE64_Pct_QtrOPDepDxGE18 | [I_AGEGE46LE64_Pct_QtrOPDepDxGE18] Interaction: AGE_GE46_LE64 x Pct_QtrOPDepDxGE18 |
| 310 | I_AGEGE46LE64_OPDep_30401_YN | [I_AGEGE46LE64_OPDep_30401_YN] Interaction: AGE_GE46_LE64 x OPDep_30401_YN |
| 311 | I_AGEGE46LE64_NDistQtrTobDxGE6 | [I_AGEGE46LE64_NDistQtrTobDxGE6] Interaction: AGE_GE46_LE64 x N_DistinctQtrTobbacoDxGE6 |
| 312 | I_AGEGE65_Pct_QtrAlcoholDxGE18 | [I_AGEGE65_Pct_QtrAlcoholDxGE18] Interaction: AGE_GE65 x Pct_QtrAlcoholDxGE18 |
| 313 | I_AGEGE46LE64xAlc30393YN | [I_AGEGE46LE64xAlc30393YN] Interaction: AGE_GE46_LE64 * Alcohol_ICD9_30393_YN |
| 314 | I_AGEGE65xAlc30393YN | [I_AGEGE65xAlc30393YN] Interaction: AGE_GE65 * Alcohol_ICD9_30393_YN |
| 315 | I_AGEGE46LE64xDRUGDEPNOSEVER | [I_AGEGE46LE64xDRUGDEPNOSEVER] Interaction: AGE_GE46_LE64 * DRUG_DEP_NOS_EVER |
| 316 | I_AGEGE65xDRUGDEPNOSEVER | [I_AGEGE65xDRUGDEPNOSEVE] Interaction: AGE_GE65 * DRUG_DEP_NOS_EVER |
| 317 | I_AGEGE46LE64xNoofDistPainGE3 | [I_AGEGE46LE64xNoofDistPainGE3] Interaction: AGE_GE46_LE64 * Ever_NoofDistinctPainLoc_GE3 |
| 318 | I_AGEGE65xNoofDistPainGE3 | [I_AGEGE65xNoofDistPainGE3] Interaction: AGE_GE65 * Ever_NoofDistinctPainLoc_GE3 |
| 319 | I_AGEGE46LE64xNoofDistPainGE4 | [I_AGEGE46LE64xNoofDistPainGE4] Interaction: AGE_GE46_LE64 * Ever_NoofDistinctPainLoc_GE4 |
| 320 | I_AGEGE65xNoofDistPainGE4 | [I_AGEGE65xNoofDistPainGE4] Interaction: AGE_GE65 * Ever_NoofDistinctPainLoc_GE4 |
| 321 | I_AGEGE46LE64xNoofDistPainGE5 | [I_AGEGE46LE64xNoofDistPainGE5] Interaction: AGE_GE46_LE64 * Ever_NoofDistinctPainLoc_GE5 |
| 322 | I_AGEGE65xNoofDistPainGE5 | [I_AGEGE65xNoofDistPainGE5] Interaction: AGE_GE65 * Ever_NoofDistinctPainLoc_GE5 |
| 323 | I_AGEGE46LE64xNoofDistPainGE6 | [I_AGEGE46LE64xNoofDistPainGE6] Interaction: AGE_GE46_LE64 * Ever_NoofDistinctPainLoc_GE6 |
| 324 | I_AGEGE65xNoofDistPainGE6 | [I_AGEGE65xNoofDistPainGE6] Interaction: AGE_GE65 * Ever_NoofDistinctPainLoc_GE6 |
| 325 | I_AGEGE46LE64xAlc30390YN | [I_AGEGE46LE64xAlc30390YN] Interaction: AGE_GE46_LE64 * Alcohol_ICD9_30390_YN |
| 326 | I_AGEGE65xAlc30390YN | [I_AGEGE65xAlc30390YN] Interaction: AGE_GE65 * Alcohol_ICD9_30390_YN |
| 327 | I_AGEGE46LE64xComboDrugDepYN | [I_AGEGE46LE64xComboDrugDepYN] Interaction: AGE_GE46_LE64 * ComboDrugDepDx_Ever_YN |
| 328 | I_AGEGE65xComboDrugDepYN | [I_AGEGE65xComboDrugDepYN] Interaction: AGE_GE65 * ComboDrugDepDx_Ever_YN |
| 329 | I_AGEGE46LE64xEverAlc3039X | [I_AGEGE46LE64xEverAlc3039X] Interaction: AGE_GE46_LE64 * Ever_Alcohol_3039X |
| 330 | I_AGEGE65xEverAlc3039X | [I_AGEGE65xEverAlc3039X] Interaction: AGE_GE65 * Ever_Alcohol_3039X |
| 331 | I_AGEGE46LE64xPctQtrPOUDxGE8 | [I_AGEGE46LE64xPctQtrPOUDxGE8] Interaction: AGE_GE46_LE64 * Pct_QtrPOUDxGE8 |
| 332 | I_AGEGE65xPctQtrPOUDxGE8 | [I_AGEGE65xPctQtrPOUDxGE8] Interaction: AGE_GE65 * Pct_QtrPOUDxGE8 |
| 333 | I_AGEGE46LE64xAlcoholDxYN | [I_AGEGE46LE64xAlcoholDxYN] Interaction: AGE_GE46_LE64 * AlcoholDx_Ever_YN |
| 334 | I_AGEGE65xAlcoholDxYN | [I_AGEGE65xAlcoholDxYN] Interaction: AGE_GE65 * AlcoholDx_Ever_YN |
| 335 | I_AGEGE46LE64xPctQtrOPDepDxGE18 | [I_AGEGE46LE64xPctQtrOPDepDxGE18] Interaction: AGE_GE46_LE64 * Pct_QtrOPDepDxGE18 |
| 336 | I_AGEGE65xPctQtrOPDepDxGE18 | [I_AGEGE65xPctQtrOPDepDxGE18] Interaction: AGE_GE65 * Pct_QtrOPDepDxGE18 |
| 337 | I_AGEGE46LE64xNDistQtrAnxietyGE5 | [I_AGEGE46LE64xNDistQtrAnxietyGE5] Interaction: AGE_GE46_LE64 * N_DistinctQtrAnxietyDxGE5 |
| 338 | I_AGEGE65xNDistQtrAnxietyGE5 | [I_AGEGE65xNDistQtrAnxietyGE5] Interaction: AGE_GE65 * N_DistinctQtrAnxietyDxGE5 |
| 339 | I_AGEGE46LE64xPctQtrSubAbDxGE25 | [I_AGEGE46LE64xPctQtrSubAbDxGE25] Interaction: AGE_GE46_LE64 * Pct_QtrSubAbuseDxGE25 |
| 340 | I_AGEGE65xPctQtrSubAbDxGE25 | [I_AGEGE65xPctQtrSubAbDxGE25] Interaction: AGE_GE65 * Pct_QtrSubAbuseDxGE25 |
| 341 | I_AGEGE46LE64xNDistQtrSubAbGE6 | [I_AGEGE46LE64xNDistQtrSubAbGE6] Interaction: AGE_GE46_LE64 * N_DistinctQtrSubAbuseDxGE6 |
| 342 | I_AGEGE65xNDistQtrSubAbGE6 | [I_AGEGE65xNDistQtrSubAbGE6] Interaction: AGE_GE65 * N_DistinctQtrSubAbuseDxGE6 |
| 343 | I_AGEGE46LE64xOPDep30400YN | [I_AGEGE46LE64xOPDep30400YN] Interaction: AGE_GE46_LE64 * OPDep_30400_YN |
| 344 | I_AGEGE65xOPDep30400YN | [I_AGEGE65xOPDep30400YN] Interaction: AGE_GE65 * OPDep_30400_YN |
| 345 | I_AGEGE46LE64xPctQtrHeadDxGE17 | [I_AGEGE46LE64xPctQtrHeadDxGE17] Interaction: AGE_GE46_LE64 * Pct_QtrHeadacheDxGE17 |
| 346 | I_AGEGE65xPctQtrHeadDxGE17 | [I_AGEGE65xPctQtrHeadDxGE17] Interaction: AGE_GE65 * Pct_QtrHeadacheDxGE17 |
| 347 | I_AGEGE46LE64xOPDepDxEverYN | [I_AGEGE46LE64xOPDepDxEverYN] Interaction: AGE_GE46_LE64 * OPDepDx_Ever_YN |
| 348 | I_AGEGE65xOPDepDxEverYN | [I_AGEGE65xOPDepDxEverYN] Interaction: AGE_GE65 * OPDepDx_Ever_YN |
| 349 | I_AGEGE46LE64xCCAlcTobErla1days | [I_AGEGE46LE64xCCAlcTobErla1days] Interaction: AGE_GE46_LE64 * Ever_ConcurAlcTobErla_1days |
| 350 | I_AGEGE65xCCAlcTobErla1days | [I_AGEGE65xCCAlcTobErla1days] Interaction: AGE_GE65 * Ever_ConcurAlcTobErla_1days |
| 351 | I_AGEGE46LE64xOPDep30401YN | [I_AGEGE46LE64xOPDep30401YN] Interaction: AGE_GE46_LE64 * OPDep_30401_YN |
| 352 | I_AGEGE65xOPDep30401YN | [I_AGEGE65xOPDep30401YN] Interaction: AGE_GE65 * OPDep_30401_YN |
| 353 | I_AGEGE46LE64xCCAlcTobSA1days | [I_AGEGE46LE64xCCAlcTobSA1days] Interaction: AGE_GE46_LE64 * Ever_ConcurAlcTobSA_1days |
| 354 | I_AGEGE65xCCAlcTobSA1days | [I_AGEGE65xCCAlcTobSA1days] Interaction: AGE_GE65 * Ever_ConcurAlcTobSA_1days |
| 355 | I_AGEGE46LE64xSAForMigraine1days | [I_AGEGE46LE64xSAForMigraine1days] Interaction: AGE_GE46_LE64 * Ever_SAForHeadMigraine_1days |
| 356 | I_AGEGE65xSAForMigraine1days | [I_AGEGE65xSAForMigraine1days] Interaction: AGE_GE65 * Ever_SAForHeadMigraine_1days |
| 357 | I_AGEGE46LE64xCCAlcTobErla3days | [I_AGEGE46LE64xCCAlcTobErla3days] Interaction: AGE_GE46_LE64 * Ever_ConcurAlcTobErla_3days |
| 358 | I_AGEGE65xCCAlcTobErla3days | [I_AGEGE65xCCAlcTobErla3days] Interaction: AGE_GE65 * Ever_ConcurAlcTobErla_3days |
| 359 | I_AGEGE46LE64xPctQtrAnxietyGE10 | [I_AGEGE46LE64xPctQtrAnxietyGE10] Interaction: AGE_GE46_LE64 * Pct_QtrAnxietyDxGE10 |
| 360 | I_AGEGE65xPctQtrAnxietyGE10 | [I_AGEGE65xPctQtrAnxietyGE10] Interaction: AGE_GE65 * Pct_QtrAnxietyDxGE10 |
| 361 | I_AGEGE46LE64xCCAlcTobSA30days | [I_AGEGE46LE64xCCAlcTobSA30days] Interaction: AGE_GE46_LE64 * Ever_ConcurAlcTobSA_30days |
| 362 | I_AGEGE65xCCAlcTobSA30days | [I_AGEGE65xCCAlcTobSA30days] Interaction: AGE_GE65 * Ever_ConcurAlcTobSA_30days |
| 363 | I_AGEGE46LE64xCCAlcTobOp1days | [I_AGEGE46LE64xCCAlcTobOp1days] Interaction: AGE_GE46_LE64 * Ever_ConcurAlcTobOpioids_1days |
| 364 | I_AGEGE65xCCAlcTobOp1days | [I_AGEGE65xCCAlcTobOp1days] Interaction: AGE_GE65 * Ever_ConcurAlcTobOpioids_1days |
| 365 | I_AGEGE46LE64xCCAlcTobSA3days | [I_AGEGE46LE64xCCAlcTobSA3days] Interaction: AGE_GE46_LE64 * Ever_CCAlcTobSA_3days |
| 366 | I_AGEGE65xConcurAlcTobSA3days | [I_AGEGE65xConcurAlcTobSA3days] Interaction: AGE_GE65 * Ever_ConcurAlcTobSA_3days |
| 367 | I_AGEGE46LE64xCCAlcTobSA7days | [I_AGEGE46LE64xCCAlcTobSA7days] Interaction: AGE_GE46_LE64 * Ever_ConcurAlcTobSA_7days |
| 368 | I_AGEGE65xCCAlcTobSA7days | [I_AGEGE65xCCAlcTobSA7days] Interaction: AGE_GE65 * Ever_ConcurAlcTobSA_7days |
| 369 | I_AGEGE46LE64xPctQtrTobDxGE10 | [I_AGEGE46LE64xPctQtrTobDxGE10] Interaction: AGE_GE46_LE64 * Pct_QtrTobbacoDxGE10 |
| 370 | I_AGEGE65xPctQtrTobbacoDxGE10 | [I_AGEGE65xPctQtrTobbacoDxGE10] Interaction: AGE_GE65 * Pct_QtrTobbacoDxGE10 |
| 371 | I_AGEGE46LE64xCCAlcTobOp3days | [I_AGEGE46LE64xCCAlcTobOp3days] Interaction: AGE_GE46_LE64 * Ever_ConcurAlcTobOpioids_3days |
| 372 | I_AGEGE65xCCAlcTobOp3days | [I_AGEGE65xCCAlcTobOp3days] Interaction: AGE_GE65 * Ever_ConcurAlcTobOpioids_3days |
| 373 | I_AGEGE46LE64xCCAlcTobOp7days | [I_AGEGE46LE64xCCAlcTobOp7days] Interaction: AGE_GE46_LE64 * Ever_ConcurAlcTobOpioids_7days |
| 374 | I_AGEGE65xCCAlcTobOp7days | [I_AGEGE65xCCAlcTobOp7days] Interaction: AGE_GE65 * Ever_ConcurAlcTobOpioids_7days |
| 375 | I_AGEGE46LE64xCCAlcTobErla7days | [I_AGEGE46LE64xCCAlcTobErla7days] Interaction: AGE_GE46_LE64 * Ever_ConcurAlcTobErla_7days |
| 376 | I_AGEGE65xCCAlcTobErla7days | [I_AGEGE65xCCAlcTobErla7days] Interaction: AGE_GE65 * Ever_ConcurAlcTobErla_7days |
| 377 | I_AGEGE46LE64xCCAlcTobOp30days | [I_AGEGE46LE64xCCAlcTobOp30days] Interaction: AGE_GE46_LE64 * Ever_ConcurAlcTobOpioids_30days |
| 378 | I_AGEGE65xCCAlcTobOp30days | [I_AGEGE65xCCAlcTobOp30days] Interaction: AGE_GE65 * Ever_ConcurAlcTobOpioids_30days |
| 379 | I_AGEGE46LE64xHepaCirrDxEverYN | [I_AGEGE46LE64xHepaCirrDxEverYN] Interaction: AGE_GE46_LE64 * HepaCirrDx_Ever_YN |
| 380 | I_AGEGE65xHepaCirrDxEverYN | [I_AGEGE65xHepaCirrDxEverYN] Interaction: AGE_GE65 * HepaCirrDx_Ever_YN |
| 381 | I_AGEGE46LE64xHeadtoOther1wkYN | [I_AGEGE46LE64xHeadtoOther1wkYN] Interaction: AGE_GE46_LE64 * HeadtoOther_1wk_YN |
| 382 | I_AGEGE65xHeadtoOther1wkYN | [I_AGEGE65xHeadtoOther1wkYN] Interaction: AGE_GE65 * HeadtoOther_1wk_YN |
| 383 | I_AGEGE46LE64xOpForHead1days | [I_AGEGE46LE64xOpForHead1days] Interaction: AGE_GE46_LE64 * Ever_ERLASAForHeadMigraine_1days |
| 384 | I_AGEGE65xOpForHead1days | [I_AGEGE65xOpForHead1days] Interaction: AGE_GE65 * Ever_ERLASAForHeadMigraine_1days |
| 385 | I_AGEGE46LE64xERLASleepDO | [I_AGEGE46LE64xERLASleepDO] Interaction: AGE_GE46_LE64 * Ever_ERLAOpioids_Sleep_Disorder |
| 386 | I_AGEGE65xERLASleepDO | [I_AGEGE65xERLASleepDO] Interaction: AGE_GE65 * Ever_ERLAOpioids_Sleep_Disorder |
| 387 | I_AGEGE46LE64xCCAlcTobErla30days | [I_AGEGE46LE64xCCAlcTobErla30days] Interaction: AGE_GE46_LE64 * Ever_ConcurAlcTobErla_30days |
| 388 | I_AGEGE65xCCAlcTobErla30days | [I_AGEGE65xCCAlcTobErla30days] Interaction: AGE_GE65 * Ever_ConcurAlcTobErla_30days |
| 389 | I_AGEGE46LE64xPctincCharlsonGE10 | [I_AGEGE46LE64xPctincCharlsonGE10] Interaction: AGE_GE46_LE64 * Pct_increase_Charlson_GE10 |
| 390 | I_AGEGE65xPctincCharlsonGE10 | [I_AGEGE65xPctincCharlsonGE10] Interaction: AGE_GE65 * Pct_increase_Charlson_GE10 |
| 391 | I_AGEGE46LE64xSAFibrowithin7days | [I_AGEGE46LE64xSAFibrowithin7days] Interaction: AGE_GE46_LE64 * CC_SAOpioids_Fibro_within7days |
| 392 | I_AGEGE65xSAFibrowithin7days | [I_AGEGE65xSAFibrowithin7days]AGE_GE65 * CC_SAOpioids_Fibro_within7days |
| 393 | I_AGEGE46LE64xOpsFibro7days | [I_AGEGE46LE64xOpsFibro7days] Interaction: AGE_GE46_LE64 * CC_Opioids_Fibro_within7days |
| 394 | I_AGEGE65xOpsFibro7days | [I_AGEGE65xOpsFibro7days] Interaction: AGE_GE65 * CC_Opioids_Fibro_within7days |
| 395 | I_MALE_BipolarDxYN | [I_MALE_BipolarDxYN] Interaction: GENDER_MALE x BipolarDx_Ever_YN |
| 396 | I_FEMALE_BipolarDxYN | [I_FEMALE_BipolarDxYN] Interaction: GENDER_FEMALE x Fibromyalgia_Ever_YN |
| 397 | I_MALE_FibroDxYN | [I_MALE_FibroDxYN] Interaction: GENDER_MALE x BipolarDx_Ever_YN |
| 398 | I_FEMALE_FibroDxYN | [I_FEMALE_FibroDxYN] Interaction: GENDER_FEMALE x Fibromyalgia_Ever_YN |
| 399 | I_MALE_AGELE45_AlcoholDxYN | [I_MALE_AGELE45_AlcoholDxYN] Interaction: GENDER_MALE x AGE_LE45 x AlcoholDx_Ever_YN |
| 400 | I_FEMALE_AGELE45_AlcoholDxYN | [I_FEMALE_AGELE45_AlcoholDxYN] Interaction: GENDER_FEMALE x AGE_LE45 x AlcoholDx_Ever_YN |
| 401 | I_MALE_AGEGE46LE64_AlcoholDxYN | [I_MALE_AGEGE46LE64_AlcoholDxYN] Interaction: GENDER_MALE x AGE_GE46_LE64 x AlcoholDx_Ever_YN |
| 402 | I_FEMALE_AGEGE46LE64_AlcoholDxYN | [I_FEMALE_AGEGE46LE64_AlcoholDxYN] Interaction: GENDER_FEMALE x AGE_GE46_LE64 x AlcoholDx_Ever_YN |
| 403 | I_MALE_AGEGE65_AlcoholDxYN | [I_MALE_AGEGE65_AlcoholDxYN] Interaction: GENDER_MALE x AGE_GE65 x AlcoholDx_Ever_YN |
| 404 | I_FEMALE_AGEGE65_AlcoholDxYN | [I_FEMALE_AGEGE65_AlcoholDxYN] Interaction: GENDER_FEMALE x AGE_GE65 x AlcoholDx_Ever_YN |
| 405 | ER_DURING_BENZO_RX_COUNT_GE4 | Yes/no >=4 ER encounters during active possession of benzodiazepine |
| 406 | RX_TREAT_AA_EVER_N_QTRS_GE4 | Yes/no >=4 quarters with drugs used to treat opioid abuse/addiction |
| 407 | COUNT_RXSUP_ANY_LE14_QTR_GE7 | Yes/no >=7 fills (ER or SA) with 1-14 days supply during a quarter |
| 408 | COUNT_RXSUP_ANY_LE14_QTR_GE12 | Yes/no >=12 fills (ER or SA) with 1-14 days supply during a quarter |
| 409 | COUNT_RXSUP_ER_LE7_QTR_GE1 | Ever/never >=1 ER fill with 1-7 days supply during a quarter |
| 410 | COUNT_RXSUP_ER_LE7_QTR_GE2 | Yes/no >=2 ER fill with 1-7 days supply during a quarter |
| 411 | COUNT_RXSUP_ER_8LE14_QTR_GE1 | Ever/never >=1 ER fill with 8-14 days supply during a quarter |
| 412 | COUNT_RXSUP_ER_8LE14_QTR_GE3 | Yes/no >=3 ER fill with 8-14 days supply during a quarter |
| 413 | COUNT_RXSUP_ER_LE14_QTR_GE1 | Ever/never >=1 ER fill with 1-14 days supply during a quarter |
| 414 | COUNT_RXSUP_ER_LE14_QTR_GE3 | Yes/no >=3 ER fill with 1-14 days supply during a quarter |
| 415 | COUNT_RXSUP_ER_LE14_QTR_GE5 | Yes/no >=5 ER fill with 1-14 days supply during a quarter |
| 416 | COUNT_RXSUP_LE3_WKND_ANY_GE2 | Yes/no >=2 fills (ER or SA) with 1-3 days supply dispensed on Saturday, Sunday or Monday (ever in 36 months) |
| 417 | COUNT_RXSUP_LE14_WKND_ER_GE1 | Ever/never >=1 ER fill with 1-14 days supply dispensed on Saturday, Sunday or Monday during a quarter |
| 418 | COUNT_RXSUP_LE14_WKND_ER_GE2 | Yes/no >=2 ER fills with 1-14 days supply dispensed on Saturday, Sunday or Monday (ever in 36 months) |
| 419 | COUNT_RXSUP_LE7_WKND_ER_GE1 | Ever/never >=1 ER fill with 1-7 days supply dispensed on Saturday, Sunday or Monday during a quarter |
| 420 | COUNT_RXSUP_8LE14_WKND_ER_GE1 | Ever/never >=1 ER fill with 8-14 days supply dispensed on Saturday, Sunday or Monday during a quarter |
| 421 | COUNT_RXSUP_8LE14_WKND_ER_GE2 | Yes/no >=2 ER fill with 8-14 days supply dispensed on Saturday, Sunday or Monday during a quarter |
| 422 | COUNT_RXSUP_8LE14_WKND_ER_GE3 | Yes/no >=3 ER fill with 8-14 days supply dispensed on Saturday, Sunday or Monday (ever in 36 months) |
| 423 | COUNT_RXSUP_ANY_LE14_GE21 | Yes/no >=21 opioid fills (ER or SA) with 1-14 days supply (ever in 36 months) |
| 424 | COUNT_RXSUP_ANY_LE14_GE45 | Yes/no >=21 opioid fills (ER or SA) with 1-14 days supply (ever in 36 months) |
| 425 | COUNT_RXSUP_ER_LE7_GE3 | Yes/no >=2 ER fills with 1-7 days supply (ever in 36 months) |
| 426 | COUNT_RXSUP_ER_GE8LE14_GE5 | Yes/no >=5 ER fills with 8-14 days supply (ever in 36 months) |
| 427 | COUNT_RXSUP_ER_GE8LE14_GE7 | Yes/no >=7 ER fills with 8-14 days supply (ever in 36 months) |
| 428 | COUNT_RXSUP_ER_LE14_GE6 | Yes/no >=6 ER fills with 1-14 days supply (ever in 36 months) |
| 429 | COUNT_RXSUP_ER_LE14_GE9 | Yes/no >=9 ER fills with 1-14 days supply (ever in 36 months) |
| 430 | COUNT_RXSUP_ER_LE28_GE17 | Yes/no >=17 ER fills with 1-28 days supply (ever in 36 months) |
| 431 | COUNT_RXSUP_ER_LE28_GE30 | Yes/no >=30 ER fills with 1-28 days supply (ever in 36 months) |
| 432 | OVERLAP_SUP_LE3_WKND_GE1 | Ever/never >=1 opioid fill (ER or SA) with 1-3 days supply that is dispensed on Saturday, Sunday or Monday and overlaps with a preceding fill (of any days supply) (in 36 months) |
| 433 | OVERLAP_SUP_LE3_WKND_GE2 | Yes/no >=2 opioid fills (ER or SA) with 1-3 days supply that is dispensed on Saturday, Sunday or Monday and overlaps with a preceding fill (of any days supply) (ever in 36 months) |
| 434 | OVERLAP_SUP_LE3_WKND_QTR_GE2 | Yes/no >=2 opioid fills (ER or SA) with 1-3 days supply that is dispensed on Saturday, Sunday or Monday and overlaps with a preceding fill (of any days supply) during a quarter |
| 435 | OVERLAP_SUP_LE14_ER_WKND_GE1 | Ever/never >=1 ER fill with 1-14 days supply dispensed on a Saturday, Sunday or Monday that overlaps with a preceding fill (of any days supply) |
| 436 | OVERLAP_SUP_LE14_ER_WKND_GE2 | Yes/no >=2 ER fills with 1-14 days supply dispensed on a Saturday, Sunday or Monday that overlaps with a preceding fill (of any days supply) during a quarter |
| 437 | OVERLAP_SUP_LE7_ER_WKND_GE1 | Ever/never >=1 ER fill with 1-7 days supply dispensed on a Saturday, Sunday or Monday that overlaps with a preceding fill (of any days supply) |
| 438 | OVERLAP_SUP_8LE14_ER_WKND_GE1 | Ever/never >=1 ER fill with 8-14 days supply dispensed on a Saturday, Sunday or Monday that overlaps with a preceding fill (of any days supply) |
| 439 | OVERLAP_SUP_LE14_SA_WKND_GE2 | Yes/no >=2 SA fills with 1-14 days supply dispensed on a Saturday, Sunday or Monday that overlaps with a preceding fill (of any days supply) during a quarter |
| 440 | OVERLAP_SUP_LE14_SA_WKND_GE3 | Yes/no >=3 SA fills with 1-14 days supply dispensed on a Saturday, Sunday or Monday that overlaps with a preceding fill (of any days supply) during a quarter |
| 441 | OVERLAP_SUP_LE7_SA_WKND_GE2 | Yes/no >=2 SA fills with 1-7 days supply dispensed on a Saturday, Sunday or Monday that overlaps with a preceding fill (of any days supply) during a quarter |
| 442 | OVERLAP_SUP_8LE14_SA_WKND_GE1 | Ever/never >=1 SA fill with 8-14 days supply dispensed on a Saturday, Sunday or Monday that overlaps with a preceding fill (of any days supply) |
| 443 | EXCESS_RXSUP_TOTAL_QTRS_GE2 | Yes/no >=2 quarters with >1.2 excess days supply (of ER or SA opioids) during the quarter |
| 444 | PCNT_RXSUP_ER_LE14_QTR_GE80 | Yes/no >=80% of all overlapping ER fills have days supply <=14 during a quarter |
| 445 | PCNT_RXSUP_ER_LE14_QTR_GE55 | Yes/no ER fills with <=14 days supply comprise >=55% of total days supply during a quarter |
| 446 | COUNT_RXSUP_ER_LE14_QTR_GE2 | Yes/no >=2 ER fills with <=14 days supply during a quarter |
| 447 | OVERLAP_DAYS_SUM_SA_GE60 | Yes/no >=60 cumulative excess days supply of SA opioids during a quarter |
| 448 | OVERLAP_COUNT_QTR_GE9 | Yes/no >=9 overlapping fills (ER or SA) during a quarter |
| 449 | OVERLAP_COUNT_QTR_GE12 | Yes/no >=12 overlapping fills (ER or SA) during a quarter |
| 450 | OVERLAP_COUNT_ER_GE4 | Yes/no >=4 overlapping ER fills during a quarter |
| 451 | OVERLAP_COUNT_ER_GE5 | Yes/no >=5 overlapping ER fills during a quarter |
| 452 | OVERLAP_COUNT_ER_GE6 | Yes/no >=6 overlapping ER fills during a quarter |
| 453 | OVERLAP_COUNT_ER_GE8 | Yes/no >=8 overlapping ER fills during a quarter |
| 454 | OVERLAP_COUNT_SA_GE4 | Yes/no >=4 overlapping SA fills during a quarter |
| 455 | OVERLAP_COUNT_SA_GE6 | Yes/no >=6 overlapping SA fills during a quarter |
| 456 | OVERLAP_COUNT_SA_GE8 | Yes/no >=8 overlapping SA fills during a quarter |
| 457 | OVERLAP_DAYS_SUM_ER_GE24 | Yes/no >=24 cumulative excess days supply of ER opioids during a quarter |
| 458 | OVERLAP_DAYS_SUM_ER_GE44 | Yes/no >=44 cumulative excess days supply of ER opioids during a quarter |
| 459 | OVERLAP_DAYS_SUM_SA_GE20 | Yes/no >=20 cumulative excess days supply of SA opioids during a quarter |
| 460 | OVERLAP_DAYS_SUM_SA_GE41 | Yes/no >=41 cumulative excess days supply of SA opioids during a quarter |
| 461 | OVERLAP_DAYS_SUM_SA_GE45 | Yes/no >=45 cumulative excess days supply of SA opioids during a quarter |
| 462 | OVERLAP_SUPER_LE7_GE2 | Yes/no >=2 overlapping ER fills with <=7 days supply during a quarter |
| 463 | OVERLAP_SUPSA_LE28_GE3 | Yes/no >=3 overlapping SA fills with <=7 days supply during a quarter |
| 464 | OVERLAP_SUP_LE7_QTR_GE2 | Yes/no >=2 fills with <=7 days supply that overlap with a preceding fill during a quarter |
| 465 | OVERLAP_SUP_LE7_QTR_GE3 | Yes/no >=3 fills with <=7 days supply that overlap with a preceding fill during a quarter |
| 466 | OVERLAP_SUP_LE7_QTR_GE5 | Yes/no >=5 fills with <=7 days supply that overlap with a preceding fill during a quarter |
| 467 | OVERLAP_SUP_LE28_QTR_GE3 | Yes/no >=3 fills with 8-28 days supply that overlap with a preceding fill during a quarter |
| 468 | OVERLAP_SUP_LE28_QTR_GE6 | Yes/no >=6 fills with 8-28 days supply that overlap with a preceding fill during a quarter |
| 469 | OVERLAP_SUP_LE28_QTR_GE8 | Yes/no >=8 fills with 8-28 days supply that overlap with a preceding fill during a quarter |
| 470 | OVERLAP_SUP_GT28_QTR_GE5 | Yes/no >=5 fills with >28 days supply that overlap with a preceding fill during a quarter |
| 471 | OVERLAP_PCT_SUP_GE25_GE6 | Yes/no >=6 fills that overlap by >=25% with a preceding fill during a quarter |
| 472 | OVERLAP_PCT_SUP_GE25_GE9 | Yes/no >=9 fills that overlap by >=25% with a preceding fill during a quarter |
| 473 | OVERLAP_PCT_SUP_GE50_GE4 | Yes/no >=4 fills that overlap by >=50% with a preceding fill during a quarter |
| 474 | OVERLAP_PCT_SUP_GE50_GE6 | Yes/no >=6 fills that overlap by >=50% with a preceding fill during a quarter |
| 475 | OVERLAP_GE2_COUNT_GE8 | Yes/no >=8 fills (ER or SA) that overlap by >=2 days with a preceding fill during a quarter |
| 476 | OVERLAP_GE2_COUNT_GE11 | Yes/no >=11 fills (ER or SA) that overlap by >=2 days with a preceding fill during a quarter |
| 477 | OVERLAP_SUP2_LE7_QTR_GE1 | Ever/never >=2 fills with <=7 days supply overlap during a quarter |
| 478 | OVERLAP_SUP2_LE28_QTR_GE5 | Yes/no >=5 fills with 8-28 days supply overlap during a quarter |
| 479 | OVERLAP_SUP2_LE28_QTR_GE7 | Yes/no >=7 fills with 8-28 days supply overlap during a quarter |
| 480 | OVERLAP_SUP2_GT28_QTR_GE4 | Yes/no >=4 fills with >28 days supply overlap during a quarter |
| 481 | EARLY_FILL_NDC_COUNT_GE5 | Yes/no >=5 fills with the same NDC code overlap during the 18 months around index date |
| 482 | EARLY_FILL_NDC_COUNT_GE8 | Yes/no >=8 fills with the same NDC code overlap during the 18 months around index date |
| 483 | EARLY_FILL_NDC_DAYS_SUM_GE24 | Yes/no >=24 days supply of overlap for a single NDC code during the 6 months before and 18 months after index date |
| 484 | EARLY_FILL_NDC_DAYS_SUM_GE40 | Yes/no >=40 days supply of overlap for a single NDC code during the 6 months before and 18 months after index date |
| 485 | EARLY_FILL_NDC_DAYS_SUM_GE60 | Yes/no >=60 days supply of overlap for a single NDC code during the 6 months before and 18 months after index date |
| 486 | EARLY_FILL_NDC_DAYS_GE2_GE4 | Yes/no >=4 fills with the same NDC code overlap by >=2 days during the 18 months around index date |
| 487 | EARLY_FILL_NDC_DAYS_GE2_GE6 | Yes/no >=6 fills with the same NDC code overlap by >=2 days during the 18 months around index date |
| 488 | EARLY_FILL_NDC_DAYS_GE2_GE8 | Yes/no >=8 fills with the same NDC code overlap by >=2 days during the 18 months around index date |
| 489 | EARLY_FILL_QTR_COUNT_NDC_GE3 | Yes/no >=3 overlapping fills with the same NDC code during a quarter |
| 490 | EARLY_FILL_QTR_COUNT_NDC_GE4 | Yes/no >=4 overlapping fills with the same NDC code during a quarter |
| 491 | EARLY_FILL_QTR_DAYS_SUM_GE16 | Yes/no >=16 days supply of overlap for a single NDC code during a quarter |
| 492 | EARLY_FILL_QTR_DAYS_SUM_GE22 | Yes/no >=22 days supply of overlap for a single NDC code during a quarter |
| 493 | EARLY_FILL_QTR_DAYS_SUM_GE24 | Yes/no >=24 days supply of overlap for a single NDC code during a quarter |
| 494 | EARLY_FILL_QTR_DAYS_GE2_GE2 | Yes/no >=2 fills with the same NDC code overlap by >=2 days during a quarter |
| 495 | EARLY_FILL_QTR_DAYS_GE2_GE3 | Yes/no >=3 fills with the same NDC code overlap by >=2 days during a quarter |
| 496 | MEQ_PER_DAY_18MO_GE120 | Yes/no >=120 MEQ per day during 18 months around index date |
| 497 | MEQ_PER_DAY_18MO_GE140 | Yes/no >=140 MEQ per day during 18 months around index date |
| 498 | MEQ_PER_DAY_18MO_GE200 | Yes/no >=200 MEQ per day during 18 months around index date |
| 499 | MEQ_ER_PER_DAY_18MO_GE120 | Yes/no >=120 MEQ per day from ER Opioids during 18 months around index date |
| 500 | MEQ_ER_PER_DAY_18MO_GE180 | Yes/no >=180 MEQ per day from ER Opioids during 18 months around index date |
| 501 | MEQ_ER_PER_DAY_18MO_GE200 | Yes/no >=200 MEQ per day from ER Opioids during 18 months around index date |
| 502 | MEQ_SA_PER_DAY_18MO_GE60 | Yes/no >=60 MEQ per day from SA Opioids during 18 months around index date |
| 503 | MEQ_PER_DAY_SUPPLY_18MO_GE100 | Yes/no >=100 MEQ per day supply during 18 months around index date |
| 504 | MEQ_PER_DAY_SUPPLY_18MO_GE120 | Yes/no >=120 MEQ per day supply during 18 months around index date |
| 505 | MEQ_PER_DAY_SUPPLY_18MO_GE200 | Yes/no >=200 MEQ per day supply during 18 months around index date |
| 506 | MEQ_PER_DAY_SUPPLY_ER_18MO_GE100 | Yes/no >=100 MEQ per day supply during 18 months around index date |
| 507 | MEQ_PER_DAY_SUPPLY_ER_18MO_GE120 | Yes/no >=120 MEQ per day supply during 18 months around index date |
| 508 | MEQ_PER_DAY_SUPPLY_ER_18MO_GE200 | Yes/no >=200 MEQ per day supply during 18 months around index date |
| 509 | EXCESS_RXSUP_TOTAL_QTRS_GE6 | Yes/no >=6 quarters with >1.2 excess days supply during the quarter |
| 510 | PCT_CHANGE_MEQ_GE33_N_QTR_GE3 | Yes/no >=33% increase in MEQ between two consecutive quarters at least 3 times during 36 months |
| 511 | PCT_CHANGE_MEQ_GE50_N_QTR_GE2 | Yes/no >=50% increase in MEQ between two consecutive quarters at least 2 times during 36 months |
| 512 | PCT_CHANGE_MEQ_GE75_N_QTR_GE2 | Yes/no >=75% increase in MEQ between two consecutive quarters at least 2 times during 36 months |
| 513 | PCT_CHANGE_MEQ_GE100_N_QTR_GE2 | Yes/no >=100% increase in MEQ between two consecutive quarters at least 2 times during 36 months |
| 514 | PCT_CHANGE_MEQ_GE150_N_QTR_GE2 | Yes/no >=150% increase in MEQ between two consecutive quarters at least 2 times during 36 months |
| 515 | AVG_DAILY_MEQ_GE120_COUNT__GE2 | Yes/no >=2 quarters with Average Daily MEQ >=120 during the quarter |
| 516 | AVG_DAILY_MEQ_IN_QTR_GE140 | Yes/no Average Daily MEQ >=140 during a quarter |
| 517 | AVG_DAILY_MEQ_IN_QTR_GE200 | Yes/no Average Daily MEQ >=200 during a quarter |
| 518 | AVG_DAILY_MEQ_ERLA_IN_QTR_GE140 | Yes/no Average Daily MEQ from ER >=140 during a quarter |
| 519 | AVG_DAILY_MEQ_ERLA_IN_QTR_GE200 | Yes/no Average Daily MEQ from ER >=200 during a quarter |
| 520 | ER_RX_COUNT_ANY_IN_QTR_GE2 | Yes/no >=2 opioid fills (ER or SA) dispensed on the same date as an ER encounter during a quarter |
| 521 | ER_RX_COUNT_SA_IN_QTR_GE2 | Yes/no >=2 SA fills dispensed on the same date as an ER encounter during a quarter |
| 522 | TOTAL_QTRS_ER_RX_EVER_ERLA_GE1 | Ever/never >=1 quarter with an ER fill dispensed on the same date as an ER encounter |
| 523 | TOTAL_QTRS_ER_RX_EVER_SA_GE2 | Yes/no >=2 quarters with >=1 SA fill dispensed on the same date as an ER encounter |
| 524 | ER_RXSUP_ANY_IN_QTR_GE14 | Yes/no >=14 total days supply of opioids (ER or SA) dispensed on the same date(s) as an ER encounter during a quarter |
| 525 | ER_RX_SUP_LE3_GE1 | Ever/never >=1 opioid fill (ER or SA) with 1-3 days supply dispensed on the same date as an ER encounter |
| 526 | ER_RX_SUP_LE7_GE2 | Yes/no >=2 opioid fills (ER or SA) with 1-7 days supply dispensed on the same date as an ER encounter during a quarter |
| 527 | ER_RX_WKND_GE1 | Ever/never >=1 opioid fill (ER or SA) dispensed on a Saturday, Sunday or Monday on the same date as an ER encounter |
| 528 | ER_RX_WKND_SUP_LE3_GE1 | Ever/never >=1 opioid fill (ER or SA) with 1-3 days supply dispensed on a Saturday, Sunday or Monday on the same date as an ER encounter |
| 529 | ER_RX_WKND_SUP_LE7_GE1 | Ever/never >=1 opioid fill (ER or SA) with 1-7 days supply dispensed on a Saturday, Sunday or Monday on the same date as an ER encounter |
| 530 | ER_RX_COUNT_ANY_GE2 | Yes/no >=2 opioid fills (ER or SA) dispensed on the same date as an ER encounter anytime during 36 months |
| 531 | ER_RX_COUNT_SA_GE2 | Yes/no >=2 SA fills dispensed on the same date as an ER encounter anytime during 36 months |
| 532 | ER_RXSUP_ANY_GE6 | Yes/no >=6 total days supply of opioids (ER or SA) dispensed on the same date as an ER encounter anytime during 36 months |
| 533 | ER_RXSUP_ANY_GE7 | Yes/no >=7 total days supply of opioids (ER or SA) dispensed on the same date as an ER encounter anytime during 36 months |
| 534 | ANTIPSYCHOTIC_TOTAL_QUARTE_GE3 | Yes/no >=3 quarters with >=1 antipsychotic dispensing during the quarter |
| 535 | ANTID_TOTAL_QUARTERS_GE3 | Yes/no >=3 quarters with >=1 antidepressant medication during the quarter |
| 536 | ANTIANXIETY_TOTAL_QUARTERS_GE2 | Yes/no >=2 quarters with >=1 antianxiety medication during the quarter |
| 537 | BENZODIAZEPINE_TOTAL_QUART_GE3 | Yes/no >=3 quarters with >=1 benzodiazepine dispensing during the quarter |
| 538 | BENZODIAZEPINE_TOTAL_QUART_GE6 | Yes/no >=6 quarters with >=1 benzodiazepine dispensing during the quarter |
| 539 | MUSCLE_RELAXER_TOTAL_QUART_GE2 | Yes/no >=2 quarters with >=1 muscle relaxer during the quarter |
| 540 | MUSCLE_RELAXER_TOTAL_QUART_GE8 | Yes/no >=8 quarters with >=1 muscle relaxer during the quarter |
| 541 | HOMEOPATHIC_TOTAL_QUARTERS_GE3 | Yes/no >=3 quarters with >=1 homeopathic medication during the quarter |
| 542 | MULTI_DRUGS_TOTAL_QUARTERS_GE7 | Yes/no >=7 quarters with opioids and at least one other psychoactive medication during the quarter |
| 543 | OPIOID_AND_BENZO_TOTAL_QUA_GE5 | Yes/no >=5 quarters with >=1 opioid and >=1 benzodiazepine dispensed during the quarter |
| 544 | RXSUP_QTR_ANTID_GE120 | Yes/no >=120 days supply of antidepressant during a quarter |
| 545 | RXSUP_QTR_ANTID_GE180 | Yes/no >=180 days supply of antidepressant during a quarter |
| 546 | RXSUP_QTR_ANTIANXIETY_GE10 | Yes/no >=10 days supply of antianxiety medication during a quarter |
| 547 | RXSUP_QTR_ANTIPSYCHOTIC_GE7 | Yes/no >=7 days supply of antipsychotic medication during a quarter |
| 548 | RXSUP_QTR_ANTICONVULSANTS_GE30 | Yes/no >=30 days supply of anticonvulsant medication during a quarter |
| 549 | RXSUP_QTR_BENZODIAZEPINE_GE30 | Yes/no >=30 days supply of benzodiazepine during a quarter |
| 550 | RXSUP_QTR_BENZODIAZEPINE_GE90 | Yes/no >=90 days supply of benzodiazepine during a quarter |
| 551 | RXSUP_QTR_BENZODIAZEPINE_GE100 | Yes/no >=100 days supply of benzodiazepine during a quarter |
| 552 | RXSUP_QTR_HYPNOTIC_GE30 | Yes/no >=30 days supply of hypnotics during a quarter |
| 553 | RXSUP_QTR_MUSCLE_RELAXER_GE20 | Yes/no >=20 days supply of muscle-relaxer during a quarter |
| 554 | RXSUP_QTR_MUSCLE_RELAXER_GE40 | Yes/no >=40 days supply of muscle-relaxer during a quarter |
| 555 | RXSUP_QTR_MUSCLE_RELAXER_GE60 | Yes/no >=60 days supply of muscle-relaxer during a quarter |
| 556 | RXSUP_QTR_HOMEOPATHIC_GE10 | Yes/no >=10 days supply of homeopathic medication during a quarter |
| 557 | ER_DURING_BENZO_RX_COUNT_GE2 | Yes/no >=2 ER encounters during active possession of benzodiazepine |
| 558 | RXSUP_QTR_ADD_GE6 | Yes/no >=6 days supply of ADD medication during a quarter |
| 559 | RXSUP_QTR_ADD_GE11 | Yes/no >=11 days supply of ADD medication during a quarter |
| 560 | RXSUP_QTR_ANTIANXIETY_GE56 | Yes/no >=56 days supply of antianxiety medication during a quarter |
| 561 | RXSUP_QTR_ANTIPSYCHOTIC_GE12 | Yes/no >=12 days supply of antipsychotic medication during a quarter |
| 562 | COUNT_ANTID_WITH_OPIOIDS_GE4 | Yes/no >=4 antidepressant dispensings during a quarter with >=1 opioid dispensing (ER or SA) |
| 563 | COUNT_ANTID_WITH_OPIOIDS_GE6 | Yes/no >=6 antidepressant dispensings during a quarter with >=1 opioid dispensing (ER or SA) |
| 564 | COUNT_ANXIO_WITH_OPIOIDS_GE3 | Yes/no >=3 antianxiety dispensings during a quarter with >=1 opioid dispensing (ER or SA) |
| 565 | COUNT_BENZO_WITH_OPIOIDS_GE4 | Yes/no >=4 benzodiazepine dispensings and >=1 opioid dispensing (ER or SA) during a quarter |
| 566 | COUNT_HOMEO_WITH_OPIOIDS_GE4 | Yes/no >=4 homeopathic dispensings and >=1 opioid dispensing (ER or SA) during a quarter |
| 567 | RX_TREAT_AA_EVER_N_QTRS_GE6 | Yes/no >=4 quarters with drugs used to treat opioid abuse/addiction |
| 568 | RXSUP_TREAT_AA_GE84 | Yes/no >=84 days supply of drugs used to treat opioid abuse/addiction during a quarter |
| 569 | RXSUP_TREAT_AA_GE100 | Yes/no >=100 days supply of drugs used to treat opioid abuse/addiction during a quarter |
| 570 | DAYS_SUPPLY_ANY_B180_GE250 | Yes/no >=282 days supply of opioids (ER or SA) during 6 months prior to index date |
| 571 | DAYS_SUPPLY_ANY_B180_GE282 | Yes/no >=282 days supply of opioids (ER or SA) during 6 months prior to index date |
| 572 | DAYS_SUPPLY_IR_B180_GE120 | Yes/no >=120 days supply of SA opioids during 6 months prior to index date |
| 573 | DAYS_SUPPLY_IR_B180_GE190 | Yes/no >=190 days supply of SA opioids during 6 months prior to index date |
| 574 | MAX_COUNT_RXSUP_ER_LE7_QTR | [MAX_COUNT_RXSUP_ER_LE7_QTR] Maximum number of ER/LA dispensings during a quarter with 1-7 days supply during a quarter |
| 575 | MAX_COUNT_RXSUP_ER_8LE14_QTR | [MAX_COUNT_RXSUP_ER_8LE14_QTR] Maximum number of ER/LA dispensings during a quarter with 8-14 days supply during a quarter |
| 576 | MAX_COUNT_RXSUP_ER_LE14_QTR | [MAX_COUNT_RXSUP_ER_LE14_QTR] Maximum number of ER/LA dispensings during a quarter with 1-14 days supply during a quarter |
| 577 | MAX_PCNT_RXSUP_ER_LE14_QTR | [MAX_PCNT_RXSUP_ER_LE14_QTR] Maximum percentage of ER/LA dispensings during a quarter that have 1-14 days supply during a quarter |
| 578 | MAX_RXSUP_ANY_TOTAL_QTR | [MAX_RXSUP_ANY_TOTAL_QTR] Maximum days supply of opioids (ER/LA or SA/IR) during any quarter |
| 579 | MAX_RXSUP_ER_TOTAL_QTR | [MAX_RXSUP_ER_TOTAL_QTR] Maximum days supply of ER/LAopioids during any quarter |
| 580 | MAX_RXSUP_SA_TOTAL_QTR | [MAX_RXSUP_SA_TOTAL_QTR] Maximum days supply of SA/IR opioids during any quarter |
| 581 | MAX_PCT_CHANGE_RXSUP_BTW_QTR | [MAX_PCT_CHANGE_RXSUP_BTW_QTR] Maximum percent change in days supply between two consecutive quarters with >0 days supply |
| 582 | PCT_CHANGE_RXSUP_GE50_PCT | [PCT_CHANGE_RXSUP_GE50_PCT] Number of times days supply increases by 50% or more between consecutive quarters |
| 583 | PCT_CHANGE_RXSUP_GE80_PCT | [PCT_CHANGE_RXSUP_GE80_PCT] Number of times days supply increases by 80% or more between consecutive quarters |
| 584 | PCT_CHANGE_RXSUP_GE100_PCT | [PCT_CHANGE_RXSUP_GE100_PCT] Number of times days supply increases by 100% or more between consecutive quarters |
| 585 | PCT_CHANGE_RXSUP_GE150_PCT | [PCT_CHANGE_RXSUP_GE150_PCT] Number of times days supply increases by 150% or more between consecutive quarters |
| 586 | PCT_CHANGE_RXSUP_GE200_PCT | [PCT_CHANGE_RXSUP_GE200_PCT] Number of times days supply increases by 200% or more between consecutive quarters |
| 587 | COUNT_RXSUP_TOTAL_INCREASE | [COUNT_RXSUP_TOTAL_INCREASE] Number of times Days Supply increases between two consecutive quarters with >0 days supply |
| 588 | MAX_PCT_DAYS_COVERED_QTR | [MAX_PCT_DAYS_COVERED_QTR] Maximum number of days covered in any quarter (Total Days Supply in Quarter relative to Total Days in Quarter) |
| 589 | MAX_COUNT_RXSUP_ER_LE28_QTR | [MAX_COUNT_RXSUP_ER_LE28_QTR] Maximum number of ER/LA dispensings with <=28 days supply during any quarter |
| 590 | MAX_RXSUP_ER_60_90 | [MAX_RXSUP_ER_60_90] Yes/no received no more than 60 days supply of ER/LA during the study period |
| 591 | EXCESS_RXSUP_TOTAL_QTRS | [EXCESS_RXSUP_TOTAL_QTRS] Total number of quarters with either >=1.2 excess days supply of ER/LA opioids or >=1.2 excess days supply of SA/IR during the quarter |
| 592 | MAX_EXCESS_RXSUP_ER_QTR | [MAX_EXCESS_RXSUP_ER_QTR] Maximum amount of excess days supply of ER/LAopioids during a quarter |
| 593 | MAX_EXCESS_RXSUP_SA_QTR | [MAX_EXCESS_RXSUP_SA_QTR] Maximum amount of excess days supply of SA/IR opioids during a quarter |
| 594 | MAX_EXCESS_RXSUP_QTR | [MAX_EXCESS_RXSUP_QTR] Maximum amount of excess days supply of either ER/LAor SA/IR opioids during a quarter |
| 595 | EXCESS_RXSUP_QTR_EVER | [EXCESS_RXSUP_QTR_EVER] Ever/never >=1.2 excess days supply of opioids (ER/LA or SA/IR) during a quarter |
| 596 | MAX_EXCESS_RXSUP_ER_QTR_GE120 | [MAX_EXCESS_RXSUP_ER_QTR_GE120] Ever/never >=1.2 excess days supply of ER/LA opioids during a quarter |
| 597 | MAX_EXCESS_RXSUP_SA_QTR_GE120 | [MAX_EXCESS_RXSUP_SA_QTR_GE120] Ever/never >=1.2 excess days supply of SA/IR opioids during a quarter |
| 598 | MAX_EXCESS_RXSUP_ER_QTR_GE150 | [MAX_EXCESS_RXSUP_ER_QTR_GE150] Ever/never >=1.5 excess days supply of ER/LA opioids during a quarter |
| 599 | MAX_EXCESS_RXSUP_SA_QTR_GE150 | [MAX_EXCESS_RXSUP_SA_QTR_GE150] Ever/never >=1.5 excess days supply of SA/IR opioids during a quarter |
| 600 | MIN_RXSUP_ANY_TOTAL_QTR | [MIN_RXSUP_ANY_TOTAL_QTR] Minimum non-zero days supply (ER/LA or SA/IR) during a quarter |
| 601 | MIN_RXSUP_ER_TOTAL_QTR | [MIN_RXSUP_ER_TOTAL_QTR] Minimum non-zero days supply of ER during a quarter |
| 602 | MIN_RXSUP_SA_TOTAL_QTR | [MIN_RXSUP_SA_TOTAL_QTR] Minimum non-zero days supply of SA during a quarter |
| 603 | DIF_BTW_MIN_MAX_RXSUP_ANY | [DIF_BTW_MIN_MAX_RXSUP_ANY] Difference between minimum non-zero days supply (ER/LA or SA/IR) during a quarter and maximum days supply (ER/LA or SA/IR) during a quarter |
| 604 | DIF_BTW_MIN_MAX_RXSUP_ER | [DIF_BTW_MIN_MAX_RXSUP_ER] Difference between minimum non-zero days supply of ER/LA during a quarter and maximum days supply of ER/LA during a quarter |
| 605 | DIF_BTW_MIN_MAX_RXSUP_SA | [DIF_BTW_MIN_MAX_RXSUP_SA] Difference between minimum non-zero days supply of SA/IR during a quarter and maximum days supply of SA/IR during a quarter |
| 606 | SUM_QTRS_WITH_RXSUP_ANY | [SUM_QTRS_WITH_RXSUP_ANY] Total number of quarters with >=1 days supply of opioids (ER/LA or SA/IR) during the quarter |
| 607 | SUM_QTRS_TOTAL_GE120 | [SUM_QTRS_TOTAL_GE120] Total number of quarters with >=120 days supply of opioids (ER/LA or SA/IR) during the quarter |
| 608 | MAX_COUNT_RXSUP_LE3_WKND_ANY | [MAX_COUNT_RXSUP_LE3_WKND_ANY] Maximum number of opioid dispensings (ER/LA or SA/IR) with 1-3 days supply dispensed on Saturday, Sunday, or Monday during a quarter |
| 609 | MAX_COUNT_RXSUP_LE14_WKND_ER | [MAX_COUNT_RXSUP_LE14_WKND_ER] Maximum number of ER/LA dispensings with 1-14 days supply dispensed on Saturday, Sunday, or Monday during a quarter |
| 610 | MAX_COUNT_RXSUP_LE7_WKND_ER | [MAX_COUNT_RXSUP_LE7_WKND_ER] Maximum number of ER/LA dispensings with 1-7 days supply dispensed on Saturday, Sunday, or Monday during a quarter |
| 611 | MAX_COUNT_RXSUP_8LE14_WKND_ER | [MAX_COUNT_RXSUP_8LE14_WKND_ER] Maximum number of ER/LA dispensings with 8-14 days supply dispensed on Saturday, Sunday, or Monday during a quarter |
| 612 | MAX_COUNT_RXSUP_ANY_LE14_QTR | [MAX_COUNT_RXSUP_ANY_LE14_QTR] Maximum number of dispensings (ER/LA or SA/IR) with 1-14 days supply during a quarter |
| 613 | SUM_COUNT_RXSUP_LE3_WKND_ANY | [SUM_COUNT_RXSUP_LE3_WKND_ANY] Total number of opioid dispensings (ER/LA or SA/IR) with 1-3 days supply dispensed on Saturday, Sunday, or Monday during the study period |
| 614 | SUM_COUNT_RXSUP_LE14_WKND_ER | [SUM_COUNT_RXSUP_LE14_WKND_ER] Total number of ER/LA dispensings with 1-14 days supply dispensed on Saturday, Sunday, or Monday during the study period |
| 615 | SUM_COUNT_RXSUP_LE7_WKND_ER | [SUM_COUNT_RXSUP_LE7_WKND_ER] Total number of ER/LA dispensings with 1-7 days supply dispensed on Saturday, Sunday, or Monday during a quarter |
| 616 | SUM_COUNT_RXSUP_8LE14_WKND_ER | [SUM_COUNT_RXSUP_8LE14_WKND_ER] Total number of ER/LA dispensings with 8-14 days supply dispensed on Saturday, Sunday, or Monday during a quarter |
| 617 | SUM_COUNT_RXSUP_ANY_LE14 | [SUM_COUNT_RXSUP_ANY_LE14] Total number of dispensings (ER/LA or SA/IR) with 1-14 days supply during the study period |
| 618 | SUM_COUNT_RXSUP_ER_LE7 | [SUM_COUNT_RXSUP_ER_LE7] Total number of ER/LA dispensings with 1-7 days supply during the study period |
| 619 | SUM_COUNT_RXSUP_ER_GE8LE14 | [SUM_COUNT_RXSUP_ER_GE8LE14] Total number of ER/LA dispensings with 8-14 days supply during the study period |
| 620 | SUM_COUNT_RXSUP_ER_LE14 | [SUM_COUNT_RXSUP_ER_LE14] Total number of ER/LA dispensings with 1-14 days supply during the study period |
| 621 | SUM_COUNT_RXSUP_ER_LE28 | [SUM_COUNT_RXSUP_ER_LE28] Total number of ER/LA dispensings with 1-28 days supply during the study period |
| 622 | SUM_QTRS_ER_GE60 | SUM_QTRS_ER_GE60 |
| 623 | SUM_QTRS_SA_GE100 | SUM_QTRS_SA_GE100 |
| 624 | PERCENT_QTRS_SA_GE100 | PERCENT_QTRS_SA_GE100 |
| 625 | MAX_PCT_DAYS_COVERED_ER_QTR | MAX_PCT_DAYS_COVERED_ER_QTR |
| 626 | MAX_PCT_DAYS_COVERED_SA_QTR | MAX_PCT_DAYS_COVERED_SA_QTR |
| 627 | MAX_PCNT_RXSUP_ANY_LE14_QTR | MAX_PCNT_RXSUP_ANY_LE14_QTR |
| 628 | MAX_OVERLAP_PCT | [MAX_OVERLAP_PCT] Maximum percent of dispensings that overlap during any quarter |
| 629 | MAX_OVERLAP_COUNT_QTR | [MAX_OVERLAP_COUNT_QTR] Maximum number of overlapping dispensings (ER/LA or SA/IR) during any quarter |
| 630 | MAX_OVERLAP_DAYS_SUM_QTR | [MAX_OVERLAP_DAYS_SUM_QTR] Maximum number of cumulative excess days supply (ER/LA or SA/IR) during any quarter |
| 631 | MAX_OVERLAP_SUP_LE7_QTR | [MAX_OVERLAP_SUP_LE7_QTR] Maximum number of overlapping dispensings with 1-7 days supply during any quarter |
| 632 | MAX_OVERLAP_SUP_LE28_QTR | [MAX_OVERLAP_SUP_LE28_QTR] Maximum number of overlapping dispensings with 8-<28 days supply during any quarter |
| 633 | MAX_OVERLAP_SUP_GT28_QTR | [MAX_OVERLAP_SUP_GT28_QTR] Maximum number of overlapping dispensings with >28 days supply during any quarter |
| 634 | MAX_OVERLAP_SUP2_LE7_QTR | [MAX_OVERLAP_SUP2_LE7_QTR] Maximum number of overlapping dispensings in which BOTH have <=7 days supply during any quarter |
| 635 | MAX_OVERLAP_SUP2_LE28_QTR | [MAX_OVERLAP_SUP2_LE28_QTR] Maximum number of overlapping dispensings in which BOTH have 8-<28 days supply during any quarter |
| 636 | MAX_OVERLAP_SUP2_GT28_QTR | [MAX_OVERLAP_SUP2_GT28_QTR] Maximum number of overlapping dispensings in which BOTH have >28 days supply during any quarter |
| 637 | MAX_OVERLAP_PCT_SUP | [MAX_OVERLAP_PCT_SUP] Maximum percent of overlapping days supply between two dispensings (ER/LA or SA/IR) during a quarter (excluding those that overlap completely by dispensing date, type, and days s |
| 638 | MAX_OVERLAP_PCT_SUP_GE25 | [MAX_OVERLAP_PCT_SUP_GE25] Maximum number of dispensings (ER/LA or SA/IR) that overlap with a preceding dispensing by 25% or more days supply during a quarter |
| 639 | MAX_OVERLAP_PCT_SUP_GE50 | [MAX_OVERLAP_PCT_SUP_GE50] Maximum number of dispensings (ER/LA or SA/IR) that overlap with a preceding dispensing by 50% or more days supply during a quarter |
| 640 | MAX_OVERLAP_GE2_COUNT | [MAX_OVERLAP_GE2_COUNT] Maximum number of dispensings (ER/LA or SA/IR) that overlap by at least 2 days during any quarter |
| 641 | MAX_OVERLAP_SUP_LE3_WKND_QTR | [MAX_OVERLAP_SUP_LE3_WKND_QTR] Maximum number of dispensings (ER/LA or SA/IR) with 1-3 days supply that overlap with a preceding dispensing during a quarter |
| 642 | SUM_OVERLAP_SUP_LE3_WKND | [SUM_OVERLAP_SUP_LE3_WKND] Total number of dispensings (ER/LA or SA/IR) with 1-3 days supply that overlap with a preceding dispensing during the study period |
| 643 | MAX_OVERLAP_PCT_ER | [MAX_OVERLAP_PCT_ER] Maximum percent of ER/LA dispensings that overlap during a quarter |
| 644 | MAX_OVERLAP_COUNT_ER | [MAX_OVERLAP_COUNT_ER] Maximum number of ER/LA dispensings that overlap during a quarter |
| 645 | MAX_OVERLAP_DAYS_SUM_ER | [MAX_OVERLAP_DAYS_SUM_ER] Maximum amount of overlapping days supply of ER/LA during a quarter |
| 646 | MAX_OVERLAP_SUPER_LE7 | [MAX_OVERLAP_SUPER_LE7] Maximum number of ER/LA dispensings with <=7 days supply that overlap with a preceding dispensing (of any days supply) by 1 or more days during a quarter |
| 647 | MAX_OVERLAP_SUPER_LE28 | [MAX_OVERLAP_SUPER_LE28] Maximum number of ER/LA dispensings with 8-<28 days supply that overlap with a preceding dispensing (of any days supply) by 1 or more days during a quarter |
| 648 | MAX_OVERLAP_SUPER_GT28 | [MAX_OVERLAP_SUPER_GT28] Maximum number of ER/LA dispensings with >28 days supply that overlap with a preceding dispensing (of any days supply) by 1 or more days during a quarter |
| 649 | MAX_OVERLAP_PCT_SUPER | [MAX_OVERLAP_PCT_SUPER] Maximum percent of overlap between two ER/LA dispensings during a quarter (excluding those that overlap completely by dispensing date, type, and days supply) |
| 650 | MAX_OVERLAP_PCT_SUPER_GE25 | [MAX_OVERLAP_PCT_SUPER_GE25] Maximum number of ER/LA dispensings that overlap with an adjacent ER/LA dispensing by 25% or more days supply during a quarter |
| 651 | MAX_OVERLAP_PCT_SUPER_GE10 | [MAX_OVERLAP_PCT_SUPER_GE10] Maximum number of ER/LA dispensings that overlap with an adjacent ER/LA dispensing by 10% or more days supply during a quarter |
| 652 | MAX_OVERLAP_GE2_COUNT_ER | [MAX_OVERLAP_GE2_COUNT_ER] Maximum number of ER/LA dispensings that overlap by >=2 days with an adjacent ER/LA dispensing during any quarter |
| 653 | MAX_N_COMPLETE_OVERLAP_ER_SUP | [MAX_N_COMPLETE_OVERLAP_ER_SUP] Maximum number of completely overlapping ER/LA dispensings (by date, days supply) during a quarter |
| 654 | MAX_OVERLAP_SUP_LE14_ER_WKND | [MAX_OVERLAP_SUP_LE14_ER_WKND] Maximum number of ER/LA opioids with 1-14 days supply dispensed on Saturday, Sunday, or Monday that overlaps with an adjacent ER/LA dispensing (of any days supply) |
| 655 | MAX_OVERLAP_SUP_LE7_ER_WKND | [MAX_OVERLAP_SUP_LE7_ER_WKND] Maximum number of ER/LA opioids with 1-7 days supply dispensed on Saturday, Sunday, or Monday that overlaps with an adjacent ER/LA dispensing (of any days supply) |
| 656 | MAX_OVERLAP_SUP_8LE14_ER_WKND | [MAX_OVERLAP_SUP_GE8LE14_ER_WKND] Maximum number of ER/LA opioids with 8-14 days supply dispensed on Saturday, Sunday, or Monday that overlaps with an adjacent ER/LA dispensing (of any days supply) |
| 657 | MAX_OVERLAP_PCT_SA | [MAX_OVERLAP_PCT_SA] Maximum percent of SA/IR dispensings that overlap during a quarter |
| 658 | MAX_OVERLAP_COUNT_SA | [MAX_OVERLAP_COUNT_SA] Maximum number of SA/IR dispensings that overlap during a quarter |
| 659 | MAX_OVERLAP_DAYS_SUM_SA | [MAX_OVERLAP_DAYS_SUM_SA] Maximum amount of overlapping days supply of SA/IR during a quarter |
| 660 | MAX_OVERLAP_SUPSA_LE7 | [MAX_OVERLAP_SUPSA_LE7] Maximum number of SA/IR dispensings with <=7 days supply that overlap with a preceding dispensing (of any days supply) by 1 or more days during a quarter |
| 661 | MAX_OVERLAP_SUPSA_LE28 | [MAX_OVERLAP_SUPSA_LE28] Maximum number of SA/IR dispensings with 8-<28 days supply that overlap with a preceding dispensing (of any days supply) by 1 or more days during a quarter |
| 662 | MAX_OVERLAP_SUPSA_GT28 | [MAX_OVERLAP_SUPSA_GT28] Maximum number of SA/IR dispensings with >28 days supply that overlap with a preceding dispensing (of any days supply) by 1 or more days during a quarter |
| 663 | MAX_OVERLAP_PCT_SUPSA | [MAX_OVERLAP_PCT_SUPSA] Maximum percent of overlap between two SA/IR dispensings during a quarter (excluding those that overlap completely by dispensing date, type, and days supply) |
| 664 | MAX_OVERLAP_PCT_SUPSA_GE25 | [MAX_OVERLAP_PCT_SUPSA_GE25] Maximum number of SA/IR dispensings that overlap with an adjacent SA/IR dispensing by 25% or more days supply during a quarter |
| 665 | MAX_OVERLAP_PCT_SUPSA_GE10 | [MAX_OVERLAP_PCT_SUPSA_GE10] Maximum number of SA/IR dispensings that overlap with an adjacent SA/IR dispensing by 10% or more days supply during a quarter |
| 666 | MAX_OVERLAP_GE2_COUNT_SA | [MAX_OVERLAP_GE2_COUNT_SA] Maximum number of SA/IR dispensings that overlap by >=2 days with an adjacent SA/IR dispensing during any quarter |
| 667 | MAX_N_COMPLETE_OVERLAP_SA_SUP | [MAX_N_COMPLETE_OVERLAP_SA_SUP] Maximum number of completely overlapping SA/IR dispensings (by date, days supply) during a quarter |
| 668 | MAX_OVERLAP_SUP_LE14_SA_WKND | [MAX_OVERLAP_SUP_LE14_SA_WKND] Maximum number of SA/IR opioids with 1-14 days supply dispensed on Saturday, Sunday, or Monday that overlaps with an adjacent SA/IR dispensing (of any days supply) |
| 669 | MAX_OVERLAP_SUP_LE7_SA_WKND | [MAX_OVERLAP_SUP_LE7_SA_WKND] Maximum number of SA/IR opioids with 1-7 days supply dispensed on Saturday, Sunday, or Monday that overlaps with an adjacent SA/IR dispensing (of any days supply) |
| 670 | MAX_OVERLAP_SUP_8LE14_SA_WKND | [MAX_OVERLAP_SUP_GE8LE14_SA_WKND] Maximum number of SA/IR opioids with 8-14 days supply dispensed on Saturday, Sunday, or Monday that overlaps with an adjacent SA/IR dispensing (of any days supply) |
| 671 | MAX_EARLY_FILL_NDC_COUNT | [MAX_EARLY_FILL_NDC_COUNT] Maximum number of early dispensings for a single NDC code 6 months before Index Date and 12 months after |
| 672 | MAX_EARLY_FILL_NDC_DAYS_SUM | [MAX_EARLY_FILL_NDC_DAYS_SUM] Maximum amount of overlapping days supply for a single NDC code 6 months before Index Date and 12 months after |
| 673 | MAX_EARLY_FILL_NDC_DAYS_GE2 | [MAX_EARLY_FILL_NDC_DAYS_GE2] Maximum number of dispensings that are >=2 days early for a single NDC code 6 months before Index Date and 12 months after |
| 674 | MAX_EARLY_FILL_NDC_PCT_AVG | [MAX_EARLY_FILL_NDC_PCT_AVG] Maximum percent of dispensings that are early for a single NDC code 6 months before Index Date and 12 months after |
| 675 | MAX_EARLY_FILL_QTR_COUNT_NDC | [MAX_EARLY_FILL_QTR_COUNT_NDC] Maximum number of early dispensings for a single NDC code during a quarter |
| 676 | MAX_EARLY_FILL_QTR_DAYS_SUM | [MAX_EARLY_FILL_QTR_DAYS_SUM] Maximum amount of overlapping days supply for a single NDC code during a quarter |
| 677 | MAX_EARLY_FILL_QTR_DAYS_GE2 | [MAX_EARLY_FILL_QTR_DAYS_GE2] Maximum number of dispensings that are >=2 days early for a single NDC code during a quarter |
| 678 | MAX_EARLY_FILL_QTR_DAYS_PCT | [MAX_EARLY_FILL_QTR_DAYS_PCT] Percent of dispensings that are early for a single NDC code during a quarter |
| 679 | MEQ_PER_DAY_TOTAL_GT120_EVER | [MEQ_PER_DAY_TOTAL_GT120_EVER] Ever/never has at least one day with total Daily MEQ >120 (based on all opioids active on that day) |
| 680 | MEQ_PER_DAY_GT120_EVER | [MEQ_PER_DAY_GT120_EVER] Ever/never receives fill (ER/LA or SA/IR) with >120 avg daily MEQ during the study period |
| 681 | TOTAL_MEQ_ERLA | [TOTAL_MEQ_ERLA] Total amount of MEQ from ERL/LA opioids |
| 682 | TOTAL_MEQ_SA | [TOTAL_MEQ_SA] Total amount of MEQ from SA/IR opioids |
| 683 | TOTAL_MEQ | [TOTAL_MEQ] Total amount of MEQ from ER/LA and SA/IR opioids |
| 684 | METHADONE_EVER | [METHADONE_EVER] Ever/never receives Methadone during the study period |
| 685 | MEQ_PER_DAY_GT120_COUNT | [MEQ_PER_DAY_GT120_COUNT] Total number of fills (ER/LA or SA/IR) with >120 avg daily MEQ during the study period |
| 686 | TOTAL_MEQ_18MO | [TOTAL_MEQ_18MO] Total MEQ (ER/LA or SA/IR) during 6 months before and 18 months after index date |
| 687 | TOTAL_MEQ_ER_18MO | [TOTAL_MEQ_ER_18MO] Total MEQ from ER/LA opioids during 6 months before and 18 months after index date |
| 688 | TOTAL_MEQ_SA_18MO | [TOTAL_MEQ_SA_18MO] Total MEQ from SA/IR opioids during 6 months before and 18 months after index date |
| 689 | TOTAL_MEQ_PER_DAY_18MO | [TOTAL_MEQ_PER_DAY_18MO] Average MEQ per day (ER/LA or SA/IR) during 6 months before and 18 months after index date |
| 690 | TOTAL_MEQ_ER_PER_DAY_18MO | [TOTAL_MEQ_ER_PER_DAY_18MO] Average MEQ per day from ER/LA opioids during 6 months before and 18 months after index date |
| 691 | TOTAL_MEQ_SA_PER_DAY_18MO | [TOTAL_MEQ_SA_PER_DAY_18MO] Average MEQ per day from SA/IR opioids during 6 months before and 18 months after index date |
| 692 | TOTAL_MEQ_PER_DAY_SUPPLY_18MO | [TOTAL_MEQ_PER_DAY_SUPPLY_18MO] Average MEQ per day of supply (ER/LA or SA/IR) during 6 months before and 18 months after index date |
| 693 | TOTAL_MEQ_PER_DAY_SUPPLY_ER_18MO | [TOTAL_MEQ_PER_DAY_SUPPLY_ER_18MO] Average MEQ per day of supply from ER/LA opioids during 6 months before and 18 months after index date |
| 694 | TOTAL_MEQ_PER_DAY_SUPPLY_SA_18MO | [TOTAL_MEQ_PER_DAY_SUPPLY_SA_18MO] Average MEQ per day of supply from SA/IR opioids during 6 months before and 18 months after index date |
| 695 | AVG_DAILY_MEQ_GE120_COUNT_QTRS | [AVG_DAILY_MEQ_GE120_COUNT_QTRS] Total number of quarters with Average Daily MEQ >=120 during the quarter |
| 696 | AVG_DAILY_MEQ_IN_QTR_INCREASE | [AVG_DAILY_MEQ_IN_QTR_INCREASE] Number of times avg daily MEQ increases between quarters |
| 697 | MAX_AVG_DAILY_MEQ_IN_QTR | [MAX_AVG_DAILY_MEQ_IN_QTR] Maximum avg daily MEQ from ER/LA or SA/IR opioids in any quarter |
| 698 | MAX_AVG_DAILY_MEQ_ERLA_IN_QTR | [MAX_AVG_DAILY_MEQ_ERLA_IN_QTR] Maximum Average Daily MEQ from ER opioids in any quarter |
| 699 | MAX_AVG_DAILY_MEQ_SA_IN_QTR | [MAX_AVG_DAILY_MEQ_SA_IN_QTR] Maximum Average Daily MEQ from SA opioids in any quarter |
| 700 | MAX_MEQ_TOTAL_IN_QTR | [MAX_MEQ_TOTAL_IN_QTR] Maximum total MEQ from ER/LA or SA/IR opioids in any quarter |
| 701 | MAX_MEQ_ERLA_TOTAL_IN_QTR | [MAX_MEQ_ERLA_TOTAL_IN_QTR] Maximum total MEQ from ER opioids in any quarter |
| 702 | MAX_MEQ_SA_TOTAL_IN_QTR | [MAX_MEQ_SA_TOTAL_IN_QTR] Maximum total MEQ from SA opioids in any quarter |
| 703 | MAX_MEQ_GE120_COUNT_IN_QTR | [MAX_MEQ_GE120_COUNT_IN_QTR] Maximum number of fills (ER/LA or SA/IR) with >=120 avg daily MEQ in any quarter |
| 704 | MAX_MEQ_GE120_DAYS_IN_QTR | [MAX_MEQ_GE120_DAYS_IN_QTR] Maximum total days supply of fills (ER/LA or SA/IR) with >=120 avg daily MEQ in any quarter |
| 705 | PERCENT_QTRS_WITH_MEQ_GE120 | [PERCENT_QTRS_WITH_MEQ_GE120] Percent of quarters with any opioids (ER/LA or SA/IR) during which avg daily MEQ was >=120 |
| 706 | MAX_PCT_CHANGE_MEQ_IN_QTR | [MAX_PCT_CHANGE_MEQ_IN_QTR] Maxmimum percent change in avg daily MEQ (ER/LA or SA/IR) between two consecutive quarters |
| 707 | PCT_CHANGE_MEQ_GE33_N_QTR | [PCT_CHANGE_MEQ_GE33_N_QTR] Number of times avg daily MEQ increases by >=33% between two consecutive quarters |
| 708 | PCT_CHANGE_MEQ_GE50_N_QTR | [PCT_CHANGE_MEQ_GE50_N_QTR] Number of times avg daily MEQ increases by >=50% between two consecutive quarters |
| 709 | PCT_CHANGE_MEQ_GE75_N_QTR | [PCT_CHANGE_MEQ_GE75_N_QTR] Number of times avg daily MEQ increases by >=75% between two consecutive quarters |
| 710 | PCT_CHANGE_MEQ_GE100_N_QTR | [PCT_CHANGE_MEQ_GE100_N_QTR] Number of times avg daily MEQ increases by >=100% between two consecutive quarters |
| 711 | PCT_CHANGE_MEQ_GE150_N_QTR | [PCT_CHANGE_MEQ_GE150_N_QTR] Number of times avg daily MEQ increases by >=150% between two consecutive quarters |
| 712 | MAX_MEQ_PER_DAY_SUPPLY_QTR | [MAX_MEQ_PER_DAY_SUPPLY_QTR] Maximum avg MEQ per day of supply (ER/LA or SA/IR) during a quarter |
| 713 | MAX_MEQ_PER_DAY_SUPPLY_ER_QTR | [MAX_MEQ_PER_DAY_SUPPLY_ER_QTR] Maximum avg MEQ per day of supply (ER only) during a quarter |
| 714 | MAX_MEQ_PER_DAY_SUPPLY_SA_QTR | [MAX_MEQ_PER_DAY_SUPPLY_SA_QTR] Maximum avg MEQ per day of supply (SA only) during a quarter |
| 715 | EVER_ER_GE40_SA_GT0_IN_QTR | [EVER_ER_GE40_SA_GT0_IN_QTR] Ever/never receives >=40 MEQ/day of supply from ER and >0 MEQ/day of supply from SA during a quarter |
| 716 | EVER_ER_GE60_SA_GT0_IN_QTR | [EVER_ER_GE60_SA_GT0_IN_QTR] Ever/never receives >=60 MEQ/day of supply from ER and >0 MEQ/day of supply from SA during a quarter |
| 717 | EVER_ER_GE120_SA_GT0_IN_QTR | [EVER_ER_GE120_SA_GT0_IN_QTR] Ever/never receives >=120 MEQ/day of supply from ER and >0 MEQ/day of supply from SA during a quarter |
| 718 | EVER_ER_GE40_SA_GE60_IN_QTR | [EVER_ER_GE40_SA_GE60_IN_QTR] Ever/never receives >=40 MEQ/day of supply from ER and >=60 MEQ/day of supply from SA during a quarter |
| 719 | EVER_ER_GE60_SA_GE60_IN_QTR | [EVER_ER_GE60_SA_GE60_IN_QTR] Ever/never receives >=60 MEQ/day of supply from ER and >=60 MEQ/day of supply from SA during a quarter |
| 720 | EVER_ER_GE120_SA_GE60_IN_QTR | [EVER_ER_GE120_SA_GE60_IN_QTR] Ever/never receives >=120 MEQ/day of supply from ER and >=60 MEQ/day of supply from SA during a quarter |
| 721 | EVER_ER_GE40_SA_GE80_IN_QTR | [EVER_ER_GE40_SA_GE80_IN_QTR] Ever/never receives >=40 MEQ/day of supply from ER and >=80 MEQ/day of supply from SA during a quarter |
| 722 | EVER_ER_GE60_SA_GE80_IN_QTR | [EVER_ER_GE60_SA_GE80_IN_QTR] Ever/never receives >=60 MEQ/day of supply from ER and >=80 MEQ/day of supply from SA during a quarter |
| 723 | EVER_ER_GE120_SA_GE80_IN_QTR | [EVER_ER_GE120_SA_GE80_IN_QTR] Ever/never receives >=120 MEQ/day of supply from ER and >=80 MEQ/day of supply from SA during a quarter |
| 724 | N_DistinctQtrANTIDRx | [N_DistinctQtrANTIDRx] Number of quarters with >=1 dispensing of Anti-Depressant Medications |
| 725 | Pct_QtrANTIDRx | [Pct_QtrANTIDRx] Number of quarters with >=1 dispensing of Anti-Depressant Medications relative to number of quarters with >=1 dispensing of any opioid (ER/LA or SA/IR) |
| 726 | MAX_RXSUP_QTR_ANTID | [MAX_RXSUP_QTR_ANTID] Maximum days supply of Anti-Depressant Medications in a quarter |
| 727 | ANTID_TOTAL_QUARTERS | [ANTID_TOTAL_QUARTERS] Total number of quarters with >=1 dispensing of Anti-Depressant Medications |
| 728 | ANTIANXIETY_TOTAL_QUARTERS | [ANTIANXIETY_TOTAL_QUARTERS] Total number of quarters with >=1 dispensing of Anti-Anxiety Medications |
| 729 | OPIOID_AND_ANXIO_GE1 | [OPIOID_AND_ANXIO_GE1] >=1 quarters with at least one opioid dispensing and at least one Anxiolytics dispensing during the quarter (fills do not need to overlap) |
| 730 | OPIOID_AND_ANXIO_GE3 | [OPIOID_AND_ANXIO_GE3] >=3 quarters with at least one opioid dispensing and at least one Anxiolytics dispensing during the quarter (fills do not need to overlap) |
| 731 | ADD_TOTAL_QUARTERS | [ADD_TOTAL_QUARTERS] Total number of quarters with >=1 dispensing of ADD Medications |
| 732 | OPIOID_AND_ADD_GE1 | [OPIOID_AND_ADD_GE1] >=1 quarters with at least one opioid dispensing and at least one ADD dispensing during the quarter (fills do not need to overlap) |
| 733 | OPIOID_AND_ADD_GE3 | [OPIOID_AND_ADD_GE3] >=3 quarters with at least one opioid dispensing and at least one ADD dispensing during the quarter (fills do not need to overlap) |
| 734 | ANTIPSYCHOTIC_TOTAL_QUARTERS | [ANTIPSYCHOTIC_TOTAL_QUARTERS] Total number of quarters with >=1 dispensing of Anti-Psychotic Medications |
| 735 | ANTICONVULSANTS_TOTAL_QUARTERS | [ANTICONVULSANTS_TOTAL_QUARTERS] Total number of quarters with >=1 dispensing of anticonvulsant medication |
| 736 | BARBITURATE_TOTAL_QUARTERS | [BARBITURATE_TOTAL_QUARTERS] Total number of quarters with >=1 dispensing of barbiturate medication |
| 737 | BENZODIAZEPINE_TOTAL_QUARTERS | [BENZODIAZEPINE_TOTAL_QUARTERS] Total number of quarters with >=1 dispensing of benzodiazepine medication |
| 738 | HYPNOTIC_TOTAL_QUARTERS | [HYPNOTIC_TOTAL_QUARTERS] Total number of quarters with >=1 dispensing of hypnotic medication |
| 739 | LITHIUM_TOTAL_QUARTERS | [LITHIUM_TOTAL_QUARTERS] Total number of quarters with >=1 dispensing of lithium medication |
| 740 | MUSCLE_RELAXER_TOTAL_QUARTERS | [MUSCLE_RELAXER_TOTAL_QUARTERS] Total number of quarters with >=1 dispensing of muscle relaxer medication |
| 741 | HOMEOPATHIC_TOTAL_QUARTERS | [HOMEOPATHIC_TOTAL_QUARTERS] Total number of quarters with >=1 dispensing of homeopathic medication |
| 742 | STIMULANTS_TOTAL_QUARTERS | [STIMULANTS_TOTAL_QUARTERS] Total number of quarters with >=1 dispensing of stimulant medication |
| 743 | OPIOID_AND_ANTIPSYCHOTIC_GE1 | [OPIOID_AND_ANTIPSYCHOTIC_GE1] Yes/no whether has received >=1 dispensing of any opioid (ER/LA or SA/IR) AND >=1 dispensing of Anti-Psychotic Medications for >=1 quarters during the study period |
| 744 | OPIOID_AND_ANTIPSYCHOTIC_GE3 | [OPIOID_AND_ANTIPSYCHOTIC_GE3] Yes/no whether has received >=1 dispensing of any opioid (ER/LA or SA/IR) AND >=1 dispensing of Anti-Psychotic Medications for >=3 quarters during the study period |
| 745 | OPIOID_AND_BENZO_TOTAL_QUARTERS | [OPIOID_AND_BENZO_TOTAL_QUARTERS] Total number of quarters with >=1 opioid dispensing and >=1 Benzodiazepine dispensing during the quarter (fills do not need to overlap) |
| 746 | OPIOID_AND_BENZO_GE1 | [OPIOID_AND_BENZO_GE1] >=1 quarters with at least one opioid dispensing and at least one Benzodiazepine dispensing during the quarter (fills do not need to overlap) |
| 747 | OPIOID_AND_BENZO_GE3 | [OPIOID_AND_BENZO_GE3] >=3 quarters with at least one opioid dispensing and at least one Benzodiazepine dispensing during the quarter (fills do not need to overlap) |
| 748 | OPIOID_AND_HYPNO_TOTAL_QUARTERS | [OPIOID_AND_HYPNO_TOTAL_QUARTERS] Total number of quarters with >=1 opioid dispensing and >=1 Hypnotics dispensing during the quarter (fills do not need to overlap) |
| 749 | OPIOID_AND_HYPNO_GE1 | [OPIOID_AND_HYPNO_GE1] >=1 quarters with at least one opioid dispensing and at least one Hypnotics dispensing during the quarter (fills do not need to overlap) |
| 750 | OPIOID_AND_HYPNO_GE3 | [OPIOID_AND_HYPNO_GE3] >=3 quarters with at least one opioid dispensing and at least one Hypnotics dispensing during the quarter (fills do not need to overlap) |
| 751 | MAX_N_CONCURRENT_DRUGS_IN_QTR | [MAX_N_CONCURRENT_DRUGS_IN_QTR] Maximum number of drug categories received during a quarter |
| 752 | MULTI_DRUGS_TOTAL_QUARTERS | [MULTI_DRUGS_TOTAL_QUARTERS] Total number of quarters with >=1 opioid dispensing and >=1 psychoactive medication dispensing during the quarter |
| 753 | MAX_COUNT_ANTID_WITH_OPIOIDS | [MAX_COUNT_ANTID_WITH_OPIOIDS] Maximum number of antidepressant dispensings during a quarter during which opioids were also dispensed |
| 754 | MAX_COUNT_ADD_WITH_OPIOIDS | [MAX_COUNT_ADD_WITH_OPIOIDS] Maximum number of ADD dispensings during a quarter during which opioids were also dispensed |
| 755 | MAX_COUNT_ANXIO_WITH_OPIOIDS | [MAX_COUNT_ANXIO_WITH_OPIOIDS] Maximum number of antianxiety dispensings during a quarter during which opioids were also dispensed |
| 756 | MAX_COUNT_ANTIP_WITH_OPIOIDS | [MAX_COUNT_ANTIP_WITH_OPIOIDS] Maximum number of antipsychotic dispensings during a quarter during which opioids were also dispensed |
| 757 | MAX_COUNT_ANTIC_WITH_OPIOIDS | [MAX_COUNT_ANTIC_WITH_OPIOIDS] Maximum number of anticonvulsant dispensings during a quarter during which opioids were also dispensed |
| 758 | MAX_COUNT_BARBI_WITH_OPIOIDS | [MAX_COUNT_BARBI_WITH_OPIOIDS] Maximum number of barbiturate dispensings during a quarter during which opioids were also dispensed |
| 759 | MAX_COUNT_BENZO_WITH_OPIOIDS | [MAX_COUNT_BENZO_WITH_OPIOIDS] Maximum number of benzodiazepine dispensings during a quarter during which opioids were also dispensed |
| 760 | MAX_COUNT_HYPNO_WITH_OPIOIDS | [MAX_COUNT_HYPNO_WITH_OPIOIDS] Maximum number of hypnotic dispensings during a quarter during which opioids were also dispensed |
| 761 | MAX_COUNT_MUSCL_WITH_OPIOIDS | [MAX_COUNT_MUSCL_WITH_OPIOIDS] Maximum number of muscle relaxer dispensings during a quarter during which opioids were also dispensed |
| 762 | MAX_COUNT_HOMEO_WITH_OPIOIDS | [MAX_COUNT_HOMEO_WITH_OPIOIDS] Maximum number of homeopathic dispensings during a quarter during which opioids were also dispensed |
| 763 | MAX_COUNT_STIMU_WITH_OPIOIDS | [MAX_COUNT_STIMU_WITH_OPIOIDS] Maximum number of stimulant dispensings during a quarter during which opioids were also dispensed |
| 764 | N_DistinctQtrADDRx | [N_DistinctQtrADDRx] Number of quarters with >=1 dispensing of Attention Deficit Disorder Medications |
| 765 | Pct_QtrADDRx | [Pct_QtrADDRx] Number of quarters with >=1 dispensing of Attention Deficit Disorder Medications relative to number of quarters with >=1 dispensing of any opioid (ER/LA or SA/IR) |
| 766 | MAX_RXSUP_QTR_ADD | [MAX_RXSUP_QTR_ADD] Maximum days supply of Attention Deficit Disorder Medications in a quarter during the study period |
| 767 | N_DistinctQtrANTIANXIETYRx | [N_DistinctQtrANTIANXIETYRx] Number of quarters with >=1 dispensing of Anti-Anxiety Medications |
| 768 | Pct_QtrANTIANXIETYRx | [PCT_QTRANTIANXIETYRX] Number of quarters with >=1 Anti-Anxiety Medication dispensing compared to number of quarters with >=1 opioid dispensing (ER/LA or SA/IR) |
| 769 | MAX_RXSUP_QTR_ANTIANXIETY | [MAX_RXSUP_QTR_ANTIANXIETY] Maximum days supply of Anti-Anxiety Medications in a quarter during the study period |
| 770 | N_DistinctQtrANTIPSYCHOTICRx | [N_DistinctQtrANTIPSYCHOTICRx] Number of quarters with >=1 dispensing of Anti-Psychotic Medications |
| 771 | Pct_QtrANTIPSYCHOTICRx | [Pct_QtrANTIPSYCHOTICRx] Number of quarters with >=1 dispensing of Anti-Psychotic Medications relative to number of quarters with >=1 dispensing of any opioid (ER/LA or SA/IR) |
| 772 | MAX_RXSUP_QTR_ANTIPSYCHOTIC | [MAX_RXSUP_QTR_ANTIPSYCHOTIC] Maximum days supply of Anti-Psychotic Medications in a quarter during the study period |
| 773 | N_DistinctQtrANTICONVULSANTSRx | [N_DistinctQtrANTICONVULSANTSRx] Number of quarters with >=1 dispensing of Anti-Convulsant Medications |
| 774 | Pct_QtrANTICONVULSANTSRx | [Pct_QtrANTICONVULSANTSRx] Number of quarters with >=1 dispensing of Anti-Convulsant Medications relative to number of quarters with >=1 dispensing of any opioid (ER/LA or SA/IR) |
| 775 | MAX_RXSUP_QTR_ANTICONVULSANTS | [MAX_RXSUP_QTR_ANTICONVULSANTS] Maximum days supply of Anti-Convulsant Medications in a quarter during the study period |
| 776 | N_DistinctQtrBARBITURATERx | [N_DistinctQtrBARBITURATERx] Number of quarters with >=1 dispensing of Barbiturates Medications |
| 777 | Pct_QtrBARBITURATERx | [Pct_QtrBARBITURATERx] Number of quarters with >=1 dispensing of Barbiturates Medications relative to number of quarters with >=1 dispensing of any opioid (ER/LA or SA/IR) |
| 778 | MAX_RXSUP_QTR_BARBITURATE | [MAX_RXSUP_QTR_BARBITURATE] Maximum days supply of Barbiturates Medications in a quarter during the study period |
| 779 | N_DistinctQtrBENZODIAZEPINERx | [N_DistinctQtrBENZODIAZEPINERx] Number of quarters with >=1 dispensing of Benzodiazepine Medications |
| 780 | Pct_QtrBENZODIAZEPINERx | [Pct_QtrBENZODIAZEPINERx] Number of quarters with >=1 dispensing of Benzodiazepine Medications relative to number of quarters with >=1 dispensing of any opioid (ER/LA or SA/IR) |
| 781 | MAX_RXSUP_QTR_BENZODIAZEPINE | [MAX_RXSUP_QTR_BENZODIAZEPINE] Maximum days supply of Benzodiazepine Medications in a quarter during the study period |
| 782 | N_DistinctQtrHYPNOTICRx | [N_DistinctQtrHYPNOTICRx] Number of quarters with >=1 dispensing of Hypnotic and other Sleeping Aid Medications |
| 783 | Pct_QtrHYPNOTICRx | [Pct_QtrHYPNOTICRx] Number of quarters with >=1 dispensing of Hypnotic and other Sleeping Aid Medications relative to number of quarters with >=1 dispensing of any opioid (ER/LA or SA/IR) |
| 784 | MAX_RXSUP_QTR_HYPNOTIC | [MAX_RXSUP_QTR_HYPNOTIC] Maximum days supply of Hypnotic and other Sleeping Aid Medications in a quarter during the study period |
| 785 | N_DistinctQtrLITHIUMRx | [N_DistinctQtrLITHIUMRx] Number of quarters with >=1 dispensing of Lithium Medications |
| 786 | Pct_QtrLITHIUMRx | [Pct_QtrLITHIUMRx] Number of quarters with >=1 dispensing of Lithium Medications relative to number of quarters with >=1 dispensing of any opioid (ER/LA or SA/IR) |
| 787 | MAX_RXSUP_QTR_LITHIUM | [MAX_RXSUP_QTR_LITHIUM] Maximum days supply of Lithium Medications in a quarter during the study period |
| 788 | N_DistinctQtrMUSCLE_RELAXERRx | [N_DistinctQtrMUSCLE_RELAXERRx] Number of quarters with >=1 dispensing of Muscle Relaxers Medications |
| 789 | Pct_QtrMUSCLE_RELAXERRx | [Pct_QtrMUSCLE_RELAXERRx] Number of quarters with >=1 dispensing of Muscle Relaxers Medications relative to number of quarters with >=1 dispensing of any opioid (ER/LA or SA/IR) |
| 790 | MAX_RXSUP_QTR_MUSCLE_RELAXER | [MAX_RXSUP_QTR_MUSCLE_RELAXER] Maximum days supply of Muscle Relaxers Medications in a quarter during the study period |
| 791 | N_DistinctQtrHOMEOPATHICRx | [N_DistinctQtrHOMEOPATHICRx] Number of quarters with >=1 dispensing of Homeopathic Medications |
| 792 | Pct_QtrHOMEOPATHICRx | [Pct_QtrHOMEOPATHICRx] Number of quarters with >=1 dispensing of Homeopathic Medications relative to number of quarters with >=1 dispensing of any opioid (ER/LA or SA/IR) |
| 793 | MAX_RXSUP_QTR_HOMEOPATHIC | [MAX_RXSUP_QTR_HOMEOPATHIC] Maximum days supply of Homeopathic Medications in a quarter during the study period |
| 794 | N_DistinctQtrSTIMULANTSRx | [N_DistinctQtrSTIMULANTSRx] Number of quarters with >=1 dispensing of Stimulants Medications |
| 795 | Pct_QtrSTIMULANTSRx | [Pct_QtrSTIMULANTSRx] Number of quarters with >=1 dispensing of Stimulants Medications relative to number of quarters with >=1 dispensing of any opioid (ER/LA or SA/IR) |
| 796 | MAX_RXSUP_QTR_STIMULANTS | [MAX_RXSUP_QTR_STIMULANTS] Maximum days supply of Stimulants Medications in a quarter during the study period |
| 797 | OPIOID_EVER_TOTAL_QUARTERS | OPIOID_EVER_TOTAL_QUARTERS |
| 798 | NON_OPIOID_EVER_TOTAL_QUARTERS | NON_OPIOID_EVER_TOTAL_QUARTERS |
| 799 | MULTI_DRUGS_EVER | MULTI_DRUGS_EVER |
| 800 | OPIOID_AND_ANTID_GE1 | OPIOID_AND_ANTID_GE1 |
| 801 | OPIOID_AND_ANTIANXIETY_GE1 | OPIOID_AND_ANTIANXIETY_GE1 |
| 802 | OPIOID_AND_ADD_TOTAL_QUARTERS | [OPIOID_AND_ADD_TOTAL_QUARTERS] Total number of quarters with >=1 opioid dispensing and >=1 ADD dispensing during the quarter (fills do not need to overlap) |
| 803 | OPIOID_AND_ANXIO_TOTAL_QUARTERS | [OPIOID_AND_ANXIO_TOTAL_QUARTERS] Total number of quarters with >=1 opioid dispensing and >=1 Anxiolytics dispensing during the quarter (fills do not need to overlap) |
| 804 | OPIOID_AND_ANTIPSYCHOTIC_QTRS | OPIOID_AND_ANTIPSYCHOTIC_QTRS |
| 805 | ANTID_EVER | [ANTID_EVER] Ever/never use of Anti-Depressant Medication |
| 806 | ADD_EVER | [ADD_EVER] Ever/never use of ADD Medication |
| 807 | ANTIANXIETY_EVER | [ANTIANXIETY_EVER] Ever/never use of Anti-Anxiety Medication |
| 808 | ANTICONVULSANTS_EVER | [ANTICONVULSANTS_EVER] Ever/never use of Anti-Convulsant Medication |
| 809 | ANTIPSYCHOTIC_EVER | [ANTIPSYCHOTIC_EVER] Ever/never use of Anti-Psychotic Medication |
| 810 | BARBITURATE_EVER | [BARBITURATE_EVER] Ever/never use of Barbiturates |
| 811 | BENZODIAZEPINE_EVER | [BENZODIAZEPINE_EVER] Ever/never use of Benzodiazepines |
| 812 | HYPNOTIC_EVER | [HYPNOTIC_EVER] Ever/never use of Hypnotic/Sleeping Aid Medication |
| 813 | LITHIUM_EVER | [LITHIUM_EVER] Ever/never use of Lithium |
| 814 | MUSCLE_RELAXER_EVER | [MUSCLE_RELAXER_EVER] Ever/never use of Muscle Relaxers |
| 815 | HOMEOPATHIC_EVER | [HOMEOPATHIC_EVER] Ever/never use of Homeopathic Medication for Mental Illness |
| 816 | STIMULANTS_EVER | STIMULANTS_EVER |
| 817 | ANTID_COUNT | ANTID_COUNT |
| 818 | ER_RX_a_EPI_OPIOID_benzo_YN | [ER_RX_a_EPI_OPIOID_BENZO_YN] Ever/never had an emergency room encounter in close proximity to overlapping opioid-Benzodiazepine fills during study period |
| 819 | ER_RX_a_EPI_OPIOID_benzo_N | [ER_RX_a_EPI_OPIOID_BENZO_n] Number of emergency room encounters in close proximity to overlapping opioid-Benzodiazepine fills during study period |
| 820 | ER_RX_a_EPI_OPIOID_benzo1_MAX | [ER_RX_a_EPI_OPIOID_BENZO1_MAX] Maximum number of opioid fills that are implicated in an opioid-Benzodiazepine fill overlap in close proximity with an emergency room encounter |
| 821 | ER_RX_a_EPI_OPIOID_benzo2_MAX | [ER_RX_a_EPI_OPIOID_BENZO2_MAX] Maximum number of Benzodiazepine fills that are implicated in an opioid-Benzodiazepine fill overlap in close proximity with an emergency room encounter |
| 822 | ER_RX_b_EPI_OPIOID_benzo_YN | [ER_RX_b_EPI_OPIOID_BENZO_YN] Ever/never had an emergency room encounter DURING overlapping opioid-Benzodiazepine fills during study period |
| 823 | ER_RX_b_EPI_OPIOID_benzo_N | [ER_RX_b_EPI_OPIOID_BENZO_N] Number of emergency room encounters that occur DURING overlapping opioid-Benzodiazepine fills during study period |
| 824 | ER_RX_b_EPI_OPIOID_benzo1_MAX | [ER_RX_b_EPI_OPIOID_BENZO1_MAX] Maximum number of opioid fills that are implicated in an opioid-Benzodiazepine fill overlapping with an emergency room encounter |
| 825 | ER_RX_b_EPI_OPIOID_benzo2_MAX | [ER_RX_b_EPI_OPIOID_BENZO2_MAX] Maximum number of Benzodiazepine fills that are implicated in an opioid-Benzodiazepine fill overlapping with an emergency room encounter |
| 826 | ER_RX_a_EPI_OPIOID_hypno_YN | [ER_RX_a_EPI_OPIOID_HYPNO_YN] Ever/never had an emergency room encounter in close proximity to overlapping opioid-Hypnotics fills during study period |
| 827 | ER_RX_a_EPI_OPIOID_hypno_N | [ER_RX_a_EPI_OPIOID_HYPNO_n] Number of emergency room encounters in close proximity to overlapping opioid-Hypnotics fills during study period |
| 828 | ER_RX_a_EPI_OPIOID_hypno1_MAX | [ER_RX_a_EPI_OPIOID_HYPNO1_MAX] Maximum number of opioid fills that are implicated in an opioid-Hypnotics fill overlap in close proximity with an emergency room encounter |
| 829 | ER_RX_a_EPI_OPIOID_hypno2_MAX | [ER_RX_a_EPI_OPIOID_HYPNO2_MAX] Maximum number of Hypnotics fills that are implicated in an opioid-Hypnotics fill overlap in close proximity with an emergency room encounter |
| 830 | ER_RX_b_EPI_OPIOID_hypno_YN | [ER_RX_b_EPI_OPIOID_HYPNO_YN] Ever/never had an emergency room encounter DURING overlapping opioid-Hypnotics fills during study period |
| 831 | ER_RX_b_EPI_OPIOID_hypno_N | [ER_RX_b_EPI_OPIOID_HYPNO_N] Number of emergency room encounters that occur DURING overlapping opioid-Hypnotics fills during study period |
| 832 | ER_RX_b_EPI_OPIOID_hypno1_MAX | [ER_RX_b_EPI_OPIOID_HYPNO1_MAX] Maximum number of opioid fills that are implicated in an opioid-Hypnotics fill overlapping with an emergency room encounter |
| 833 | ER_RX_b_EPI_OPIOID_hypno2_MAX | [ER_RX_b_EPI_OPIOID_HYPNO2_MAX] Maximum number of Hypnotics fills that are implicated in an opioid-Hypnotics fill overlapping with an emergency room encounter |
| 834 | ER_RX_a_EPI_OPIOID_add_YN | [ER_RX_a_EPI_OPIOID_ADD_YN] Ever/never had an emergency room encounter in close proximity to overlapping opioid-ADD fills during study period |
| 835 | ER_RX_a_EPI_OPIOID_add_N | [ER_RX_a_EPI_OPIOID_ADD_n] Number of emergency room encounters in close proximity to overlapping opioid-ADD fills during study period |
| 836 | ER_RX_a_EPI_OPIOID_add1_MAX | [ER_RX_a_EPI_OPIOID_ADD1_MAX] Maximum number of opioid fills that are implicated in an opioid-ADD fill overlap in close proximity with an emergency room encounter |
| 837 | ER_RX_a_EPI_OPIOID_add2_MAX | [ER_RX_a_EPI_OPIOID_ADD2_MAX] Maximum number of ADD fills that are implicated in an opioid-ADD fill overlap in close proximity with an emergency room encounter |
| 838 | ER_RX_b_EPI_OPIOID_add_YN | [ER_RX_b_EPI_OPIOID_ADD_YN] Ever/never had an emergency room encounter DURING overlapping opioid-ADD fills during study period |
| 839 | ER_RX_b_EPI_OPIOID_add_N | [ER_RX_b_EPI_OPIOID_ADD_N] Number of emergency room encounters that occur DURING overlapping opioid-ADD fills during study period |
| 840 | ER_RX_b_EPI_OPIOID_add1_MAX | [ER_RX_b_EPI_OPIOID_ADD1_MAX] Maximum number of opioid fills that are implicated in an opioid-ADD fill overlapping with an emergency room encounter |
| 841 | ER_RX_b_EPI_OPIOID_add2_MAX | [ER_RX_b_EPI_OPIOID_ADD2_MAX] Maximum number of ADD fills that are implicated in an opioid-ADD fill overlapping with an emergency room encounter |
| 842 | ER_RX_a_EPI_OPIOID_anxio_YN | [ER_RX_a_EPI_OPIOID_ANXIO_YN] Ever/never had an emergency room encounter in close proximity to overlapping opioid-Anxiolytics fills during study period |
| 843 | ER_RX_a_EPI_OPIOID_anxio_N | [ER_RX_a_EPI_OPIOID_ANXIO_n] Number of emergency room encounters in close proximity to overlapping opioid-Anxiolytics fills during study period |
| 844 | ER_RX_a_EPI_OPIOID_anxio1_MAX | [ER_RX_a_EPI_OPIOID_ANXIO1_MAX] Maximum number of opioid fills that are implicated in an opioid-Anxiolytics fill overlap in close proximity with an emergency room encounter |
| 845 | ER_RX_a_EPI_OPIOID_anxio2_MAX | [ER_RX_a_EPI_OPIOID_ANXIO2_MAX] Maximum number of Anxiolytics fills that are implicated in an opioid-Anxiolytics fill overlap in close proximity with an emergency room encounter |
| 846 | ER_RX_b_EPI_OPIOID_anxio_YN | [ER_RX_b_EPI_OPIOID_ANXIO_YN] Ever/never had an emergency room encounter DURING overlapping opioid-Anxiolytics fills during study period |
| 847 | ER_RX_b_EPI_OPIOID_anxio_N | [ER_RX_b_EPI_OPIOID_ANXIO_N] Number of emergency room encounters that occur DURING overlapping opioid-Anxiolytics fills during study period |
| 848 | ER_RX_b_EPI_OPIOID_anxio1_MAX | [ER_RX_b_EPI_OPIOID_ANXIO1_MAX] Maximum number of opioid fills that are implicated in an opioid-Anxiolytics fill overlapping with an emergency room encounter |
| 849 | ER_RX_b_EPI_OPIOID_anxio2_MAX | [ER_RX_b_EPI_OPIOID_ANXIO2_MAX] Maximum number of Anxiolytics fills that are implicated in an opioid-Anxiolytics fill overlapping with an emergency room encounter |
| 850 | OPIOID_benzo_OVERLAP_EVER | [OPIOID_benzo_OVERLAP_EVER\t\t\t ] Ever/never overlap in opioid (ER/LA or SA/IR) dispensings and benzo dispensings anytime during the study period |
| 851 | OPIOID_benzo_OVERLAP_COUNT | [OPIOID_benzo_OVERLAP_COUNT\t\t\t] Number of overlapping opioid (ERLA and SA) dispensings and benzo dispensings during the study period |
| 852 | OPIOID_benzo_OVERLAP_TOTAL_QTRS | [OPIOID_benzo_OVERLAP_TOTAL_QTRS ] Total number of quarters with >=1 overlapping opioid and benzo dispensing |
| 853 | OPIOID_benzo_OVERLAP_COUNT_MAX | [OPIOID_benzo_OVERLAP_COUNT_MAX\t] Maximum number of overlapping opioid and benzo dispensings during any quarter |
| 854 | OPIOID_hypno_OVERLAP_EVER | [OPIOID_hypno_OVERLAP_EVER\t\t\t ] Ever/never overlap in opioid (ER/LA or SA/IR) dispensings and hypno dispensings anytime during the study period |
| 855 | OPIOID_hypno_OVERLAP_COUNT | [OPIOID_hypno_OVERLAP_COUNT\t\t\t] Number of overlapping opioid (ERLA and SA) dispensings and hypno dispensings during the study period |
| 856 | OPIOID_hypno_OVERLAP_TOTAL_QTRS | [OPIOID_hypno_OVERLAP_TOTAL_QTRS ] Total number of quarters with >=1 overlapping opioid and hypno dispensing |
| 857 | OPIOID_hypno_OVERLAP_COUNT_MAX | [OPIOID_hypno_OVERLAP_COUNT_MAX\t] Maximum number of overlapping opioid and hypno dispensings during any quarter |
| 858 | OPIOID_add_OVERLAP_EVER | [OPIOID_add_OVERLAP_EVER\t\t\t ] Ever/never overlap in opioid (ER/LA or SA/IR) dispensings and add dispensings anytime during the study period |
| 859 | OPIOID_add_OVERLAP_COUNT | [OPIOID_add_OVERLAP_COUNT\t\t\t] Number of overlapping opioid (ERLA and SA) dispensings and add dispensings during the study period |
| 860 | OPIOID_add_OVERLAP_TOTAL_QTRS | [OPIOID_add_OVERLAP_TOTAL_QTRS ] Total number of quarters with >=1 overlapping opioid and add dispensing |
| 861 | OPIOID_add_OVERLAP_COUNT_MAX | [OPIOID_add_OVERLAP_COUNT_MAX\t] Maximum number of overlapping opioid and add dispensings during any quarter |
| 862 | OPIOID_anxio_OVERLAP_EVER | [OPIOID_anxio_OVERLAP_EVER\t\t\t ] Ever/never overlap in opioid (ER/LA or SA/IR) dispensings and anxio dispensings anytime during the study period |
| 863 | OPIOID_anxio_OVERLAP_COUNT | [OPIOID_anxio_OVERLAP_COUNT\t\t\t] Number of overlapping opioid (ERLA and SA) dispensings and anxio dispensings during the study period |
| 864 | OPIOID_anxio_OVERLAP_TOTAL_QTRS | [OPIOID_anxio_OVERLAP_TOTAL_QTRS ] Total number of quarters with >=1 overlapping opioid and anxio dispensing |
| 865 | OPIOID_anxio_OVERLAP_COUNT_MAX | [OPIOID_anxio_OVERLAP_COUNT_MAX\t] Maximum number of overlapping opioid and anxio dispensings during any quarter |
| 866 | ER_DURING_benzo_RX | [ER_DURING_benzo_RX] Ever/never emergency room encounters during active possession of benzo medication (i.e., emergency room encounter occurs between dispensing date and run-out date) |
| 867 | ER_DURING_benzo_RX_COUNT | [ER_DURING_benzo_RX_COUNT] Number of emergency room encounters during active possession of benzo medication (i.e., emergency room encounter occurs between dispensing date and run-out date) |
| 868 | ER_DURING_hypno_RX | [ER_DURING_hypno_RX] Ever/never emergency room encounters during active possession of hypno medication (i.e., emergency room encounter occurs between dispensing date and run-out date) |
| 869 | ER_DURING_hypno_RX_COUNT | [ER_DURING_hypno_RX_COUNT] Number of emergency room encounters during active possession of hypno medication (i.e., emergency room encounter occurs between dispensing date and run-out date) |
| 870 | ER_DURING_add_RX | [ER_DURING_add_RX] Ever/never emergency room encounters during active possession of add medication (i.e., emergency room encounter occurs between dispensing date and run-out date) |
| 871 | ER_DURING_add_RX_COUNT | [ER_DURING_add_RX_COUNT] Number of emergency room encounters during active possession of add medication (i.e., emergency room encounter occurs between dispensing date and run-out date) |
| 872 | ER_DURING_anxio_RX | [ER_DURING_anxio_RX] Ever/never emergency room encounters during active possession of anxio medication (i.e., emergency room encounter occurs between dispensing date and run-out date) |
| 873 | ER_DURING_anxio_RX_COUNT | [ER_DURING_anxio_RX_COUNT] Number of emergency room encounters during active possession of anxio medication (i.e., emergency room encounter occurs between dispensing date and run-out date) |
| 874 | MAX_ER_RX_SUP_LE3 | Maximum number of opioid fills (ER or SA) with 1-3 days supply dispensed on the same date as an ER encounter during a quarter |
| 875 | MAX_ER_RX_SUP_LE7 | Maximum number of opioid fills (ER or SA) with 1-3 days supply dispensed on the same date as an ER encounter during a quarter |
| 876 | MAX_ER_RX_SUP_8LE14 | Maximum number of opioid fills (ER or SA) with 1-3 days supply dispensed on the same date as an ER encounter during a quarter |
| 877 | SUM_ER_RX_SUP_LE3 | Total number of opioid fills (ER or SA) with 1-3 days supply dispensed on the same date as an ER encounter (ever in 36 months) |
| 878 | SUM_ER_RX_SUP_LE7 | Total number of opioid fills (ER or SA) with 1-3 days supply dispensed on the same date as an ER encounter (ever in 36 months) |
| 879 | SUM_ER_RX_SUP_8LE14 | Total number of opioid fills (ER or SA) with 1-3 days supply dispensed on the same date as an ER encounter (ever in 36 months) |
| 880 | MAX_ER_RX_WKND | Maximum number of opioid fills (ER or SA) with any days supply dispensed on the same date as an ER encounter during a quarter |
| 881 | MAX_ER_RX_WKND_SUP_LE3 | Maximum number of opioid fills (ER or SA) with 1-3 days supply dispensed on the same date as an ER encounter during a quarter |
| 882 | MAX_ER_RX_WKND_SUP_LE7 | Maximum number of opioid fills (ER or SA) with 1-7 days supply dispensed on the same date as an ER encounter during a quarter |
| 883 | SUM_ER_RX_WKND | Total number of opioid fills (ER or SA) with any days supply dispensed on the same date as an ER encounter (ever in 36 months) |
| 884 | SUM_ER_RX_WKND_SUP_LE3 | Total number of opioid fills (ER or SA) with 1-3 days supply dispensed on the same date as an ER encounter (ever in 36 months) |
| 885 | SUM_ER_RX_WKND_SUP_LE7 | Total number of opioid fills (ER or SA) with 1-7 days supply dispensed on the same date as an ER encounter (ever in 36 months) |
| 886 | ER_RX_WKND_EVER | Ever/never opioid fill (ER or SA) on the same date as an ER encounter on a Saturday, Sunday, or Monday (ever in 36 months) |
| 887 | MAX_ER_RX_COUNT_ANY_IN_QTR | Maximum number of opioid dispensings (ERLA or SA) on the same date as an ER encounter during the study period |
| 888 | MAX_ER_RX_COUNT_ERLA_IN_QTR | Maximum number of ERLA opioid dispensings on the same date as an ER encounter during the study period |
| 889 | MAX_ER_RX_COUNT_SA_IN_QTR | Maximum number of SA opioid dispensings on the same date as an ER encounter during the study period |
| 890 | SUM_ER_RX_COUNT_ANY | Total number of opioid dispensings (ERLA or SA) on the same date as an ER encounter during the study period |
| 891 | SUM_ER_RX_COUNT_ERLA | Total number of ERLA opioid dispensings on the same date as an ER encounter during the study period |
| 892 | SUM_ER_RX_COUNT_SA | Total number of SA opioid dispensings on the same date as an ER encounter during the study period |
| 893 | TOTAL_QTRS_ER_RX_EVER_ANY | Total number of quarters with any opioid dispensings (ERLA or SA) on the same date as an ER encounter during the study period |
| 894 | TOTAL_QTRS_ER_RX_EVER_ERLA | Total number of quarters with any ERLA opioid dispensings on the same date as an ER encounter during the study period |
| 895 | TOTAL_QTRS_ER_RX_EVER_SA | Total number of quarters with any SA opioid dispensings on the same date as an ER encounter during the study period |
| 896 | MAX_ER_RXSUP_ANY_IN_QTR | Maximum days supply of any opioids (ERLA or SA) on the same date as an ER encounter during any quarter |
| 897 | MAX_ER_RXSUP_ERLA_IN_QTR | Maximum days supply of ERLA opioids on the same date as an ER encounter during any quarter |
| 898 | MAX_ER_RXSUP_SA_IN_QTR | Maximum days supply of SA opioids on the same date as an ER encounter during any quarter |
| 899 | SUM_ER_RXSUP_ANY | Total days supply of any opioids (ERLA or SA) dispensed on the same date as an ER encounter during any quarter |
| 900 | SUM_ER_RXSUP_ERLA | Total days supply of ERLA opioids dispensed on the same date as an ER encounter during any quarter |
| 901 | SUM_ER_RXSUP_SA | Total days supply of SA opioids dispensed on the same date as an ER encounter during any quarter |
| 902 | ER_RX_ANY | Yes/no ever received an opioid dispensing on the same date as an ER encounter |
| 903 | DX_pois_inj_op_1 | [E850.1] Ever/never during study period |
| 904 | DX_pois_inj_op_2 | [E850.2] Ever/never during study period |
| 905 | DX_pois_inj_op_3 | [E935.1] Ever/never during study period |
| 906 | DX_pois_inj_op_4 | [E935.2] Ever/never during study period |
| 907 | DX_pois_inj_op_ANY | Ever/never >=1 ICD9/10 Dx code for Accidental Poisoning by Opioids during the study period |
| 908 | DX_pois_oth_1 | [E850.3] Ever/never during study period |
| 909 | DX_pois_oth_2 | [E850.4] Ever/never during study period |
| 910 | DX_pois_oth_3 | [E850.6] Ever/never during study period |
| 911 | DX_pois_oth_4 | [E850.8] Ever/never during study period |
| 912 | DX_pois_oth_5 | [E850.9] Ever/never during study period |
| 913 | DX_pois_oth_ANY | Ever/never >=1 ICD9/10 Dx code for Accidental Poisoning by Other Drugs during the study period |
| 914 | DX_pois_barb_hyp_1 | [E852.8] Ever/never during study period |
| 915 | DX_pois_barb_hyp_2 | [E852.9] Ever/never during study period |
| 916 | DX_pois_barb_hyp_3 | [E853.2] Ever/never during study period |
| 917 | DX_pois_barb_hyp_ANY | Ever/never >=1 ICD9/10 Dx code for Accidental Poisoning by Barbiturates and Sedatives/Hypnotics during the study period |
| 918 | DX_pois_psych_1 | [E854.0] Ever/never during study period |
| 919 | DX_pois_psych_2 | [E854.2] Ever/never during study period |
| 920 | DX_pois_psych_ANY | Ever/never >=1 ICD9/10 Dx code for Adverse Effects from Psychoactive Drugs during the study period |
| 921 | DX_ae_oth_1 | [E858.8] Ever/never during study period |
| 922 | DX_ae_oth_2 | [E858.9] Ever/never during study period |
| 923 | DX_ae_oth_ANY | Ever/never >=1 ICD9/10 Dx code for Adverse Effects from Other Drugs during the study period |
| 924 | DX_endo_1 | [421.0] Ever/never during study period |
| 925 | DX_endo_2 | [421.9] Ever/never during study period |
| 926 | DX_endo_3 | [424.90] Ever/never during study period |
| 927 | DX_endo_4 | [424.91] Ever/never during study period |
| 928 | DX_endo_ANY | Ever/never >=1 ICD9/10 Dx code for Endocarditis during the study period |
| 929 | EVER_POISONING_INJURY_DRUGS |  |
| 930 | RX_TREAT_AA_EVER | [RX_TREAT_AA_EVER] Ever/never receipt of medication used to treat opioid abuse/addiction |
| 931 | RX_TREAT_AA_COUNT | [RX_TREAT_AA_COUNT] Total number of dispensings for drugs used to treat opioid abuse/addiction |
| 932 | TOTAL_RXSUP_TREAT_AA | [TOTAL_RXSUP_TREAT_AA] Total days supply of drugs used to treat opioid abuse/addiction |
| 933 | TOTAL_RXSUP_TREAT_AA_18MO | [TOTAL_RXSUP_TREAT_AA_18MO] Total days supply of drugs used to treat opioid abuse/addiction during 18 months around index date |
| 934 | RX_TREAT_AA_COUNT_18MO | [RX_TREAT_AA_COUNT_18MO] Total number of dispensings for drugs used to treat opioid abuse/addiction during 18 months around index date |
| 935 | RX_TREAT_AA_EVER_18MO | [RX_TREAT_AA_EVER_18MO] Ever/never >=1 dispensing for medication used to treat opioid abuse/addiction during 18 months around index date |
| 936 | PRE_INDEX_TOTAL_RXSUP_TREAT_AA | PRE_INDEX_TOTAL_RXSUP_TREAT_AA |
| 937 | PRE_INDEX_TREAT_AA_COUNT | PRE_INDEX_TREAT_AA_COUNT |
| 938 | PRE_INDEX_TREAT_AA_EVER | PRE_INDEX_TREAT_AA_EVER |
| 939 | POST_INDEX_TOTAL_RXSUP_TREAT_AA | POST_INDEX_TOTAL_RXSUP_TREAT_AA |
| 940 | POST_INDEX_RX_TREAT_AA_COUNT | POST_INDEX_RX_TREAT_AA_COUNT |
| 941 | POST_INDEX_RX_TREAT_AA_EVER | POST_INDEX_RX_TREAT_AA_EVER |
| 942 | RX_TREAT_AA_EVER_N_QTRS | [RX_TREAT_AA_EVER_N_QTRS] Total number of quarters with >=1 dispensing for medication(s) used to treat opioid abuse/addiction |
| 943 | MAX_RXSUP_TREAT_AA | [MAX_RXSUP_TREAT_AA] Maximum days supply of medication(s) used to treat opioid abuse/addiction during a quarter |
| 944 | PX_80100 | [PX_80100] Ever/never Urine Drug Screen, qualitative multiple drug class (CPT: 80100) |
| 945 | PX_80101 | [PX_80101] Ever/never Urine Drug Screen, qualitative single drug class (CPT: 80101) |
| 946 | PX_80102 | [PX_80102] Ever/never Urine Drug Screen confirmation, each procedure (CPT: 80102) |
| 947 | PX_80154 | [PX_80154] Ever/never Benzodiazepines (CPT: 80154) |
| 948 | PX_80299 | [PX_80299] Ever/never Quantitation of drug, not elsewhere specified (CPT: 80299) |
| 949 | PX_80300 | [PX_80300] Ever/never (CPT: 80300) |
| 950 | PX_83925 | [PX_83925] Ever/never Opiates, drug and metabolites, each procedure (CPT: 83925) |
| 951 | PX_99408 | [PX_99408] Ever/never Alcohol and/or substance (other than tobacco) abuse structured screening (CPT: 99408) |
| 952 | PX_102_154_299 | [PX_102_154_299] Ever/never at least one of the following CPT codes for Urine Drug Screen (CPT: 80102, 80154, 80299) |
| 953 | UDS_COUNT_IN_QUARTER_MAX | [UDS_COUNT_IN_QUARTER_MAX] Maximum number of codes associated with Urine Drug Screen in any quarter |
| 954 | UDS_EVER_TOTAL_QUARTERS | [UDS_EVER_TOTAL_QUARTERS] Total number of quarters with at least one code associated with Urine Drug Screen |
| 955 | UDS_COUNT_IN_QUARTER_MAX_GE5 | [UDS_COUNT_IN_QUARTER_MAX_GE5] Yes/no >=5 Urine Drug Screenings during a quarter |
| 956 | UDS_EVER_TOTAL_QUARTERS_GE2 | [UDS_EVER_TOTAL_QUARTERS_GE2] Yes/no >=2 quarters with at least one Urine Drug Screening during the quarter |
| 957 | UDS_COUNT_IN_QUARTER_MAX_LE2010 | [UDS_COUNT_IN_QUARTER_MAX_LE2010] Maximum number of codes associated with Urine Drug Screen in any quarter, 2006-2010 |
| 958 | UDS_COUNT_IN_QUARTER_MAX_GT2010 | [UDS_COUNT_IN_QUARTER_MAX_GT2010] Maximum number of codes associated with Urine Drug Screen in any quarter, 2011-2015 |
| 959 | UDS_EVER_TOTAL_QUARTERS_LE2010 | [UDS_EVER_TOTAL_QUARTERS_LE2010] Total number of quarters with at least one code associated with Urine Drug Screen, 2006-2010 |
| 960 | UDS_EVER_TOTAL_QUARTERS_GT2010 | [UDS_EVER_TOTAL_QUARTERS_GT2010] Total number of quarters with at least one code associated with Urine Drug Screen, 2011-2015 |
| 961 | UDS_TOTAL_COUNT_LE2010 | [UDS_TOTAL_COUNT_LE2010] Total number of Urine Drug Screens, 2006-2010 |
| 962 | UDS_TOTAL_COUNT_GT2010 | [UDS_TOTAL_COUNT_GT2010] Total number of Urine Drug Screens, 2011-2015 |
| 963 | UDS_EVER_LE2010 | [UDS_EVER_LE2010] Yes/no ever had Urine Drug Screen, 2006-2010 |
| 964 | UDS_EVER_GT2010 | [UDS_EVER_GT2010] Yes/no ever had Urine Drug Screen, 2011-2015 |
| 965 | UDS_EVER | [UDS_EVER] Yes/no ever had Urine Drug Screen during the study period |
| 966 | DAYS_SUPPLY_ANY_B180 | Total days supply of opioids (ER and SA) during 6 months prior to index date |
| 967 | DAYS_SUPPLY_IR_B180 | Total days supply of SA opioids during 6 months prior to index date |
| 968 | DAYS_SUPPLY_ER_B180 | Total days supply of ER opioids during 6 months prior to index date |
| 969 | OPIOID_IR_B180 | Yes/no >=1 SA opioid dispensing during 6 months prior to index date |
| 970 | FILL_OVERLAP_AND_UDS_IN_QTR | Ever/never have an early refill (ERLA or SA) and Urine Drug Screen in the same quarter |
| 971 | FILL_OVERLAP_AND_UDS_N_QTR | Number of quarters with >=1 early refill (ERLA or SA) and >=1 Urine Drug Screen during the quarter |
| 972 | FILL_OVERLAP_GE2_UDS_GE2_IN_QTR | Yes/no >=2 early refills (ER or SA) AND >=2 Urine Drug Screens in the same quarter |
| 973 | FILL_OVERLAP_GE3_UDS_GE3_IN_QTR | Yes/no >=3 early refills (ER or SA) AND >=3 Urine Drug Screens in the same quarter |
| 974 | FILL_OVERLAP_GE4_UDS_GE4_IN_QTR | Yes/no >=4 early refills (ER or SA) AND >=4 Urine Drug Screens in the same quarter |
| 975 | MAX_FILL_OVERLAP_COUNT_AND_UDS | Maximum number of overlapping fills (ER or SA) in the same quarter with >=1 Urine Drug Screen |
| 976 | MAX_FILL_OVERLAP_AND_UDS_COUNT | Maximum number of Urine Drug Screens in the same quarter with >=1 overlapping fill (ER or SA) |
| 977 | ER_GE3_ER_LE14SUP | Yes/no ER visit and >=3 ER Dispensings with <=14 Days Supply during a quarter |
| 978 | ER_GE120_ER_EXCESS_SUP | Yes/no ER visit and >=1.2 Excess Days Supply of ER during a quarter |
| 979 | ER_GE120_ER_EXCESS_SUP_SA_GE50 | Yes/no ER visit and >=1.2 Excess Days Supply of ER and >=50 Days Supply of SA during a quarter |
| 980 | ER_GE120_ER_EXCESS_SUP_OTHER_RX | Yes/no ER visit and >=1.2 Excess Days Supply of ER and Concomitant Use of Other Psychoactive Drug(s) during a quarter |
| 981 | ER_GE2_NDC_OVERLAP | Yes/no ER visit and >=2 Overlapping Fills with the Same NDC Code during a quarter |
| 982 | ER_GE2_NDC_OVERLAP_OTHER_RX | Yes/no ER visit and >=2 Overlapping Fills with the Same NDC Code and Concomitant Use of Other Psychoactive Drug(s) during a quarter |
| 983 | ER_GE55_MEQ_PER_DAY_18MO | Yes/no ER visit and >=55 Avg MEQ/day for 18 Months Around Index Date |
| 984 | ER_GE55_AVG_DAILY_MEQ | Yes/no ER visit and >=55 Avg MEQ/day during a quarter |
| 985 | ER_GE55_MEQ_PER_DAY_18MO_AGELE45 | Yes/no ER visit and >=55 Avg MEQ/day for 18 Months Around Index Date and Age <=45 Years |
| 986 | ER_GE55_AVG_DAILY_MEQ_AGELE45 | Yes/no ER visit and >=55 Avg MEQ/day during a quarter and Age <=45 Years |
| 987 | ER_GE120_ER_XSUP_SA_GE50_MIDAGE | Yes/no ER visit and >=1.2 Excess Days Supply of ER and >=50 Days Supply of SA during a quarter and Age <65 |
| 988 | MAX_N_OTHER_MEDS | Maximum number of unique medication categories from which a person is receiving medications during a quarter |
| 989 | MAX_N_OTHER_MEDS_AND_OPIOIDS | Maximum number of unique medication categories from which a person is receiving medications while also receiving opiodis during a quarter |
| 990 | AVG_DAILY_MEQ_ER_GE100_SA_GE50 | Yes/no average daily MEQ from ER >=100 and from SA >=50 during a quarter |
| 991 | AVG_DAILY_MEQ_ER_GE140_SA_GE50 | Yes/no average daily MEQ from ER >=140 and from SA >=50 during a quarter |
| 992 | AVG_DAILY_MEQ_ER_GE120_SA_GE70 | Yes/no average daily MEQ from ER >=120 and from SA >=70 during a quarter |
| 993 | ER_GE2_NDC_OVERLAP_OTH_RX_YOUNG | Yes/no >=2 overlapping fills with the same NDC code AND an ER encounter AND >=1 fill for any psychoactive medication during the same quarter |
| 994 | OTHER_MEDS_AND_OPIOIDS_GE4_ADD | Yes/no received opioids and psychoactive medications from >=4 different categories (including at least one ADD medication) during the same quarter |
| 995 | OTHER_MEDS_AND_OPIOIDS_GE4_ANXIO | Yes/no received opioids and psychoactive medications from >=4 different categories (including at least one anti-anxiety medication) during the same quarter |
| 996 | OTHER_MEDS_AND_OPIOIDS_GE4_BENZO | Yes/no received opioids and psychoactive medications from >=4 different categories (including at least one Benzodiazepine medication) during the same quarter |
| 997 | OTHER_MEDS_AND_OPIOIDS_GE4_HYPNO | Yes/no received opioids and psychoactive medications from >=4 different categories (including at least one hypnotic medication) during the same quarter |
| 998 | OTHER_MEDS_AND_OPIOIDS_GE4_MUSCL | Yes/no received opioids and psychoactive medications from >=4 different categories (including at least one muscle-relaxer medication) during the same quarter |
| 999 | OTHER_MEDS_AND_OPIOIDS_GE4_ANTID | Yes/no received opioids and psychoactive medications from >=4 different categories (including at least one anti-depressant medication) during the same quarter |
| 1000 | OTHER_MEDS_AND_OPIOIDS_GE4_STIMU | Yes/no received opioids and psychoactive medications from >=4 different categories (including at least one stimulant medication) during the same quarter |
| 1001 | OTHER_MEDS_AND_OPIOIDS_GE4_ANTIP | Yes/no received opioids and psychoactive medications from >=4 different categories (including at least one anti-psychotic medication) during the same quarter |
| 1002 | OTHER_MEDS_AND_OPIOIDS_GE4_ANTIC | Yes/no received opioids and psychoactive medications from >=4 different categories (including at least one anti-convulsant medication) during the same quarter |
| 1003 | OTHER_MEDS_AND_OPIOIDS_GE4_BARBI | Yes/no received opioids and psychoactive medications from >=4 different categories (including at least one barbiturate medication) during the same quarter |
| 1004 | MAX_N_OTHER_MEDS_AND_OPIOIDS_GE4 | Yes/no received opioids and psychoactive medications from >=4 different categories during the same quarter |
| 1005 | MAX_N_OTHER_MEDS_AND_OPIOIDS_GE5 | Yes/no received opioids and psychoactive medications from >=5 different categories during the same quarter |
| 1006 | ER_RX_ab_EPI_OPIOID_OTH_PSYCH | Yes/no ER encounter near or during overlap between opioid use and use of another psychoactive medication |
| 1007 | i_A2_COUNT_RXSUP_LE7_WKND_ER_GE | [COUNT_RXSUP_LE7_WKND_ER_GE1] by [Age 46-64] |
| 1008 | i_A3_COUNT_RXSUP_LE7_WKND_ER_GE | [COUNT_RXSUP_LE7_WKND_ER_GE1] by [Age >=65] |
| 1009 | i_A2_COUNT_RXSUP_8LE14_WKND_ER_ | [COUNT_RXSUP_8LE14_WKND_ER_GE3] by [Age 46-64] |
| 1010 | i_A3_COUNT_RXSUP_8LE14_WKND_ER_ | [COUNT_RXSUP_8LE14_WKND_ER_GE3] by [Age >=65] |
| 1011 | i_A2_OVERLAP_SUP_LE14_ER_WKND_G | [OVERLAP_SUP_LE14_ER_WKND_GE1] by [Age 46-64] |
| 1012 | i_A3_OVERLAP_SUP_LE14_ER_WKND_G | [OVERLAP_SUP_LE14_ER_WKND_GE1] by [Age >=65] |
| 1013 | i_A2_OVERLAP_SUP_8LE14_ER_WKND_ | [OVERLAP_SUP_8LE14_ER_WKND_GE1] by [Age 46-64] |
| 1014 | i_A3_OVERLAP_SUP_8LE14_ER_WKND_ | [OVERLAP_SUP_8LE14_ER_WKND_GE1] by [Age >=65] |
| 1015 | i_A2_OVERLAP_SUP_LE7_SA_WKND_GE | [OVERLAP_SUP_LE7_SA_WKND_GE2] by [Age 46-64] |
| 1016 | i_A3_OVERLAP_SUP_LE7_SA_WKND_GE | [OVERLAP_SUP_LE7_SA_WKND_GE2] by [Age >=65] |
| 1017 | i_A2_MEQ_PER_DAY_SUPPLY_QTR_GE2 | [MEQ_PER_DAY_SUPPLY_QTR_GE200] by [Age 46-64] |
| 1018 | i_A3_MEQ_PER_DAY_SUPPLY_QTR_GE2 | [MEQ_PER_DAY_SUPPLY_QTR_GE200] by [Age >=65] |
| 1019 | i_A2_MEQ_PER_DAY_18MO_GE200 | [MEQ_PER_DAY_18MO_GE200] by [Age 46-64] |
| 1020 | i_A3_MEQ_PER_DAY_18MO_GE200 | [MEQ_PER_DAY_18MO_GE200] by [Age >=65] |
| 1021 | i_A2_MEQ_ER_PER_DAY_18MO_GE200 | [MEQ_ER_PER_DAY_18MO_GE200] by [Age 46-64] |
| 1022 | i_A3_MEQ_ER_PER_DAY_18MO_GE200 | [MEQ_ER_PER_DAY_18MO_GE200] by [Age >=65] |
| 1023 | i_A2_MEQ_PER_DAY_SUPPLY_18MO_GE | [MEQ_PER_DAY_SUPPLY_18MO_GE200] by [Age 46-64] |
| 1024 | i_A3_MEQ_PER_DAY_SUPPLY_18MO_GE | [MEQ_PER_DAY_SUPPLY_18MO_GE200] by [Age >=65] |
| 1025 | i_A2_PCT_CHANGE_MEQ_GE100_N_QTR | [PCT_CHANGE_MEQ_GE100_N_QTR_GE2] by [Age 46-64] |
| 1026 | i_A3_PCT_CHANGE_MEQ_GE100_N_QTR | [PCT_CHANGE_MEQ_GE100_N_QTR_GE2] by [Age >=65] |
| 1027 | i_A2_ER_RX_ANY | [ER_RX_ANY] by [Age 46-64] |
| 1028 | i_A3_ER_RX_ANY | [ER_RX_ANY] by [Age >=65] |
| 1029 | i_A2_ER_RX_COUNT_ANY_IN_QTR_GE2 | [ER_RX_COUNT_ANY_IN_QTR_GE2] by [Age 46-64] |
| 1030 | i_A3_ER_RX_COUNT_ANY_IN_QTR_GE2 | [ER_RX_COUNT_ANY_IN_QTR_GE2] by [Age >=65] |
| 1031 | i_A2_ER_RX_COUNT_SA_IN_QTR_GE2 | [ER_RX_COUNT_SA_IN_QTR_GE2] by [Age 46-64] |
| 1032 | i_A3_ER_RX_COUNT_SA_IN_QTR_GE2 | [ER_RX_COUNT_SA_IN_QTR_GE2] by [Age >=65] |
| 1033 | i_A2_TOTAL_QTRS_ER_RX_EVER_SA_G | [TOTAL_QTRS_ER_RX_EVER_SA_GE2] by [Age 46-64] |
| 1034 | i_A3_TOTAL_QTRS_ER_RX_EVER_SA_G | [TOTAL_QTRS_ER_RX_EVER_SA_GE2] by [Age >=65] |
| 1035 | i_A2_ER_RX_SUP_LE3_GE1 | [ER_RX_SUP_LE3_GE1] by [Age 46-64] |
| 1036 | i_A3_ER_RX_SUP_LE3_GE1 | [ER_RX_SUP_LE3_GE1] by [Age >=65] |
| 1037 | i_A2_ER_RX_SUP_LE7_GE2 | [ER_RX_SUP_LE7_GE2] by [Age 46-64] |
| 1038 | i_A3_ER_RX_SUP_LE7_GE2 | [ER_RX_SUP_LE7_GE2] by [Age >=65] |
| 1039 | i_A2_ER_RX_WKND_SUP_LE3_GE1 | [ER_RX_WKND_SUP_LE3_GE1] by [Age 46-64] |
| 1040 | i_A3_ER_RX_WKND_SUP_LE3_GE1 | [ER_RX_WKND_SUP_LE3_GE1] by [Age >=65] |
| 1041 | i_A2_ER_RX_WKND_SUP_LE7_GE1 | [ER_RX_WKND_SUP_LE7_GE1] by [Age 46-64] |
| 1042 | i_A3_ER_RX_WKND_SUP_LE7_GE1 | [ER_RX_WKND_SUP_LE7_GE1] by [Age >=65] |
| 1043 | i_A2_ER_RXSUP_ANY_GE6 | [ER_RXSUP_ANY_GE6] by [Age 46-64] |
| 1044 | i_A3_ER_RXSUP_ANY_GE6 | [ER_RXSUP_ANY_GE6] by [Age >=65] |
| 1045 | i_A2_ER_RXSUP_ANY_GE7 | [ER_RXSUP_ANY_GE7] by [Age 46-64] |
| 1046 | i_A3_ER_RXSUP_ANY_GE7 | [ER_RXSUP_ANY_GE7] by [Age >=65] |
| 1047 | i_A2_ANTID_TOTAL_QUARTERS_GE3 | [ANTID_TOTAL_QUARTERS_GE3] by [Age 46-64] |
| 1048 | i_A3_ANTID_TOTAL_QUARTERS_GE3 | [ANTID_TOTAL_QUARTERS_GE3] by [Age >=65] |
| 1049 | i_A2_ER_RX_a_EPI_OPIOID_BENZO_Y | [ER_RX_a_EPI_OPIOID_BENZO_YN] by [Age 46-64] |
| 1050 | i_A3_ER_RX_a_EPI_OPIOID_BENZO_Y | [ER_RX_a_EPI_OPIOID_BENZO_YN] by [Age >=65] |
| 1051 | i_A2_ER_RX_b_EPI_OPIOID_BENZO_Y | [ER_RX_b_EPI_OPIOID_BENZO_YN] by [Age 46-64] |
| 1052 | i_A3_ER_RX_b_EPI_OPIOID_BENZO_Y | [ER_RX_b_EPI_OPIOID_BENZO_YN] by [Age >=65] |
| 1053 | i_A2_ER_RX_a_EPI_OPIOID_HYPNO_Y | [ER_RX_a_EPI_OPIOID_HYPNO_YN] by [Age 46-64] |
| 1054 | i_A3_ER_RX_a_EPI_OPIOID_HYPNO_Y | [ER_RX_a_EPI_OPIOID_HYPNO_YN] by [Age >=65] |
| 1055 | i_A2_ER_RX_a_EPI_OPIOID_ANXIO_Y | [ER_RX_a_EPI_OPIOID_ANXIO_YN] by [Age 46-64] |
| 1056 | i_A3_ER_RX_a_EPI_OPIOID_ANXIO_Y | [ER_RX_a_EPI_OPIOID_ANXIO_YN] by [Age >=65] |
| 1057 | i_A2_ER_RX_b_EPI_OPIOID_ANXIO_Y | [ER_RX_b_EPI_OPIOID_ANXIO_YN] by [Age 46-64] |
| 1058 | i_A3_ER_RX_b_EPI_OPIOID_ANXIO_Y | [ER_RX_b_EPI_OPIOID_ANXIO_YN] by [Age >=65] |
| 1059 | i_A2_ER_DURING_BENZO_RX_COUNT_G | [ER_DURING_BENZO_RX_COUNT_GE2] by [Age 46-64] |
| 1060 | i_A3_ER_DURING_BENZO_RX_COUNT_G | [ER_DURING_BENZO_RX_COUNT_GE2] by [Age >=65] |
| 1061 | i_A2_RXSUP_QTR_ADD_GE11 | [RXSUP_QTR_ADD_GE11] by [Age 46-64] |
| 1062 | i_A3_RXSUP_QTR_ADD_GE11 | [RXSUP_QTR_ADD_GE11] by [Age >=65] |
| 1063 | i_A2_ER_DURING_BENZO_RX | [ER_DURING_BENZO_RX] by [Age 46-64] |
| 1064 | i_A3_ER_DURING_BENZO_RX | [ER_DURING_BENZO_RX] by [Age >=65] |
| 1065 | i_A2_OPIOID_ADD_OVERLAP_EVER | [OPIOID_ADD_OVERLAP_EVER] by [Age 46-64] |
| 1066 | i_A3_OPIOID_ADD_OVERLAP_EVER | [OPIOID_ADD_OVERLAP_EVER] by [Age >=65] |
| 1067 | i_A2_RXSUP_QTR_ANTIANXIETY_GE10 | [RXSUP_QTR_ANTIANXIETY_GE10] by [Age 46-64] |
| 1068 | i_A3_RXSUP_QTR_ANTIANXIETY_GE10 | [RXSUP_QTR_ANTIANXIETY_GE10] by [Age >=65] |
| 1069 | i_A2_HOMEOPATHIC_EVER | [HOMEOPATHIC_EVER] by [Age 46-64] |
| 1070 | i_A3_HOMEOPATHIC_EVER | [HOMEOPATHIC_EVER] by [Age >=65] |
| 1071 | i_A2_ANTIANXIETY_EVER | [ANTIANXIETY_EVER] by [Age 46-64] |
| 1072 | i_A3_ANTIANXIETY_EVER | [ANTIANXIETY_EVER] by [Age >=65] |
| 1073 | i_A2_ANTIANXIETY_TOTAL_QUARTERS | [ANTIANXIETY_TOTAL_QUARTERS_GE2] by [Age 46-64] |
| 1074 | i_A3_ANTIANXIETY_TOTAL_QUARTERS | [ANTIANXIETY_TOTAL_QUARTERS_GE2] by [Age >=65] |
| 1075 | i_A2_BENZODIAZEPINE_TOTAL_QUART | [BENZODIAZEPINE_TOTAL_QUART_GE3] by [Age 46-64] |
| 1076 | i_A3_BENZODIAZEPINE_TOTAL_QUART | [BENZODIAZEPINE_TOTAL_QUART_GE3] by [Age >=65] |
| 1077 | i_A2_MULTI_DRUGS_TOTAL_QUARTERS | [MULTI_DRUGS_TOTAL_QUARTERS_GE7] by [Age 46-64] |
| 1078 | i_A3_MULTI_DRUGS_TOTAL_QUARTERS | [MULTI_DRUGS_TOTAL_QUARTERS_GE7] by [Age >=65] |
| 1079 | i_A2_COUNT_ANXIO_WITH_OPIOIDS_G | [COUNT_ANXIO_WITH_OPIOIDS_GE3] by [Age 46-64] |
| 1080 | i_A3_COUNT_ANXIO_WITH_OPIOIDS_G | [COUNT_ANXIO_WITH_OPIOIDS_GE3] by [Age >=65] |
| 1081 | i_A2_EVER_POISONING_INJURY_DRUG | [EVER_POISONING_INJURY_DRUGS] by [Age 46-64] |
| 1082 | i_A3_EVER_POISONING_INJURY_DRUG | [EVER_POISONING_INJURY_DRUGS] by [Age >=65] |
| 1083 | i_A2_POST_INDEX_RX_TREAT_AA_EVE | [POST_INDEX_RX_TREAT_AA_EVER] by [Age 46-64] |
| 1084 | i_A3_POST_INDEX_RX_TREAT_AA_EVE | [POST_INDEX_RX_TREAT_AA_EVER] by [Age >=65] |
| 1085 | i_A2_EVER_ER_GE120_SA_GE80_IN_Q | [EVER_ER_GE120_SA_GE80_IN_QTR] by [Age 46-64] |
| 1086 | i_A3_EVER_ER_GE120_SA_GE80_IN_Q | [EVER_ER_GE120_SA_GE80_IN_QTR] by [Age >=65] |
| 1087 | i_A2_EVER_ER_GE120_SA_GT0_IN_QT | [EVER_ER_GE120_SA_GT0_IN_QTR] by [Age 46-64] |
| 1088 | i_A3_EVER_ER_GE120_SA_GT0_IN_QT | [EVER_ER_GE120_SA_GT0_IN_QTR] by [Age >=65] |
| 1089 | i_A2_EVER_ER_GE120_SA_GE60_IN_Q | [EVER_ER_GE120_SA_GE60_IN_QTR] by [Age 46-64] |
| 1090 | i_A3_EVER_ER_GE120_SA_GE60_IN_Q | [EVER_ER_GE120_SA_GE60_IN_QTR] by [Age >=65] |
| 1091 | i_A2_PCT_CHANGE_MEQ_GE50_N_QTR_ | [PCT_CHANGE_MEQ_GE50_N_QTR_GE2] by [Age 46-64] |
| 1092 | i_A3_PCT_CHANGE_MEQ_GE50_N_QTR_ | [PCT_CHANGE_MEQ_GE50_N_QTR_GE2] by [Age >=65] |
| 1093 | i_A2_ADD_EVER | [ADD_EVER] by [Age 46-64] |
| 1094 | i_A3_ADD_EVER | [ADD_EVER] by [Age >=65] |
| 1095 | i_M_MAX_N_OTHER_MEDS_AND_OPIOI | i_M_MAX_N_OTHER_MEDS_AND_OPIOI |
| 1096 | DX_30400_EVER | Binary flag indicating whether code code (indicated in predictor variable name) was ever present during period |
| 1097 | DX_30401_EVER | Binary flag indicating whether code code (indicated in predictor variable name) was ever present during period |
| 1098 | DX_30402_EVER | Binary flag indicating whether code code (indicated in predictor variable name) was ever present during period |
| 1099 | DX_30403_EVER | Binary flag indicating whether code code (indicated in predictor variable name) was ever present during period |
| 1100 | DX_30470_EVER | Binary flag indicating whether code code (indicated in predictor variable name) was ever present during period |
| 1101 | DX_30471_EVER | Binary flag indicating whether code code (indicated in predictor variable name) was ever present during period |
| 1102 | DX_30472_EVER | Binary flag indicating whether code code (indicated in predictor variable name) was ever present during period |
| 1103 | DX_30550_EVER | Binary flag indicating whether code code (indicated in predictor variable name) was ever present during period |
| 1104 | DX_30551_EVER | Binary flag indicating whether code code (indicated in predictor variable name) was ever present during period |
| 1105 | DX_30552_EVER | Binary flag indicating whether code code (indicated in predictor variable name) was ever present during period |
| 1106 | DX_30553_EVER | Binary flag indicating whether code code (indicated in predictor variable name) was ever present during period |
| 1107 | DX_96500_EVER | Binary flag indicating whether code code (indicated in predictor variable name) was ever present during period |
| 1108 | DX_96502_EVER | Binary flag indicating whether code code (indicated in predictor variable name) was ever present during period |
| 1109 | DX_96509_EVER | Binary flag indicating whether code code (indicated in predictor variable name) was ever present during period |
| 1110 | DX_96501_EVER | Binary flag indicating whether code code (indicated in predictor variable name) was ever present during period |
| 1111 | DX_30400_COUNT | Count of code (indicated in predictor variable name) during period |
| 1112 | DX_30401_COUNT | Count of code (indicated in predictor variable name) during period |
| 1113 | DX_30402_COUNT | Count of code (indicated in predictor variable name) during period |
| 1114 | DX_30403_COUNT | Count of code (indicated in predictor variable name) during period |
| 1115 | DX_30470_COUNT | Count of code (indicated in predictor variable name) during period |
| 1116 | DX_30471_COUNT | Count of code (indicated in predictor variable name) during period |
| 1117 | DX_30472_COUNT | Count of code (indicated in predictor variable name) during period |
| 1118 | DX_30550_COUNT | Count of code (indicated in predictor variable name) during period |
| 1119 | DX_30551_COUNT | Count of code (indicated in predictor variable name) during period |
| 1120 | DX_30552_COUNT | Count of code (indicated in predictor variable name) during period |
| 1121 | DX_30553_COUNT | Count of code (indicated in predictor variable name) during period |
| 1122 | DX_96500_COUNT | Count of code (indicated in predictor variable name) during period |
| 1123 | DX_96502_COUNT | Count of code (indicated in predictor variable name) during period |
| 1124 | DX_96509_COUNT | Count of code (indicated in predictor variable name) during period |
| 1125 | DX_96501_COUNT | Count of code (indicated in predictor variable name) during period |
| 1126 | DX_ANY_EVER | Any 304.*, 305.* or 965.* diagnosis code, ever. |
